# International meeting on "Molecular biology of DNA repair." Presented by the British Photobiology Society and DNA Repair Information Network. 16-18 April, 1986, Manchester. Abstracts of posters.

**DOI:** 10.1038/bjc.1986.184

**Published:** 1986-08

**Authors:** 


*Br. J. Cancer* (1986), **54**, 345–376

### International Meeting on "Molecular Biology of DNA Repair"

### Presented by the British Photobiology Society and DNA Repair Information Network (16–18 April, 1986)†

*Held at Owens Park, University of Manchester*

### Abstracts of Posters*

### A.1 Further studies on DNA repair in *Escherichia coli*

S.I. Ahmad

*Department of Life Sciences, Trent Polytechnic, Burton Street, Nottingham NG1 4BU, UK*

Two mutants of *Escherichia coli* showing enhanced resistance towards a number of DNA damaging agents have been isolated and characterised. One of them (SA236) is hyper-resistant to UV (the UVA and the UVB), mitomycin C, nalidixic acid, novobiocin, fluorouracil and thymineless death. However, it remains as sensitive as its parent strain for 8-methoxypsoralen (MPS) plus near UV. The other mutant (SA270) is hyper-resistant exclusively to MPS plus NUV.

SDS-PAGE analysis of SA270 showed that it is synthesising a protein of 55kd (perhaps a PUVA specific endonuclease) in higher concentrations than its parent strain.

Analysis of the enzyme activities of SA236 showed that certain enzymes of DNA repair pathways, noticeably DNA polymerase I, are synthesised in higher concentrations by the cell. This result and the results obtained by other experiments show that the synthesis of DNA polymerase I in *E. coli* is genetically controlled and that in a wild type cell, during normal growth conditions, the synthesis of this enzyme is repressed.

It is suggested that the hyper-resistance phenotype of the mutant bacteria is a consequence of hyper DNA repair ability of the cell towards DNA damage. Mutation affecting the UV-resistant phenotype in SA236 has been mapped near *argH* locus on the linkage map of *E. coli.*

### A.2 Induction of SOS and adaptive response by alkylating agents in *Escherichia coli* mutants deficient in 3-methyladenine-DNA glycosylase activities

S. Boiteux, R. Costa de Oliveira & J. Laval

*LA 147 CNRS and U 140 INSERM, Institut Gustave Roussy, Villejuif, France*

The induction of SOS and adaptive response by alkylating agents was studied in *E. coli* mutants *tagA* and *alkA* deficient in 3-methyladenine-DNA glycosylase (Tag) activities. The SOS response was measured using an operon fusion *sfiA*::*lacZ*. The *sfiA* operon in the double mutant *tagA alkA*, lacking both TagI and TagII, is induced at 5 to 50 fold lower concentrations of all tested methylating and ethylating compounds, as compared to the wild-type strains. The sensitization effect is mainly due to the *tagA* mutation which inactivates the constitutive and specific TagI. The sensitization effect of the *alkA* mutation, which inactivates the inducible TagII, is observed under conditions which allow significant induction of the adaptive response. Therefore, the persistance of 3-alkyladenine residues in DNA most likely leads to the induction of the SOS functions. In contrast, the adaptive response was not affected by either *tagA* or/and *alkA* mutations. The results suggest that SOS and adaptive response use different alkylation products as inducing signals. Although SOS and adaptation are distinct phenomena, *alkA* expression inhibits to some extent the induction of the SOS response due to its action on 3-alkyladenine residues. We provide conditions to improve short-term bacterial tests for the detection of genotoxic alkylating agents.

†Organisers: J.M. Boyle, Paterson Laboratories, Christie Hospital & Holt Radium Institute, Manchester M20 9BX; A. Collins & R.T. Johnson, Department of Zoology, University of Cambridge, Downing Street, Cambridge CB2 3EJ, UK.

*Oral presentations to be published as Supplement 6 to *Journal of Cell Science*—Ed.

© The Macmillan Press Ltd., 1986

### A.3 Complete nucleotide sequence of the *recB*, *recC* and *ptr* genes of *E. coli*

P.T. Emmerson[1], K. Brown[1], K.E. Chapman[1], P.W. Finch[1], I.D. Hickson[2], A. Storey[1], A.E. Tomkinson[1] & R.E. Wilson[1]

*Departments of [1]Biochemistry and [2]Clinical Oncology, The University, Newcastle-upon-Tyne NE1 7RU, UK*

We have sequenced a 14 kb region of the *E. coli* chromosome, which includes the 3′ end of the *thyA* gene, and the entire *recC*, *ptr* and *recB* genes. In the *thyA–recC* intergenic region, there are open reading frames which would code for proteins of 30 kd, 13.5 kd and 12 kd, and immediately downstream of *recB* is an open reading frame which would encode a protein of > 52 kd. There are no strong promoter sequences or LexA binding sites preceding the *recB* or the *recC* gene. This is consistent with the observation that the intracellular level of the RecBC enzyme is very low, due to inefficient transcription of both the *recB* and *recC* genes. Furthermore, the rate of transcription is not increased during the SOS response. The predicted amino acid sequence of the RecB protein, but not that of the RecC protein, contains a consensus adenine-binding site, which correlates with the observation that only the RecB protein has a DNA-dependent ATPase activity. The sequence downstream of *recB* appears to be required for maximal expression of RecBC DNase activity and may encode the 60 kd protein reported by Lieberman and Oishi [*Proc. Natl Acad. Sci.* **71**, 4816, 1974].

### A.4 Analysis of the regulatory elements of the *Escherichia coli uvrC* gene by construction of operon fusions.

J.W. Forster[1] and P. Strike[2]

*[1]Department of Agricultural Botany, University College of Wales, Aberystwyth, Dyfed SY23 3DD and [2]Department of Genetics, University of Liverpool, Brownlow Street, Liverpool L69 3BX, UK*

The *E. coli uvrA*, *uvrB* and *uvrC* genes encode proteins involved in the early steps of excision repair of a variety of noncoding lesions in DNA, the best studied being UV-induced thymine dimers. The three gene products act as a complex, incising duplex DNA on either side of the site of damage. The *uvrA* and *uvrB* genes have been shown to be induced in response to DNA damaging agents as part of the cellular SOS system. In order to investigate the regulation of the *uvrC* gene, we have cloned the gene in multicopy plasmid vectors. By subcloning restriction fragments from the promoter proximal region of the *uvrC* gene into the promoter probe vector pPV502, we have constructed a series of operon fusions to the chloramphenicol acetyltransferase gene. This study has allowed us to detect and quantify the activity of multiple promoters in the *uvrC* control region. The regulation of *uvrC* is apparently complex and differs from that of *uvrA* and *uvrB*.

### Evidence for increased DNA repair synthesis in *Escherichia coli* strains containing plasmids pKM101 or pGW16 following UV-irradiation

C.A. Little[1], D.J. Tweats[2] & R.J. Pinney[1]

*[1]Microbiology Section, The School of Pharmacy, London WC1N 1AX, [2]Glaxo Group Research Ltd., Ware, Hertfordshire SG12 0DJ, UK*

Plasmid pKM101 increases survival and mutagenesis after DNA damage. It carries *mucAB* genes that are analogous to *umuDC*, chromosomal error-prone DNA repair genes of *E. coli.* Plasmid pGW16, a derivative of pKM101, increases DNA damage-induced mutagenesis more than pKM101.

DNA synthesis was measured in growing cells of *E. coli* strain AB1157 *umuC*$^+$ and in strain TK702 *umuC* as the amount of [$^3$H]-thymidine incorporated into acid-insoluble material. Plasmids pKM101 and pGW16 increased post-UV DNA synthesis, particularly in strain TK702, with pGW16 having the greater effect despite giving lower protection against UV than pKM101. This is further evidence that pGW16 contains a mutation in the *mucAB* regulatory region, and suggests that increased DNA-repair synthesis is involved in error-prone DNA repair.

### A.6 Cloning of the *Micrococcus luteus* 3-methyladenine-DNA glycosylase gene in *Escherichia coli*

J. Pierre & J. Laval

*LA 147-CNRS U 140 INSERM, Institut Gustave-Roussy, 94805 Villejuif Cédex, France*

Upon alkylation of DNA by chemical carcinogens such as dimethyl sulfate and methylmethane sulfonate, the main reaction products are 7-methylguanine and 3-methyladenine. In the case of methylnitrosourea and methylnitrosoguanidine, in addition to the above products, $O^6$-methylguanine and phosphodiesters are formed. The 3-methyladenine DNA glycosylase excises the 3-methyladenine residues formed in DNA after treatment with alkylating agents. In *E. coli*, the repair of this lesion depends on the product of the genes *tagA* and *alkA* which code for 3-methyladenine-DNA glycosylase I and II respectively. The *tagA* or *alkA* mutants are very sensitive to alkylating agents. We have cloned two genes of *M. luteus* that can partially substitute for the function of the *E. coli* *tagA*$^-$and *alkA*$^-$ genes. An *M. luteus* genome bank was made by shotgun cloning of *Ecor*RI + *Bam*HI digested DNA into pBR322. Two hybrid plasmids were identified that conferred MMS resistance to the *tagA*$^-$ mutant and a capacity to reactivate MMS treated bacteriophage $\lambda$. Each hybrid plasmid directed the synthesis of 21-kd 3-methyladenine-DNA glycosylases in *E. coli tagA*$^-$, which were not inhibited by 4 mM 3-methyladenine. However, the restriction maps of the two cloned genes were different, and they showed no sequence homology as judged by the lack of cross hybridization.

(This work was supported by grant from CNRS, INSERM (CRL 82 2027) and Association pour la Recherche sur le Cancer, Villejuif.)

### A.7 Cloning and expression of *M. luteus* repair functions in *E. coli*

S. Riazuddin, B. Chaudhry & A. Athar

*CAMB, University of the Punjab, Lahore, Pakistan*

Wild type *M. luteus* cells have been adapted by a stepwise treatment with sublethal concentrations of N-methyl-N-Nitro-N-Nitrosoguanidine (MNNG). The adapted cells exhibit 5.7 fold increased resistance to the killing effects of the mutagen and simultaneous efficient removal of various modified bases present in the cellular DNA. Three distinctly different repair proteins present in adapted cell extracts have been resolved by Sephadex G-75 chromatography and tentatively designated as enzymes I, II and III in order of their elution. Enzymes I and II correct $O^6$-MeG and $O^4$-MeT respectively by demethylation whereas enzyme III is a DNA glycosylase with absolute specificity for $O^2$-MeT. There is no observed cross specificity between the catalytic functions of the three repair proteins. All three enzymes are absent in wild type *M. luteus* or *ada*$^-$ *E. coli*. Regulatory genes of the isolated enzymes have been cloned and expressed in *ada*$^-$ *E. coli* by ligating chromosomal DNA partials digested with Sau3AI into the vector pBR322. *E. coli ada*$^-$ cells, transformed with hybrid vector, show increased resistance to the killing effects of MNNG when compared with the untransformed parent cells. Some of these transformants exhibit constitutive synthesis of $O^2$-MeT, $O^4$-MeT and $O^6$-MeG repair functions. Data on cloning and analysis of transformants will be presented.

### A.8 The influence of pre-irradiation growth temperature on the photoreactivable responses of UV irradiated dark repair deficient mutants of *Escherichia coli* K-12

A.W. Smith & S.H. Moss

*School of Pharmacy and Pharmacology, University of Bath, Claverton Down, Bath BA2 7AY, UK*

It has been shown that reducing the growth temperature increases the number of photoreactivating enzyme molecules in cells of *Saccharomyces cerevisiae* (Fukui & Laskowski: *Photochem. Photobiol.* **39**, 613). As part of our investigation into the genetic control of photoenzymatic repair in *Escherichia coli* K-12 we have compared the photoreactivable responses of totally dark repair deficient strains after growth at 37°C and 26°C. In addition to using the photoreactivation proficient strain AB2480 (*uvrA*, *recA*) we have also tested a *phrB* strain DY326 (*uvrA*, *uvrB*, *recA*, *phrB*), and a deletion mutant at the (*gal-chlA*) interval which includes *uvrB* and the putative *phrA* gene, strain AS44 ($\Delta$(*gal-chlA*), *recA*). The number of photoreactivating enzyme molecules was estimated by the high intensity msec flash technique and after continuous illumination. The photoreactivable response of these three strains was increased when grown at 26°C compared to growth at 37°C after 254 nm UV treatment. The phr$^+$ and the $\Delta$(*gal-chlA*) strains showed an ~2-fold increase in the initial rate of repair. After growth at 37°C, photoreactivation in the *phrB* mutant proceeds at a rate at least 100 fold lower than either the phr$^+$ or $\Delta$(*gal-chlA*) strains. However, a 4-fold increase in rate is seen when grown at 26°C.

### A.9 Post-UV kinetics of *recB*-dependent repair: relationship to post-UV inactivation of the prophage

Ž. Trgovčević, D. Petranović, E. Salaj-Šmic & M. Petranović

*Institute 'Rugjer Bošković', Zagreb, Yugoslavia*

We report the studies which were undertaken to investigate a possible relationship between *recB*-dependent repair of the bacterial chromosome and *recB*-dependent prophage inactivation. By making use of the temperature-sensitive *recB270* mutant, we were able to determine post-UV kinetics of the *recB*-dependent recovery of the cell viability (i.e., of the repair of the bacterial chromosome). By making use of the heat-inductibility of the *λcIts857indl*-lysogens, we were able to determine post-UV kinetics of the prophage inactivation. The results suggest that the RecBC enzyme has two opposite effects on repair. It reactivates a considerable fraction of damaged cells, whereas it inactivates the prophage in those lysogens whose DNA cannot be successfully repaired.

### A.10 The SOS-like system of *Bacillus subtilis*: a role for inducible repair in differentiaion

R.E. Yasbin & P.E. Love

*Microbiology Department, University of Rochester, Rochester, NY, USA*

DNA damage-inducible (Din) operon fusions were generated in *Bacillus subtilis* by transpositional mutagenesis. These Din fusions demonstrated increased transcriptional activity when exposed to UV, mitomycin C or ethyl methanesulfonate. One of the fusion strains was DNA repair deficient and was found to map with the *uvrA*$^+$ loci. Transcriptional activation of these strains also occurred when the bacteria differentiated into their competent state. Both the DNA-damage inducible and competence inducible components were abolished by the *recE4* mutation, which inhibits SOS-like (SOB) induction but does not interfere with the development of the competent state. A plasmid that expresses the *E. coli* RecA protein restored DNA repair capacity, recombination capability and induction of some of the SOB phenomena in a *recE4* mutant of *B. subtilis*. In response to treatment with agents that damage cellular DNA, *E. coli* RecA protein induced *Din* operon expression, W-reactivation and synthesis of *B. subtilis* recombinant protein (Recbs) that is analogous to RecA, but was unable to stimulate prophage induction. In addition, the RecA protein was capable of inducing the SOB response in competent *recE4* strains, independent of exposure to DNA damaging agents.

### B.1 The sensitivities of radiation sensitive *Saccharomyces cerevisiae rad* mutants to a range of monofunctional alkylation agents

A.J. Cooper & R. Waters

*School of Biology, University College, Swansea, UK*

Data will be presented which show the following sensitivities of *rad* strains to monofunctional alkylating agents.

Summary of the *rad* mutant sensitivities to alkylating agents:

| *Strain* | *DES* | *EMS* | *ENU* | *ENNG* | *MNU* |
|---|---|---|---|---|---|
| a *rad1-1* | S | R | S | S | R |
| a *rad2-1* | S | R | S | S | R |
| α *rad3-2* | R | R | R | — | R |
| a *rad4-4* | R | R | S | S | R |
| a *rad10-1* | R | R | R | R | — |
| α *rad14-2* | R | R | S | S | R |
| a *rad16-1* | R | R | R | — | — |
| α *rad6-1* | S | S | S | S | S |
| α *rad8-1* | R | R | S | S | — |
| α *rad9-1* | S | R | — | S | S |
| a *rad18-2* | — | — | S | S | S |
| a *rad50-1* | S | — | S | — | — |
| α *rad53-1* | S | — | S | — | — |
| a *rad54-1* | S | — | S | — | — |

### B.2 Selecting human and hamster variant cell lines by replica plating

A. Collins, R.T. Johnson & I. Rasko

*CRC Mammalian Cell DNA Repair Group, Department of Zoology, University of Cambridge, Downing Street, Cambridge CB2 3EJ, UK*

"Replica plating" describes the production of multiple copies of a pattern of colonies derived from single cells. The process allows the selection of mutant colonies whose identification involves an assay which kills the cells; the chosen cell clone can be retrieved from its live counterpart on the replica. Only recently has a reliable method been published for the replica plating of hamster cells, using polyester mesh. I have now adapted the technique for use with permanent human cell lines. At least four good replicas of a given set of colonies can be obtained, using cell lines of widely differing morphologies.

I describe the protocol with which we have isolated UV-sensitive hamster cell lines from a mutagenised population. The protocol can be readily adapted to look for mutants sensitive to other DNA-damaging agents. Replica plating can also be used in the selection of resistant phenotypes (e.g. after DNA transfection of sensitive mutants), avoiding problems associated with the selection of a resistant subpopulation by continual exposure to the genotoxic agent. Furthermore, DNA from colonies on a polyester replica can be readily transferred to nitrocellulose which can be processed as an autoradiogram. So replicas may be screened using assays that depend on radioactive tracers and a colony bearing a particular gene may be identified by hybridisation with a suitable labelled DNA probe.

### B.3 Study of the induction of genetic damage by X-rays using repair-deficient mutants of Drosophila melanogaster.

W. Ferro and G.B. Vegt

*Department of Radiation Genetics and Chemical Mutagenesis, State University of Leiden, The Netherlands*

The recovery of X-ray induced genetic damage in *Drosophila melanogaster* can be modified by the use of mutants deficient in the repair of UV-induced DNA damage. From the characteristics of the affected genetic endpoints inferences can be made about the DNA lesions that are involved. We now try to identify these DNA lesions biochemically in primary cell cultures derived from embryos. A genetic effect of *mus-101* (post-replication repair deficient for UV damage) is to reduce strongly the frequency of translocations. This points to a deficiency in the repair of DNA strand breaks. The repair of DNA break damage was followed in primary cell cultures by means of the alkaline unwinding technique. After biologically relevant doses of X-rays (20 Gy) no difference in repair could be detected between *mus-101* and the control strain. This means that the genetic effect of *mus-101* is not caused by a deficiency in the closing of a sizeable part of the DNA breaks.

### B.4 Introduction of *E. coli ada* gene into Mex⁻ human cells

H. Kataoka, J. Hall & P. Karran

*Imperial Cancer Research Fund, Clare Hall Laboratories, Potters Bar, Herts EN6 3LD, UK*

Human $Mex^-$ MRC5VA cells and CHO cells have been transfected with plasmids which are mammalian expression vector pSV2gpt derivatives carrying the *E. coli ada* gene. pHJ2 has a 1.3 kb insert of *E. coli* DNA, which contains the whole *ada* structural gene and its own promoter. The *ada* gene encodes a 39 kd protein which has both $O^6$-methylguanine-DNA methyltransferase and methyl phosphotriester-DNA methyltransferase activities. pHJ24 is derived from pHJ2 by introducing a frameshift mutation to make a stop codon about 0.3 kb away from the start of the *ada* structural gene and expresses an *Ada* protein fragment which has only phosphotriester DNA methyltransferase activity. pHJ53 has a 1.0 kb fragment of the *ada* structural gene, containing the active site for $0^6$-methylguanine repair.

These plasmids have been introduced into $Mex^-$ cells by calcium phosphate transfection together with a plasmid containing cloned mouse dhfr cDNA. After selecting $gpt^+$ colonies for resistance to mycophenolic acid, transformants have been treated with methotrexate to amplify the foreign DNA sequences. Mycophenolic acid, methotrexate resistant colonies are being examined for their $O^6$-methylguanine and phosphotriester repair capacities.

### B.5 New X-ray-sensitive mutants of cultured hamster cells

N.J. Jones, P.G. Debenham & J. Thacker

*MRC Radiobiology Unit, Chilton, Didcot, Oxon OX11 ORD, UK*

Few mutants have been isolated on the basis of their sensitivity to ionising radiations. Using a replica microwell technique, we have now isolated 4 X-ray-sensitive mutants of V79 hamster cells after screening 5000 ENU-treated clones. Three of these mutants show similar increased sensitivities, although the shapes of their survival curves differ, while the remaining mutant is only slightly more sensitive to X-rays than the wild type. These mutants also show different patterns of sensitivity to other agents (UV, EMS, mitomycin C). Complementation by cell fusion of double-marked mutant and wild type cells has shown that the 3 more sensitive mutants are in different complementation groups (CGs). Further, at least 2 of these mutants are in different CGs from X-ray-sensitive mutants isolated by others (*xrs*1, EM7-2). Thus we have defined at least 4 CGs for X-ray sensitivity in hamster cells.

DNA double-strand break repair was assessed by the ability of mutant and wild type cells to rejoin restriction endonuclease cuts in a transferred recombinant gene. One of the 2 mutants examined to date is significantly reduced in ability to correctly rejoin such double-strand damage.

The feasibility of molecular cloning of the genes complementing radiation sensitivity is being assessed in the same 2 mutants. The amount of high molecular weight human DNA integrated by the recipient cells is limited, making selection difficult.

B.6 **Reversion of a defect in DNA repair induced at high frequency by azacytidine**

P.A. Jeggo & R. Holliday

*Genetics Division, National Institute for Medical Research, The Ridgeway, Mill Hill, London NW7 1AA, UK*

Six X-ray sensitive strains of the CHO cell line, which all have a defect in double strand break rejoining have been shown to revert to the X-ray resistant phenotype at high frequency after treatment with azacytidine. The revertants are stable, but do not necessarily have the wild type level of resistance to X-irradiation. The azacytidine treatment has been shown to strongly decrease the level of DNA methylation, and the results suggest that the *xrs* repair gene is under methylation control in CHO cells. Since all 6 *xrs* strains revert at high frequency, and since the strains were obtained after treatment with the powerful mutagen, EMS and have somewhat different phenotypes, we propose a model that the strains are of mutational origin but that the CHO parent line carries a silent copy of the *xrs* gene inactivated by DNA methylation. This silent copy is reactivated by azacytidine treatment. Furthermore, we suggest that hypomethylation may be one explanation for the functional hemizygosity observed in cultured cell lines.

B.7 **Cloning of human DNA repair genes: 1. Immortalisation of primary strains. 2. Gene transfer and selection for resistance to DNA damaging agents**

L.V. Mayne[1], A. Priestley[2], T. Jones[1], & C.F. Arlett[2]

[1]*Cell and Molecular Biology Laboratory,* [2]*MRC Cell Mutation Unit, University of Sussex, Falmer, Brighton, Sussex, UK*

We have used a plasmid (pSV3*gpt*) containing both the SV40 early region encoding T antigen and the bacterial gene xanthine-guanine phosphoribosyl transferase (*gpt*) to achieve high efficiency morphological transformation and immortalization of primary human skin fibroblasts. Transfection of this plasmid into primary human skin fibroblasts derived from a normal individual, two Cockayne's syndrome patients (CS), an ataxia-telangiectasia patient and an immuno-deficient patient (46BR) followed by selection for the *gpt* gene resulted in an altered cell morphology and growth properties characteristic of previously described SV40 transformed cells. Transfected cultures subsequently senesced, entered crisis and in each case formed a rapidly growing culture.

We are attempting to clone the defective gene in CS and 46BR. Normal human DNA is extracted, partially digested with MBO I and ligated to pSV2*neo*. The DNA is transfected into immortalised CS and 46BR cells using the calcium phosphate technique. Transfectants are first selected by growth in the antibiotic G418 (bacterial *neo* gene confers resistance to G418) and then submitted to a regime of DNA damage designed to kill sensitive cells while enriching for resistant cells.

B.8 **Characterisation of mitomycin-C sensitive mutants of CHO-K1 cells and their use as hosts for the cloning of human DNA repair genes**

C.N. Robson, A.L. Harris & I.D. Hickson

*Cancer Research Unit, University of Newcastle upon Tyne, Royal Victoria Infirmary, Newcastle upon Tyne NE1 4LP, UK*

We have isolated 10 CHO-K1 cell lines (designated MMC-1 to 10) which exhibit greater than 5-fold sensitivity to the cytotoxic effects of mitomycin-C. Despite exhibiting similar levels of sensitivity to mitomycin-C, these mutants differ in the cross-sensitivity patterns to other DNA damaging agents, such as UV light, *cis*-Pt, chlorambucil and melphalan. Only one of the mutants (MMC-2) is hypersensitive to UV light, which correlates with the finding that MMC-2 is also unique in its sensitivity to decarbomyl mitomycin-C, the monofunctional derivative of mitomycin-C. We are currently studying the repair of DNA cross-links in these mutants using alkaline elution.

Analysis of hybrids generated by fusing all combinations of MMC-1 to 5, and wild-type cells, shows that these 5 mutants are phenotypically recessive and that they represent at least 4 different genetic complementation groups. Complementation analysis with the remaining 5 mutants is in progress.

We have constructed a human gene bank in the selectable marker cosmid pNNL (Eco*gpt*). This DNA has been transfected into the mutants and transformants selected which exhibited a repair-competent phenotype. In 2 cases, MMC-1 and MMC-4, transformants exhibiting wild-type level of mitomycin-C resistance have been recovered. These lines have stably integrated *gpt* and human DNA sequences (by Alu hybridisation). We are currently attempting to recover the transfected DNA using marker rescue techniques.

### B.9 DNA repair chracteristics of Walker tumour cells sensitive or resistant to difunctional agents

J.J. Roberts, R.J. Knox & D.A. Lydall

*Department of Molecular Pharmacology, Institute of Cancer Research, Sutton, Surrey SM2 5PX, UK*

The Walker 256 carcinoma cell (WS) is inherently sensitive to difunctional agents. Resistant Walker cells (WR) show comparable sensitivity to conventional cell lines. Both sensitive and resistant lines have the same ability to remove DNA bound platinum adducts and to circumvent DNA adducts during replication. There is however a marked difference in the time course of the inhibition of DNA synthesis due to the failure of WS cells to recover from the early inhibition of DNA synthesis induced by difunctional agents. Both WS and WR cell lines are transfectable with pSV2gpt and pSV2neo plasmids in suspension culture. Using this system no difference has yet been detected between the cell lines in their response to specific, defined, damage induced into the plasmid probes, prior to transfection, by various restriction endonucleases. Also both cell lines show equal inhibition of their transfection rate with increasing platination of a SV2gpt probe. This is consistent with there being a deficiency in a late step in the repair of a rare lesion in DNA, such as an interstrand crosslink, in the DNA of WS cells although the basis of this defect has not yet been defined.

### B.10 Chracterisation of mutants of CHO-K1 cells which exhibit sensitivity to drugs whose cytotoxicity is mediated via topoisomerase II

C.N. Robson, A.L. Harris & I.D. Hickson

*Cancer Research Unit, University of Newcastle upon Tyne, Royal Victoria Infirmary, Newcastle upon Tyne NE1 4LP, UK*

We have previously described the isolation of 2 CHO mutants (BLM-1 and 2) which are hypersensitive to killing by bleomycin [*Cancer Res.* **45,** 5304, 1985]. These mutants were subsequently found to exhibit sensitivity to adriamycin and VP16, 2 of the drugs thought to exert their cytotoxic effects by interfering with the action of topoisomerase II.

We have recently isolated an additional mutant, designated ADR-1 which is 7-fold sensitive to adriamycin and VP16 but, unlike BLM-1 and 2, is also hypersensitive to all classes of intercalating agents, including mAMSA, ellipticine and mitoxantrone. ADR-1 is not sensitive to radiation or to mono- and bi-functional alkylating agents, suggesting that it is mutated for a gene product acting specifically in a topoisomerase II-dependent reaction.

Although adriamycin and VP16 are 2 of the drugs associated with the multi-drug resistant phenotype, there is no evidence that the "reverse" of this *p*-glycoprotein mediated drug resistance mechanism is operating in these mutants, particularly as no sensitivity to vincristine or melphalan is observed.

We intend to measure the rate of appearance and repair of DNA strand breaks induced by intercalating agents in these cell lines. We are also comparing the activity of topoisomerase II isolated from ADR-1 cells with that from wild-type cells.

### B.11 SVM, a UV-sensitive muntjac cell line with a high rate of sister chromatid exchange and a defect in post-irradiation replication recovery

S.R.R. Musk, L. Pillidge, C.S. Downes & R.T. Johnson

*Cancer Research Campaign Mammalian Cell DNA Repair Group, Department of Zoology, Cambridge CB2 3EJ, UK*

Cells of the Indian muntjac, *Muntiacus muntjak*, are convenient for chromosome studies since they have very few, large chromosomes ($2n=7$ in male, 6 in female). We have found that an SV40-transformed line, SVM, has an unusually high rate of UV-induced chromosome aberrations and a twentyfold enhancement of sister chromatid exchanges, which can be detected after doses as low as $0.01\,Jm^{-2}$. SVM cells are also hypersensitive to UV killing. Their capacity for excision repair of UV damage is no worse than in non-transformed muntjac cells of normal sensitivity to UV; but after irradiation they have a defect in the recovery of rates of DNA or RNA synthesis, and show a reduced rate of maturation of DNA into high molecular weight material. This defect is partly analogous with the human xeroderma pigmentosum variant.

B.12 **Lack of correlation between excision repair of UV damage and adenovirus reactivation in an XP(D)-like cell line.**

R.T. Johnson[1], S. Squires[1], G.C. Elliott[1], & A.J. Rainbow[2]

[1]*Cancer Research Campagin Mammalian Cell DNA Repair Group, Department of Zoology, University of Cambridge, UK;* [2]*Departments of Radiology and Biology, McMaster University, Hamilton, Ontaria, Canada*

Hybrids formed between HeLa cells and fibroblasts from xeroderma pigmentosum group D show either HeLa sensitivity or XPD-like hypersensitivity to ultraviolet radiation and corresponding high or low excision repair capability. Hybrids with low repair are judged to have lost, via chromosome segregation, the HeLa wild type D alleles. Here we analyse the UV sensitivity and excision repair capability of another hybrid, HD1A, derived spontaneously from hybrid HD1 (described previously by Johnson *et al., J. Cell Sci.* **76** 115, 1985). While HD1A closely resembles the XPD phenotype in terms of UV sensitivity and excision repair it differs from XPD because of its ability to reactivate UV irradiated adenovirus 2 to an extent similar to that of its HeLa parent. This capacity functionally dissociates excision repair of chromatin-based damage from damage in a viral environment. Moreover, on the basis of complementation studies the excision repair of genomic damage by HD1A is subtly different from that of a true XPD-like hybrid, HD2.

C.1 **The induction of SOS-phenomena in normal and repair deficient human cells after UV-treatment**

P.J. Abrahams, A.A.M. van der Kleij & A.J. van der Eb

*Department of Medical Biochemistry, State University of Leiden, P.O. Box 9503, 2300 RA Leiden, The Netherlands*

We have investigated the occurrence of SOS-phenomena such as Enhanced Reactivation (ER) and Enhanced Mutagenesis (EM) of Herpes Simplex Virus type 1 (HSV-1) after UV-treatment of normal fibroblasts and cells from the following repair syndromes: Xeroderma Pigmentosum (XP), Ataxia Telangiectasia (AT), Bloom's Syndrome (BS) and Cockayne Syndrome (CS). ER and EM followed similar kinetics in normal and in XP cells from complementation groups A, C and D. Maximum activities occurred when infection with HSV-1 was delayed 1–2 days after UV-treatment. However, certain XP strains did not express an ER phenomenon, whereas the EM response was normal. Interestingly, these latter XP cells originated from patients that were reportedly (still) free from cancer in sunlight-exposed skin areas.

In BS and CS the ER and EM responses were both expressed, following similar kinetics to normal cells. However, the ER-response in BS cells was unusually high. In AT cells the EM-response was unusually low, whereas ER was normal.

These results suggest that SOS-phenomena are transiently expressed in normal and repair deficient cells and that the ER response positively correlates with cancer induction.

C.2 **Comparative study on the repair of 4,5′,8-trimethylpsoralen (TMP) plus UVA induced DNA crosslinks in a normal and a Fanconi's anemia cell line.**

D. Averbeck & D. Papadopoulo

*Institut Curie—Biologie, Paris, France*

We observed that human fibroblasts from Fanconi's anemia patients (FA) were only ~2 times more sensitive than normal human fibroblasts to the inhibition of colony forming ability by a DNA crosslinking treatment with 8-methoxypsoralen and UVA. Using the more photoreactive bifunctional furocoumarin TMP and UVA, we show that the difference in sensitivity between a normal human fibroblast (1BR/3) and a FA cell line (FA 150) was a factor of 2 higher than that observed after treatment with 8-MOP and UVA.

Since TMP photoinduces more crosslinks per unit dose than 8-MOP we asked the question whether the increased sensitivity of FA cells to TMP plus UVA treatments could be due to the induction of relatively higher amounts of DNA crosslinks and to a deficiency in repair. Knowing that at different wavelengths and with suitable combinations thereof the ratio of crosslinks (CL) over monoadducts (MA) induced by TMP can be changed, a sun lamp equipped with a monochromator was used to enhance the ratio of CL/MA at a given level of total lesions photoinduced by TMP. Experiments carried out with alkaline elution show that under these conditions in the normal fibroblast cell line ~80% while in the FA cell line only ~35% of the DNA interstrand crosslinks are repaired during 24 h of post-treatment incubation.

**C.3 The establishment and characterisation of a Xeroderma pigmentosum cell line transformed by an origin-defective SV40 recombinant plasmid**

L. Daya-Grosjean, M. James, C. Drougard & A. Sarasin

*Laboratoire de Mutagenèse Moléculaire, IRSC, 7, rue Guy Môquet, 94800 Villejuif, France*

Xeroderma pigmentosum (XP) is an autosomal recessive disease in which DNA repair processes are defective. Patients with this disease develop multiple skin lesions culminating in skin carcinomas and early death.

We have established permanent cell lines from foetal XP cells (Group C) and normal human foetal fibroblasts.

Transformation was carried out with a recombinant plasmid, pLAS-wt, containing SV40 DNA encompassing the entire early region with a defective origin of DNA replication. The transformed XP cell line, XP4PA-SVwt, and the normal transformed fibroblasts AS3-SVwt, both express SV40 T antigen together with enhanced levels of the transformation-associated cellular protein, p53. XP4PA-SVwt retains the XP UV-repair defective phenotype as demonstrated by low levels of unscheduled DNA synthesis and by the reduced survival of irradiated SV40 virus. Analysis of cellular DNA shows a single major, stable, integration site of pLAS-wt in the XP4PA-SVwt cells. The T antigen in these cells supports efficiently the replication of SV40 based shuttle vectors and should prove suitable for the introduction, expression and selection of genes related to DNA repair.

**C.4 DNA-mediated gene transfer of human wild type gene(s) into Fanconi's anemia (FA) fibroblasts**

C. Diatloff-Zito, D. Papadopoulo, D. Averbeck & E. Moustacchi

*Institute Curie—Biologie, Paris, France*

DNA from wild type human cells was transfected into FA primary skin fibroblasts along with UV-irradiated pSV2*neo* plasmid and transformants were tested for correction of hypersenstivity of the cells to mitomycin C (MMC) and for the rate of semi-conservative DNA synthesis after 8-Methoxypsoralen (8-MOP) plus UVA. The strategy used consists of (i) a preselection of a primary FA cell line competent for transformation with the neo gene, (ii) a selection procedure of the MMC resistant cells which takes advantage of the higher proliferation rate and plating efficiency of the MMC resistant transformants as opposed to the slow growing cells (FA phenotype).

Transformants were obtained that demonstrate a normal resistance to MMC in terms of clonogenic cell survival, a recovery of a normal pattern of DNA semi-conservative synthesis after 8-MOP+UVA and the presence of exogenous pSV2*neo* plasmid DNA sequences in the cells. The frequency of transfer of the MMC resistant character lies between $3.10^{-6}$–$10^{-7}$ as estimated from reconstruction experiments. Sensitivity to MMC was maintained when FA cells were mock transfected or transfected with their own DNA, with yeast or salmon sperm DNA. Thus it is unlikely that selection of spontaneous MMC resistant revertants accounts for restitution of the MMC resistance by transfection with normal DNA.

**C.5 Analysis of the fate of 8-MOP plus UVA induced DNA crosslinks in normal and Fanconi's anemia fibroblasts and lymphoblasts**

D. Papadopoulo, D. Averbeck & E. Moustacchi

*Institute Curie—Biologie, Paris, France*

To find out whether the defect associated with Fanconi's anemia (FA) might involve a defect in the repair of DNA interstrand crosslinks (CL), the colony forming ability and the fate of CL were studied in normal and FA fibroblasts following 8-Methoxypsoralen (8-MOP) photoaddition. FA cells belonging to complementation groups A and B were found to be more sensitive than normal fibroblasts by a factor of 2. The possible repair of CL was followed using the alkaline elution technique.

After 20 h of post treatment incubation the elution rate of initially crosslinked DNA was increased in all cell lines. However difference in the kinetics were seen between normal and FA cells. FA fibroblasts and lymphoblasts from complementation group A showed slower repair kinetics than normal cells and those of complementation group B showed a response closer to normal. Although FA cells show incision of CL, they demonstrate a reduced capacity of the repair of 8-MOP photo-induced CL.

### C.6 Detection of ataxia-telangiectasia heterozygotes by chromosomal radiosensitivity: A "blind study"

Y. Shiloh[1], R. Parshad[2], K.K. Sanford[3] & G.M. Jones[3]

[1]*Department of Human Genetics, Sackler Faculty of Medicine, Tel Aviv University, Ramat Aviv 69978, Israel;* [2]*Department of Pathology, Howard University College of Medicine, Washington, D.C. 20059, USA;* [3]*Laboratory of Cellular & Molecular Biology, National Cancer Institute, Bethesda, MD 20892, USA*

Ataxia-telangiectasia (A-T) is an autosomal recessive disease characterized by cerebellar degeneration, immunodeficiency, chromosomal breakage, radiosensitivity and extreme proneness to lymphoid malignancies. A laboratory assay for the identification of A-T heterozygotes is essential both for genetic counselling and linkage studies aimed at mapping the A-T gene. Fibroblast cell lines from obligatory A-T heterozygotes show an intermediate sensitivity to X-ray cytotoxicity, however, occasional overlaps with the normal sensitivity range precluded the use of this phenomenon as a reliable diagnostic assay.

We tested the possibility of using sensitivity to chromatid damage at the $G_2$ phase of the cell cycle for carrier detection in A-T, in a "blind" fashion. Thirteen coded fibroblast lines from A-T patients, obligatory A-T heterozygotes and healthy controls were tested after irradiation with 1 Gy of X-rays. Based on the results, the cell lines could be classified as showing either "high" or "low" X-ray sensitivity. When the code was broken it was found that only cells from healthy controls fell into the low sensitivity range, while both A-T homozygous and heterozygous cells showed the high sensitivity. Thus all the healthy individuals in the latter group were correctly identified as carriers of the A-T gene. The use of this assay, should, however, be limited to members of A-T families because of the large variability in X-ray sensitivity in the general population.

### C.7 Abnormal response of ataxia-telangiectasia cells to a topoisomerase II-interactive drug (epipodophyllotoxin)

P.J. Smith, C.O. Anderson & J.V. Watson

*MRC Clinical Oncology and Radiotherapeutics Unit, MRC Centre, Cambridge, UK*

The synthetic epipodophyllotoxin, VP-16-213, is an important new antitumour agent which can neither intercalate into DNA nor bind to DNA. However, the cytotoxic and cell cycle-kinetic effects of VP-16-213 are thought to relate to the induction of DNA damage, specifically protein-linked strand interruptions attributable to type II topoisomerase activity. This relationship has been explored in human cells derived from normal donors and A-T patients. The A-T derived cells (an SV40 transformed fibroblast line, a primary fibroblast strain and an EBV-transformed lymphoblastoid line) showed enhanced sensitivity (increased cell killing and elevated retention in G2 phase) following exposure to VP-16-213 in comparison with the responses of normal control cells. Further studies with transformed fibroblasts revealed that the intrinsic sensitivity (DNA strand breaks per lethal hit quantitated by nucleoid sedimentation) was the same in A-T and normal control cells, given that the A-T cell line accumulated more damage during short term drug exposure. Increased levels of DNA damage in A-T were not due to slow repair. The study suggests that abnormal topoisomerase II activity may be an important feature of A-T cells providing a molecular link between aberrant DNA function (transcription and rearrangement) and defective DNA repair.

### C.8 Initial rates of incision are different in normal, XP and XP heterozygote fibroblasts

S. Squires & R.T. Johnson

*CRC Mammalian Cell DNA Repair Group, Department of Zoology, University of Cambridge, Downing Street, Cambridge CBS 3EJ, UK*

We have used inhibitors of DNA repair synthesis to discriminate between XP cells from different complementation groups on the basis of their incision activity, and to distinguish between phenotypically normal XP heterozygotes, and wild type cells by means of a kinetic analysis of incision. By maximizing the frequency of DNA breaks measured, and by using a simple and sensitive alkaline lysis technique we have estimated the initial rates of incision after low levels of UV (0.25–10 $Jm^{-2}$) and over short intervals after irradiation. On this basis XP cells can be distinguished from one another by the abundance of enzyme(s) (Vmax) and by its affinity for the damaged site (Km). All XP cells tested express a low level of active enzyme but varied in their Km values. XPD and cells from the individual designated XP2LE express a high Km, while for XPH the value is very similar to wild type. Two XPA heterozygotes have wild type Km values but half the amount of active enzyme.

### C.9 Response of UVC (254 nm) radiation sensitive human mutants to radiation at defined UVA (334 nm, 365 nm) and visible (405 nm) wavelengths

R.M. Tyrrell

*Swiss Institute for Experimental Cancer Research, 1066 Epalinges, Switzerland*

Various cell lines derived from humans with sun-sensitive syndromes display slight (XP Variant, Bloom's) intermediate (XPC, XPD, Cockayne's) or high (XPA) sensitivity to UVC (254 nm) radiation. We have confirmed previous observations showing that XPA and XPD strains are slightly sensitive to radiation at UVA (320–400 nm) wavelengths and extended these studies with 3 independent XPA cell lines to show that the sector of effective repair involving the XPA gene product diminishes from 30 to 40% at 334 nm to ~20% at 405 nm. This may reflect an increasing fraction of lethal non-dimer DNA damage as a function of wavelength. However, there is also evidence that both UVA and visible wavelengths reduce excision repair capacity in human cells within the lethal fluence range. A third possibility, that damage to targets other than DNA becomes increasingly critical at longer wavelengths, is difficult to test experimentally. XPC, XP variant, Cockayne's and Bloom's cell lines show a sensitivity within the range of normal strains after treatment with UVA and visible radiations. This provides further evidence that XPA and XPD strains are deficient in a repair pathway distinct from that lacking in XPC strains.

### C.10 Sensitivity to sunlight in patients affected by trichothiodystrophy is related to the capacity to repair the UV-induced DNA damage

M. Stefanini[1], P. Lagomarsini[1], A. Fois[2], P. Balestri[2] & F. Nuzzo[1]

[1]*Istituto di Genetica Biochimica ed Evoluzionistica C.N.R., Via Abbiategrasso 207, 27100 Pavia, Italy;* [2]*Istituto di Clinica Pediatrica, Università di Siena, Italy*

Trichothiodystrophy (TTD) is a rare autosomal recessive disorder characterized by brittle hair with reduced sulfur content, mental and physical retardation, peculiar face, icthyosis; in some TTD patients a severe light sensitivity has also been described. We demonstrated a reduced capacity to repair UV induced damage in 4 patients affected by TTD with photosensitivity. The repair defect is due to the presence of xeroderma pigmentosum complementation group D (XP-D) mutation. This finding raises the question whether TTD is a clinical manifestation of XP-D mutation. We studied the ability to perform DNA repair in fibroblasts from a TTD patient with no photosensitivity and we found normal values of unscheduled DNA synthesis. This indicates that TTD without photosensitivity is independent from XP.

### D.1 SV40-based shuttle-vectors which can be encapsulated as virus particles

C.F.M. Menck, M.R. James & A. Sarasin

*Institut de Recherches Scientifiques sur le Cancer, BP No. 8, Villejuif 94802, France*

Shuttle-vectors have been widely used for gene transfer and gene expression in mammalian cells and also, more recently, for studying UV-induced mutagenesis in these cells. However, the introduction of these vectors is achieved by DNA transfection, which has certain disadvantages including inefficiency. Transfection of naked DNA may be one of the reasons for the high spontaneous mutagenesis seen with these plasmids. We therefore constructed a series of SV40-based shuttle-vectors that contain the entire SV40 late region and the intact replicon origin. The early genes, which encode small t- and large T-antigens, have been substituted by the bacterial miniplasmid πΔlac that has the minimal essential DNA sequences for replication and selection in *E. coli*, and the *lac0* sequence. These plasmids can replicate efficiently in monkey COS-7 cells, that produce constitutively the large T-antigen, and can form infectious virus. Plasmid DNAs from cells infected with these viruses have been rescued in bacteria and their stability analysed. DNA alterations, due to restrictions of the genome size which could be packaged as virus, are observed in one of the vectors, but stable vectors were also obtained. The 28 bp operator/repressor binding sequence (*lac0*) present in these vectors allows mutagenesis analysis by screening blue and white bacterial colonies on appropriate media. Initial data indicate that the plasmids are more stable after a first lytic cycle, and low spontaneous mutation frequences can be achieved.

(C.F.M. Menck has a post-doctoral dellowship from CNPq, Brazil, and M.R. James from ARC, France).

### D.2 Gene recombination in X-ray-sensitive CHO cells

A. Hamilton & J. Thacker

*MRC Radiobiology Unit, Chilton, Didcot, Oxford OX11 0RD, UK*

To assay recombination within mammalian cells we constructed pairs of vectors, derived from the pSV2*gpt* plasmid, with non-overlapping deletions in the *gpt* gene. These deleted vectors were transferred together into cells; recombination was seen as restoration of gene activity and confirmed by molecular analysis. The cells used were the CHO-K1 hamster line and the mutant sublines *xrs*1 and *xrs*7 isolated by Jeggo and Kemp (1983). These mutants are abnormally sensitive to ionising radiation and show some similarities to the human radiosensitive ataxia telangiectasia cells. Recombination efficiences, measured as the transformation frequency of the pair of deletion plasmids relative to that for the intact pSV2*gpt* plasmid under the same conditions, were similar for the CHO parent cells and for the *xrs* mutants. In both cell types these efficiencies were substantially enhanced by the introduction of a double-strand break into the homologous region of the deletion plasmids. However the linear increase in transformation frequencies found in CHO cells for both pSV2*gpt* and the deletion plasmid pairs with increasing DNA concentrations did not occur for the *xrs* mutants. Uptake of plasmid DNA was similar in all cell lines. Therefore we suggest that, although homologous recombination of plasmid molecules may take place normally in the *xrs* mutants, processes involved in the stable integration of plasmid DNA into genomic DNA are significantly impaired.

### D.3 Investigation of mutagenesis in transformed human fibroblasts using shuttle vectors

J.R. Lamb[1], M.R. James[2] & A.R. Lehmann[1]

[1]*MRC Cell Mutation Unit, University of Sussex, Falmer, Brighton, E. Sussex BN1 9RR, UK;*
[2]*CNRS, Institute de Recherche Scientifique sur le Cancer, BP8, 94802 Villejuif, Cedex, Paris, France*

We are developing a system to study mutagenesis in human cells at the base pair level. The approach we are taking is to passage Epstein–Barr Virus (EBV) based vectors through mammalian cells. Following mutagenic treatment of the cells, the vector is recovered and used to transform *E. coli* in which detailed molecular analysis can subsequently be carried out. The target sequence we have inserted into our plasmids is the first 120 base pairs of the bacterial *lacZ* gene. Mutations in this sequence are scored as white colonies against a blue background on X-gal plates in the bacterial host JM109. The cells we are using are the SV40-transformed human fibroblast lines: MRC5-V1 (normal), AT5BIVA (ataxia-telangiectasia), and SVOXPC (xeroderma pigmentosum). We have isolated several clones from each of these cell lines which maintain our EBV vectors episomally and we are in the process of establishing spontaneous mutation frequencies.

### D.4 Parvoviral DNA as a probe for the measurement of spontaneous depurination in mammalian cells

B. Avalosse, Y.Q. Chen & J. Rommelaere

*Department of Molecular Biology, Université Libre de Bruxelles, 1640 Rhode St Genèse, Belgium*

Apyrimidinic-apurinic (AP) sites are non-coding DNA lesions which appear spontaneously and are induced by various DNA-damaging agents. It is assumed that a fraction of these lesions can be tolerated during DNA replication in mammalian cells and are mutagenic. It therefore appears important to develop sensitive methods for the detection of AP sites in mammalian cells. The single-stranded DNA-containing parvovirus MVM was used to assess the spontaneous rate of AP site formation in mouse cells. A monomeric, double-stranded DNA species with covalently linked viral and complementary strands (turnaround RF-1) is generated during DNA replication and was tested for its AP site content. Purified turnaround RF-1 DNA was treated with an AP endonuclease and denatured with formamide. This treatment did not affect the integrity of non-depurinated DNA. In contrast, AP site-containing DNA was cleaved, generating incomplete molecules upon denaturation. The latter molecules were completed *in vitro* using the PolI Klenow fragment and $^{32}$P-labelled deoxyribonucleotide-triphosphates. The radioactivity of an internal restriction fragment allowed the quantification of AP sites in the viral genome. It was estimated that $4 \times 10^{-8}$ pmoles AP sites were accumulated per $5 \times 10^{3}$ bp viral RF DNA per 12 h. The method used reveals as little as one damaged parvoviral DNA molecule among 2,500 intact ones.

### D.5 Shuttle vectors in human cells

L. Daya-Grosjean, A. Sarasin & M. James

*Laboratory of Molecular Mutagenesis, Institut de Recherches Scientifiques sur le Cancer, Villejuif, France*

We are interested in the potential of shuttle vectors to facilitate the analysis, at the detailed molecular level, of DNA metabolism in human cells, particularly in the DNA repair deficient and cancer-prone genetic syndromes. In addition to the analysis of host-dependent mutagenesis, such a vector system may allow the facile introduction, expression and rescue of genetic loci of interest. In the first instance, we have inserted the replication origins or entire early regions of monkey and human papovaviruses into a small cosmid. These vectors replicate transiently in monkey COS-7 cells and in human cell lines transformed with an ori-defective SV40 recombinant which provide *T*-antigen *in trans*. The inclusion in these vectors of the hybrid neomycin resistance gene allows for selection in mammalian cells using the drug G418. Selection of XP4PA-SVwt cells transfected with these vectors results in rapid establishment of cell clones which show episomally-maintained plasmid, a phenomenon which appears much more stable in the human cells than in monkey COS cells. Recently, we have constructed EBV-based shuttle vectors containing *lacZ* and have obtained, after transfection and selection, human fibroblast cell lines which contain the vectors as episomal plasmids at between 100–1,000 copies per cell. Further derivatives of the above SV- and EBV-vectors have been constructed which contain the CAT gene controlled by various promoters. Our experiments provide quantitative data on the stability of the vectors (scoring *lacZ* after plasmid rescue), copy number of the episomal vectors and efficiency of expression of inserted genes. These data allow precise comparisons between different vector/host systems, after transient or long-term maintenance of the vectors and between different eukaryote promoters.

### D.6 Mutagenesis in Herpes virus grown in serum stimulated or in serum free and UV-irradiated human cells

B. Lopez, S. Nocentini & J. Coppey

*Institut Curie—Biologie, Paris, France*

In order to analyse mutagenesis in HSV progeny (iododeoxycytidine resistance) from virus grown in human cells performing normal DNA synthesis or DNA synthesis triggered by UV damage, host cells were kept in serum free medium and either UV exposed (10 J m$^{-2}$, no serum until the end) or refed by 15% FCS at 0, 6, ..., 48 h before infection with intact or UV-HSV. DNA synthesis was analysed in parallel by [$^{3}$H]-thymidine incorporation and autoradiography. It mainly appears that the rate of mutation is similar for infecting intact HSV but 10 times lower for infecting UV-HSV in cultures refed by serum at time 0 than in serum free cultures. The rate is significantly lowered in cultures infected at time of UV exposure then slightly increases following UV and serum free maintenance with comparable patterns for infecting intact or UV-viruses. Autoradiographies show, following a phase of DNA repair in all cells, the occurrence of normal DNA synthesis (heavy labelling) in a fraction of the cells.

### E.1 A DNA repair domain

V.A. Bohr, D.S. Okumoto & P.C. Hanawalt

*Department of Biological Sciences, Stanford University, Stanford 94305, California, USA*

We have measured the frequency of pyrimidine dimers after $20\,J\,m^{-2}$ UV light in restriction fragments in and around the dihydrofolate reductase (DHFR) gene in CHO cells, amplified for the gene. The technique used is briefly described in our abstract "Heterogeneity in mammalian cells". Repair was measured after 8 and 24 h. We found maximal repair efficiency (more than 40% after 8 h) at the 5′ end of the gene and in its 5′ flanking sequences. The repair efficiency declined in both the 5′ and 3′ directions from there. We found less repair efficiency in the 3′ half of the gene, (~20% after 8 h) than in its 5′ end, and repair further declined in the 3′ flanking sequences where it was less than 10% after 8 h. We also analyzed the regions in and around the DHFR gene for the level of methylation using isoschizomeric enzyme analysis. The only sites of hypomethylation were at the 5′ end of the gene, where also the repair efficiency was maximal. It is possible that the level of methylation plays an important role in determining chromatin accessibility for repair enzymes. The repair domain is about 50–80 kb in size, and this correlates well with proposed sizes for genomic, chromatin structural domains. Our DNA repair assay may be a probe for chromatin structure.

### E.2 DNA repair heterogeneity in mammalian cells

V.A. Bohr, D.S. Okumoto, H. Madhani, C.A. Smith & P.C. Hanawalt

*Department of Biological Sciences, Stanford University, Stanford 94305, California, USA*

We have studied the repair of pyrimidine dimers damage in defined DNA sequences in mammalian cells. Restricted genomic DNA from UV-irradiated cells is treated or not with the dimer-specific T4 endonuclease V, electrophoresed under denaturing conditions, Southern transferred and probed for the specific restriction fragments of interest. The proportion of fragments free of endonuclease sensitive sites in each sample is determined from the difference in the amount of probe hybridized at the position of full length fragments, for T4 endonuclease-treated and untreated samples. The overall frequency of endonuclease sensitive sites per restriction fragment is then derived using the Poisson expression.

In CHO cells in which the dihydrofolate reductase (DHFR) gene is amplified, roughly 70% of the dimers were removed from the gene in 26 h while 15% were removed from an upstream sequence or from the genome overall. Preferential repair of vital "housekeeping" genes such as DHFR may account for the high UV resistance of CHO cells in spite of low overall repair levels (Bohr *et al.*, 1985, *Cell*, **40**, 359). Similar results have been obtained in CHO cells in which the DHFR gene is not amplified.

In human fibroblasts and epidermal keratinocytes repair in the DHFR gene was ~70% in 24 h. However, in XPC• cells exhibiting UV sensitivity and only 10–20% repair of dimers in the genome overall, repair was markedly deficient in the DHFR gene. A comparison between overall genome repair, repair in the DHFR gene and cellular survival in CHO, XPC and normal human cells indicates that UV survival is better correlated with repair of the essential DHFR gene than with overall genome DNA repair. We have analyzed repair after UV damage in proto-oncogenes in mouse 3T3 cells. The constitutively actively transcribed c-*abl* proto-oncogene is proficiently repaired while the largely nontranscribed c-*mos* oncogene is not. We conclude that the repairability of damage depends upon its location in the genome and the functional state of the DNA at that site. (Work supported by grants from the National Institutes of Health and the American Cancer Society).

### E.3 The kinetics of repair

J.M. Boyce, S.J. MacCready & B.S. Cox

*Department of Plant Sciences, South Parks Road, Oxford OX1 3RA, UK*

In repair-proficient yeast strains repair of UV damage can be measured as loss of UV-endonuclease sites. The loss of sites appears to occur in two phases, an initial fast reaction and a later slow reaction. Several models could explain these kinetics:

(i) the rate of repair is proportional to the number of dimers remaining in the DNA;
(ii) dimers in some regions of the DNA, or in DNA with particular characteristics, are preferentially repaired;
(iii) cells in different stages of the cell cycle show different levels of repair efficiency;

(iv) there are two pathways of repair with different rates of action.

Evidence for and against these models will be presented.

### E.4 Inhibition of repair of X-ray-induced DNA damage by hyperthermia

H.H. Kampinga & A.W.T. Konings

*Department of Radiopathology, Bloemsingel 1, 9713 BZ Groningen, The Netherlands*

Mild heating of cells prior to X-irradiation results in an enhanced radiosensitivity. It is assumed that the observed hyperthermic inhibition of repair of radiation-induced DNA damage is the main cause for this heat radiosensitization. We found, e.g. that heating cells reduced the rate of repair of X-ray-induced alkali labile sites (as determined by the hydroxylapatite chromatography method). The reasons for this repair inhibition might possibly be a heat inactivation of enzymes involved in the repair processes and/or altered chromatin structure.

We found a hyperthermic inactivation of DNA polymerase $\alpha$ and $\beta$ at a rate which seemed to be related to heat radiosensitization. It was decided to investigate the role of these enzymes in the process of heat-induced inhibition of DNA repair by using specific enzyme inhibitors.

Aphidicolin, an inhibitor of DNA polymerase $\alpha$, could substantially reduce the rate of repair of X-ray-induced alkali labile sites (including DNA breaks). However, when combined with heat the extra inhibitory effect of aphidicolin varied from less than additive to no extra effect at all at higher heat doses.

We like to conclude that, although DNA polymerase $\alpha$ seems to be involved in repair of X-ray-induced DNA damage and although this enzyme is partially inactivated by heat, this heat inactivation of DNA polymerase $\alpha$ cannot (solely) be responsible for the observed hyperthermic inhibition of repair of X-ray-induced DNA damage.

### E.5 Deoxycytidylate deaminase activity in *E. coli*: inducibility by irradiation as part of the SOS response

A.-M. Estevevon & N. Sicard

*Centre de Recherche de Biochimie et Génétique Cellulaires du CNRS, 118 route de Narbonne 31062 Toulouse, France*

A deoxycytidylate (dCMP) deaminase activity in *E. coli* which is not detectable in normal cells is induced by UV or gamma irradiation. This inducibility is affected by a *rec*A13 mutation. In a *rec*A44I (tif) mutant, the enzyme activity is induced at non-permissive temperature. This suggests its involvement in the SOS response.

### E.6 Repair of UVC induced lethal damage to human fibroblasts is faster in non-dividing cells

S.M. Keyse & R.M. Tyrrell

*Swiss Institute for Experimental Cancer Research, 1066 Epalinges, Switzerland*

Exposure of UVC damaged human fibroblasts to aphidicolin under the appropriate conditions irreversibly blocks excision repair of potentially lethal damage (PLD). By delaying the addition of aphidicolin for varying times following irradiation, the kinetics of biologically effective excision repair have been followed in confluent (density inhibited), arrested (low serum) and exponentially growing human skin fibroblasts using cell survival as the end point. Both confluent and arrested fibroblasts exhibit overall repair kinetics which are the product of two distinct first order rates. The rate constant of the initial rapid component of repair is $\sim 0.31\,h^{-1}$ while that of the second slower component is $\sim 0.064\,h^{-1}$. These parameters were found to be independent of UV fluence over the range $1.5$–$6.0\,Jm^{-2}$ using cells from both types of non-dividing culture. In contrast, the rate of repair of PLD in fibroblasts irradiated in exponential growth was found to be fluence dependent. At the lowest UV fluence ($1.5\,Jm^{-2}$) the initial (fast) rate of repair is close to that found in arrested cells ($0.42\,h^{-1}$) while at higher fluences this rate declined in a fluence dependent fashion being $0.2\,h^{-1}$ after a fluence of $3.0\,Jm^{-2}$ and $0.063\,h^{-1}$ after a fluence of $6.0\,Jm^{-2}$.

### E.7 The role of intracellular proteinases in repair of potentially lethal damage

M. Korbelik[1], A. Suhar[2], J. Škrk[2], V. Turk[3] & D. Petrović[1]

[1]*R. Bošković Institute, Zagreb;* [2]*J. Stefan Institute, Ljubljana, Yugoslavia;* [3]*Institute of Oncology, Ljubljana, Yugoslavia*

In this work it is shown that addition of proteinases or proteinase inhibitors to Chinese hamster V79

cells in plateau-phase of growth immediately after irradiation modifies activity of potentially lethal damage repair (PLDR). The stimulatory effect was seen with calf liver neutral proteinase, and to a lesser extent, with inhibitor pepstatin A. Other proteinases, which belong to neutral, cysteine and aspartic superfamilies, as well as proteinase inhibitors examined, acted inhibitory on PLDR to different degree. Alpha-chymotrypsin and inhibitors of chymotrypsin completely inhibited PLDR. These data indicate that a neutral serine proteinase with chymotrypsin-like properties may be directly involved in PLDR process. Because of comprehensive intercorrelation of different intracellular proteinases activities, other proteinases and proteinase inhibitors can affect PLDR process in a less direct way. Moreover, by influencing cellular proliferative activity, these agents can also affect PLDR process.

### E.8 *In vitro* replication of UV-irradiated DNA with DNA polymerase III holoenzyme of *E. coli*

Z. Livneh

*Department of Biochemistry, The Weizmann Institute of Science, Rehovot 76100, Israel*

Investigation of the *in vitro* replication of UV-irradiated single stranded DNA with *E. coli* DNA polymerase III holoenzyme (Pol III HE) in the presence of single-stranded DNA binding protein (SSB) yielded the following results:

1. Under normal *in vitro* conditions, in the presence of SSB, DNA polymerase III holoenzyme can bypass pyrimidine-photodimers to a significant extent (at least 30% bypass) even in the absence of SOS induced proteins.
2. SSB is required for efficient bypass of pyrimidine-photodimers.
3. Inhibition of the 3′→5′ proofreading exonucleolytic activity does not increase bypass of pyrimidine-dimers.
4. RecA protein does not increase bypass.
5. Pol III HE seems to be unable to elongate a DNA chain terminated at a putative pyrimidine-dimer.
6. Termination involves dissociation of Pol III HE, which can then re-initiate synthesis at available primer-templates.

Based on these observations a model for SOS mutagenesis is proposed.

### E.9 Induction and repair of UV-induced lesions in DNA of human skin cells as detected by immunochemical methods

S. MacFarlane, S.L. Roza, G.P. van der Schans & P.H.M. Lohman

*Medical Biological Laboratory TNO, P.O. Box 45, 2280 AA, Rijswijk, The Netherlands*

Immunochemical methods have been applied with considerable success to detect DNA modifications resulting from exposures to genotoxic chemicals or radiations. We have obtained a serum highly specific for UV-irradiated DNA (UV-DNA) and this is being applied to studies of the induction and repair of DNA damage after exposure of cells to UV-B irradiations. Characterization of the serum in a competitive ELISA revealed a dose–effect relationship of increasing inhibition with increasing doses of UV-B to the competitor DNA in the range of 0–10 $kJ\,m^{-2}$. Doses as low as 0.5 $kJ\,m^{-2}$ are detectable in the DNA isolated from cells irradiated in culture; such a dose corresponds to ca. 85% survival in normal human skin cells. We monitored induction and repair of UV-lesions in the DNA of cultured human fibroblasts using both the immunochemical and an *M. luteus* UV-endonuclease detection assay. The lower limits of detection for both systems were comparable, but a major advantage of the immunochemical detection system is that the DNA need not be labelled with radioactive tracers and, thus, it offers the means to study UV-damage induction and repair *in vivo*. We are currently extending our investigations to *in vivo* studies with some success. Further, by making use of enzymatic and/or UV-light photoreversal, we can apply our antibody assay to the detection of non-dimer lesions induced by UV in DNA, with particular emphasis on the detection of 6,4′-[pyrimidine-2′-one]-pyrimidine photoproducts.

### E.10 AP sites in alkylated and $\gamma$-irradiated mammalian cells

M.F. Moran & K. Ebisuzaki

*Cancer Research Laboratory, University of Ontario, London, Ontario, Canada*

A highly sensitive method of estimating apurinic/apyrimidinic (AP) sites in DNA has been developed by coupling endonuclease IV (endo IV) with Kohn's alkaline elution technique. Endo IV treatment had no effect on the rate of eulution of DNA

from untreated or γ-irradiated (held in ice) HeLa S3 cells. However, alkylation of cells with dimethyl sulfate (DMS) followed by further incubation at 37°C showed an initial appearance of AP sites and strand breaks followed by a progressive repair of both lesions. When γ-irradiated cells were allowed a post-damage recovery period at 37°C, a similar sequence of events occurred. These results suggest that the repair of DNA damaged by alkylation or γ-irradiation proceeds in part via an AP intermediate and a DNA glycosylase→AP endonuclease-initiated pathway is indicated.

### E.11 **Rapid repair of the DHFR gene in human cells**

I. Mellon, V. Bohr, C.A. Smith & P.C. Hanawalt

*Department of Biological Sciences, Stanford University, Stanford, CA 94305, USA*

Removal of pyrimidine dimers from different DNA sequences in human cells in which the dihydrofolate reductase gene is amplified was measured using a dimer specific endonuclease. For analysis of the DHFR gene region we measured the fraction of restriction fragments free of nuclease sensitive sites by Southern blotting alkaline agarose gels. Within 4 h after 5 or 10 J m$^{-2}$ 254 nm UV more than 60% of the dimers were removed from a 20 kb fragment which lies entirely within the transcription unit of the DHFR gene and from a 25 kb fragment located in its 5′ flanking region. For the overall genome we determined frequencies of nuclease sensitive sites from molecular weights of unrestricted DNA using alkaline sucrose gradients. As expected, the majority of the dimers were removed after 24 h, but only 25% were removed after 4 h. Thus repair in a 50 kg region that includes the transcriptionally active DHFR gene appears to be significantly faster than that in the total cellular DNA. By probing DNA from alkaline sucrose gradients to determine the molecular weights of DNA containing specific sequences, we confirmed the rapid repair of the DHFR region and showed that repair in the non-transcribed repetitive alpha sequence resembled that of the genome overall. Preliminary results indicate that preferential repair is also observed in cells in which the gene is not amplified.

The preferential repair that has been observed in active sequences in rodent cells in culture might relate to a selective loss in repair capacity for silent DNA, resulting in a low capacity for removal of pyrimidine dimers from the genome overall. These results with human cells indicate preferential repair also occurs in repair proficient cells.

### E.12 **The effect of radioprotector WR1065 on radiation induced cell killing, mutagenesis, DNA damage and repair in V79 cells**

B. Nagy[2] & D.J. Grdina[1]

[1]*Division of Biological Medical Research, Argonne National Laboratory, Illinois 60439, USA: and* [2]*Laboratory of Experimental Cancerology, Central Institute for Tumors, Zagreb, Yugoslavia*

WR 1065, 2-((aminopropyl)amino) ethanethiol, protects against radiation-induced cell killing and mutagenesis at the hypoxanthine-guanine phosphoribosyl transferase (HGPRT) locus in V79 cells. Formation of single-strand breaks (SSB) in DNA is reduced by the presence of WR1065 during irradiation, but subsequent postradiation rejoining processes are inhibited by this agent. WR1065 effectively protected against cell death only if present during irradiation, but reduced radiation-induced mutations regardless of when it was administered. Damage and repair of SSB in DNA following a dose of 10 Gy was measured by using alkaline elution. When WR1065 was added immediately following irradiation and repair was monitored, the protector inhibited the rate of rejoining of SSB by about a factor of 3. It may be that this reduction in the rate of SSB rejoining is accompanied in some manner by a greater fidelity of repair leading to reduction in mutagenesis. (Supported by U.S. DOE contract no. W-31-109-ENG-38 and NIH/NCI grant no. CA-37435).

### E.13 **Interrelationship of deoxynucleoside triphosphate pools, the regulation of DNA synthesis and the induction of mutations by UV or ionizing radiation**

C.N. Newman & J.H. Miller

*Pacific Northwest Laboratory, Richland, Washington 99352, USA*

Chinese hamster ovary cells were grown in Ham's F-12 medium with 10% foetal calf serum (medium A) or in medium A with 2 mM CdR (medium B). No discernable differences in culture doubling times (14.5 h), or cell cytology were observed. However, exposure to UV or ionizing radiations always yields a 2- to 20-fold lower mutation frequency in medium B than in medium A. Studies of DNA precursor metabolism suggest that in medium B cells depend upon salvage pathways for the production of deoxypyrimidine triphosphates (dPTPs) while in medium A, dCTP is synthesized *de novo* and serves as the source of cellular dTTP. Since we have shown that these dPTPs may serve to regulate DNA synthesis activity, alkaline sucrose gradients were employed to examine and compare replication in the two media. Our results with UV radiation show there is a preferential inhibition of initiation of replicon synthesis in medium B, in comparison to medium A. From this we propose that fewer lesions are found in partially replicated DNA in medium B and repair of premutagenic damage in lesions serving as transient blocks to replication (which occurs more frequently in medium A) is error prone. (Work supported by U.S. Department of Energy Contract DE-AC06-76RLO 1830).

### E.14 The effects of low repeated doses of filtered near-UV light on Chinese hamster cells

M. Osmak[1], C.K. Hill[2], M. Ikebuchi[2], M.M. Elkind[3] & A. Han[3]

[1]*Department of Experimental Biology and Medicine, Ruder Bošković Institute, 41000 Zagreb, Yugoslavia;*
[2]*Department of Experimental Radiotherapy, USC Medical School, Los Angeles, CA 90015, USA;*
[3]*Department of Radiology and Radiation Biology, Colorado State University, Fort Collins, CO 80523, USA*

The response of Chinese hamster V79 cells to repeated low doses of filtered near-UV light was examined. Cell survival and the induction of mutation at the hypoxanthine-guanine phosphoribosyl transferase locus using resistance to 6-thioguanine (6-TG) and the induction of mutation at the sodium/potassium ATPase locus using ouabain (OUA) were end points of our study. With increasing accumulated dose of filtered near-UV light an increase in resistance to cell killing was observed accompanied by a gradual decrease in induction of mutants resistant to 6-TG and OUA. The increased resistance to cell killing and to mutation induction indicates that during exposure to low repeated doses of filtered near-UV light the cells become adapted to filtered near-UV light.

### E.15 DNA excision repair in cultured fibroblasts of affected and unaffected psoriatic skin

G. Partsch[1], J. Neumüller[1], F. Mayer[2] & R. Eberl[1]

[1]*Ludwig Boltzmann—Institute of Rheumatology and Balneology, Vienna–Oberlaa, Austria;*
[2]*Dermatological Department, Municipal Hospital Vienna–Lainz, Austria*

Skin punch biopsies from affected and unaffected areas of 3 patients suffering from different degrees of psoriasis were cultured and used for DNA excision repair experiments. The [$^3$H]-thymidine incorporation after depression of semi-conservative DNA synthesis by 2 mM hydroxyurea was compared to the [$^3$H]-thymidine incorporation after additional UV irradiation (20 J m$^{-2}$) and taken as a measure for DNA repair activity. Irradiation alone was of different effect on unaffected and affected skin fibroblasts with the cells of the normal skin ($n = 2$) being more sensitive.

A more active DNA excision repair was confirmed in 2 out of 3 fibroblast cultures from lesions as compared to the healthy skin of the same patient. One of these cultures which was studied in an early (8/9th) as well as in an older passage (24th) showed that these particular properties of affected and non-affected skin fibroblasts subsist during cultivation. It is therefore supposed that these effects are genetically stable.

### E.16 The increased recA protease activity in UV-irradiated *E. coli* in the presence of rifampicin

E. Salaj-Šmic and Ž. Trgovčević

*Ruder Bošković Institute, 41001 Zagreb, Yugoslavia*

In UV-irradiated *E. coli* bacteria, synthesis of the RecA protein is greatly reduced by a low concentration of rifampicin (4 $\mu$g ml$^{-1}$). In contrast to this, however, the activation of RecA protein to function as a protease is increased in the presence of a low concentration of rifampicin, as shown by our biological assay (Salaj-Šmic *et al.*, *Nucl. Acid Res.*, **13**, 1563, 1984). The increased protease activity of RecA was also confirmed with *E. coli* bacteria lysogenic for $\lambda ind^s$. In this case, the killing effect of the induced lambda was more pronounced in the presence of rifampicin than in its absence.

### E.17 **Immunochemical methods for the detection of DNA lesions in human cells**

G.P. Van der Schans, L. Roza, S. MacFarlane & P.H.M. Lohman

*Medical Biological Laboratory TNO, P.O. Box 45, 2280 AA, Rijswijk, The Netherlands*

Biological dosimetry of radiation exposure might be based on quantitative detection of lesions induced in chromosomal DNA, preferentially in nucleated cells of the peripheral blood. Advanced immunochemical methods appear very promising in this respect. Lesions used in this approach should be sufficiently persistent. Lesions in study, induced by ionizing radiation and detectable by immunochemical methods, are: thymineglycols (the most abundant base damage), pyrimidine dimers, single-strandedness and, finally, adducts of DNA with amino acids.

Amounts as small as $10^{-15}$ mol per determination are detectable. The lower limit depends on a number of variables, such as the amount of lesions per unit dose and suppression of background by means of pre-separation. Conceivably, 1 Gy may become detectable. The technique is in the early stages of development and not yet applicable as a biological indicator for radiation exposure. When fully developed, it will not be limited to the detection in isolated DNA, but also be applicable on the single-cell level, which will enable us to study small biopsies and to distinguish between total and partial body irradiation.

Simultaneously, an alternative method is pursued based on the occurrence of repair processes in the cell acting on radiation damage. Primarily being meant for calibration purposes, it may be suitable for practical application under certain circumstances.

### E.18 **Effect of physico-chemical modifiers of energy metabolism on DNA repair**

A. Sharma

*Department of Biophysics, All India Institute of Medical Sciences, New Delhi, India*

Energetics of DNA repair via excision repair pathway was investigated in peripheral blood leucocytes obtained from normal and chronic myeloid leukemic (CML) subjects. UV (254-nm) light-induced DNA repair was modulated by different temperatures and inhibitors of energy metabolism (glucose analogues and antimycin-A). DNA repair (UDS) was measured by unscheduled DNA synthesis technique, using liquid scintillation counting and autoradiography. Relevant parameters of energy metabolism were measured under identical experimental conditions. Following results were obtained: 1. UDS was differentially inhibited in normal and CML blood leucocytes by the combination of 2-deoxy-D-glucose and antimycin-A. 2. In CML leucocytes: (*a*) In absence of respiration, inhibition in DNA repair by glucose analogues was found to bear a linear correlation with inhibition in rate of glycolysis. (*b*) A minimum threshold-rate of glycolysis was found to be necessary for DNA repair under these conditions. (*c*) UDS was observed to increase with a rise in temperature up to 40°C and fall thereafter. Decrease in UDS was enhanced with longer periods of heat treatment. (*d*) Presence of metabolic inhibitors does not significantly alter activation energy of DNA repair.

### E.19 **The effect of inhibitors of DNA polymerase α on the size of the excision repair patch**

I.G. Walker, J.P.H. Th'ng & D. Lee

*Department of Biochemistry, University of Western Ontario, London, Ontario, Canada*

During excision repair of UV light- or dimethyl sulfate (DMS)-induced damage to DNA the patch size for actively replicating KB or T98G cells is around 20 nucleotides. When confluent T98G cells or "quiescent" KB cells are used the patch size is around 10 nucleotides. This value can be increased to around 20 nucleotides in T98G cells if a large excess of BrdUrd is included in the repair incubation medium. With "quiescent" KB cells the patch size is not increased by excess BrdUrd. For all of these experimental conditions, when excision repair of UV- or DMS-damage takes place in the presence of aphidicolin, the patch size is found to be several times that found in its absence. Cytosine arabinoside (ara-c) was also found to increase the repair patch size in contact inhibited T98G cells following treatment with DMS. Given the inhibitory specificity of aphidicolin and ara-c for DNA polymerase α these results provide additional evidence that DNA polymerase α plays a role in the excision repair of DNA damaged by UV light or DMS. It is postulated that the inhibitors interrupt the processivity of the DNA polymerase α holoenzyme and this allows an exonuclease to enlarge the repair site.

### F.1 Genetic evidence for excision repair of $O^6$-alkyl guanine

J.M. Boyle, M. Fox, R. Saffhill & G.P. Margison

*Paterson Laboratories, Christie Hospital, Manchester M20 9BX, UK*

Human and Chinese hamster cells which lack $O^6$-alkyl DNA alkyltransferase (AT) activity in cell extracts are able to remove $O^6$-MedG and $O^6$-nBudG determined by RIA of enzyme digests of DNA from cells exposed to MNU or BNU. Fibroblasts from xeroderma pigmentosum (XP) complementation groups A and G which show <5% unscheduled DNA synthesis following exposure to UVC, failed to remove $O^6$-nBudG. Hence it appears that $O^6$-alkyl guanine is repaired in cells which lack AT by a process which is defective in XP cells, presumably nucleotide excision repair. In normal human cells excision repair accounts for most of the $O^6$-nBudG removed, with only a small amount being removed by AT. In Chinese hamster cell lines, neither V79 nor V79/79 cells have AT activity. Both cell lines are unable to remove $O^6$-nBudG, but only V79/79 is able to remove $O^6$-MedG, suggesting that substrate specificity is, in part, defined by recognition of some topographical feature of the alkyl residue chain length.

### F.2 Plasminogen activator associated with unrepaired DNA damage

B. Brdar

*Central Institute for Tumors, Zagreb, Yugoslavia*

Alkylating agents, mechlorethamine and N-methyl-N-nitro-N-nitrosoguanidine, induce the production of plasminogen activator (PA) in U-87MG cells, an alkylation DNA repair deficient ($Mer^-$) human glioblastoma strain. Enzyme induction was not observed, however, in U-178MG and SH-101 cells, alkylation repair proficient ($Mer^+$) glioblastoma strain, or in HeLa cells, which reactivated and supported well the growth of alkylation damaged adenovirus 3. In alkylation repair defective U-87MG strain, enhanced production of PA occurred in narrow concentration range of treatment with either alkylation agent, causing a 20–50% inhibition of [$^3$H]-thymidine incorporation. Maximum PA induction was observed between 32 to 48 h after alkylation treatment and the levels of enzyme produced were 5 to 10 times those of untreated control levels. This alkylation dependent enzyme induction required protein synthesis for it did not occur in the presence of cycloheximide. It was hence concluded that PA induction in alkylation repair deficient human cells is caused by unrepaired DNA damage and that it may represent a eukaryotic SOS-like function. In addition, PA induction may be useful as a sensitive assay for the identification of alkylation repair defective human tumours.

### F.3 The toxic effects of alkylating agents are reduced in mammalian cells expressing a truncated *E. coli* gene coding for $O^6$-alkylguanine alkyltransferase

J. Brennand & G.P. Margison

*Paterson Laboratories, Christie Hospital, Manchester M20 9BX, UK*

In order to examine the role of $O^6$-alkylation of guanine in DNA in the toxic, mutagenic and sister chromatid exchange inducing effects of alkylating agents, a section of the $O^6$-alkylguanine ($O^6$-AG)-alkylphosphotriester (AP) dual alkyltransferase (AT) gene which codes only for the $O^6$-AG activity has been isolated and ligated into a retrovirus-based antibiotic-selectable expression vector. The recombinant plasmid has been transfected into AT-deficient Chinese hamster V79 RJKO cells and an antibiotic-resistant clone (SB) expressing AT activity has been isolated. By comparison with extracts of *E. coli* harbouring plasmids coding for either the dual AT or only $O^6$-AG AT in an *in vitro* assay under substrate-limiting conditions, it was shown that SB cells expressed only $O^6$AG AT and this was at a level of $\sim 200\,fmol\,mg^{-1}$ protein. The *E. coli* truncated gene product was shown to act on $O^6$-methylguanine in host cell DNA and in comparison with control cells, to reduce the toxic effects of alkylating agents that react extensively with oxygen atoms in DNA.

### F.4 Selection of nitrogen mustard resistance in a rat tumour cell line results in loss of guanine $O^6$-alkyl transferase activity

S.W. Dean, N.W. Gibson & K.D. Tew

*Division of Medical Oncology, Lombardi Cancer Center, Georgetown University, Washington, DC 20007, USA*

Cell killing, sister chromatid exchange (SCE),

DNA-interstrand crosslinks and DNA-protein crosslinks were assayed in nitrogen mustard-resistant Walker 256 carcinoma (WR) cells and the parent cell line (WS) after treatment with 5-[3-(2-chloroethyl) triazen-1-yl]imidazole-4-carboxamide (MCTIC). The WR cells, which also express collateral sensitivity to chloroethyl nitrosoureas, (CENUs) were approximately twice as sensitive to the cytotoxic effects of MCTIC as were WS. There was no difference between the two cell lines in the frequency of MCTIC-induced SCEs. Following treatment with 100 $\mu$M MCTIC, there was a rapid accumulation of both DNA-interstrand and DNA-protein crosslinks in the WR cell line, which reached a maximum at 6 and 12 h respectively. There was considerably less crosslinking in the WS cells and both cell lines were proficient in repairing most of the crosslinks by 24 h. Measurement of guanine $O^6$-alkyl transferase ($GO^6AT$) activity showed the enzyme to be present in WS but not in WR cells. These data indicate that the collateral sensitivity of NM-resistant WR cells to chloroethylating drugs is due to loss of $GO^6AT$ activity which is present in the parent line.

### F.5 DNA ethylations induced by ethylnitrosourea in the wild type, *cdc4* and *cdc7* strains of *Saccharomyces cerevisiae.*

J.C. Fox, S. Edwards & R. Waters

*School of Biology, University College, Swansea, UK*

In a dividing culture cells at the G1 phase are obviously a heterogeneous population at various steps within this phase. Temperature sensitive *cdc* mutants have enabled the G1 phase to be divided into three steps as defined by the mutants *cdc* 28, *cdc* 4 and *cdc* 7. The earliest is governed by *cdc* 28 and its completion results in spindle pole body duplication. The next is controlled by *cdc* 4 and involves spindle pole body separation and the formation of a G1 folded chromosome. Both these events occur in *cdc* 7 mutants, and *CDC* 7 is involved in the last step that governs the initiation of DNA synthesis. Amounts of radioactivity associated with the marker peaks for O-6 ethylguanine, N-7 ethylguanine and 3-ethyladenine after the exposure of cells to 2, 4 or 6 mM [$^3$H] ENU, DNA purification and HPLC analysis indicate that there is no significant difference between wild type, *cdc* 4 and *cdc* 7 as regards the induction of ethylations at the N-7 of guanine, the O-6 of guanine or at the N-3 of adenine. Second, the increase with dose of ethylations at the O-6 position appears to be less than that at the N-7 position. The amounts of radioactivity associated with 3-ethyladenine were relatively low. It is therefore difficult to comment on the slope with increased dose for this lesion. Therefore the changes in chromosome folding associated with the progression from *cdc* 4 to the *cdc* 7 mediated step do not modify the ability of this agent to ethylate the DNA.

### F.6 The toxicity of MTIC in cultured human cells of different Mer/Rem phenotypes

J.M. Lunn[1], A.L. Harris[1], P.M. Brown[1], C. Pierpoint[2] & B.T. Golding[2]

[1]*Cancer Research Unit and* [2]*Department of Organic Chemistry, University of Newcastle Upon Tyne, Newcastle Upon Tyne NE1 4LP, UK*

Toxicity of MTIC, the active metabolite of the clinically used drug dimethyl-triazeno-imidazole-carboxamide (DTIC), was assessed by measuring proliferation of cultured cells following exposure to MTIC. Sensitivity increased in the order HT29 ($Mer^+Rem^+$) < A549 ($Mer^+Rem^-$) < VA13 ($Mer^-Rem^-$), indicating the importance of the $O^6$-methylguanine lesion. Further, after treating DNA *in vitro* with MTIC, $0^6$-methylguanine could be detected by HPLC.

MTIC cytotoxicity could be potentiated by the inclusion in the culture medium of 3-acetamidobenzamide (3AAB), an inhibitor of adenosinediphosphoribosyl transferase (ADPRT). However, in the presence of 3AAB neither HT29 nor A549 cells were rendered as sensitive to MTIC as VA13 cells.

It would appear that more than one mode of cytotoxicity is involved in the action of MTIC.

### F.7 Enhancement of $O^6$-methylguanine-DNA-methyltransferase in mammalian cells after various treatments

P. Lefebvre & F. Laval

*Groupe "Radiochimie de l'ADN", Institut Gustave Roussy, 94805 Villejuif, France*

We have previously shown that a rat hepatoma cell line ($H_4$ cells) could be adapted by pretreatment with N-methyl-N′-nitro-N-nitrosoguanidine to the toxic and mutagenic effects of this compound (*Proc. Natl Acad. Sci. USA* **81,** 1062, 1985) and that the O$^6$-MeGua transferase activity was increased about 3-fold in adapted cells (*Biochimie,* **67,** 361, 1985).

The present experiments were designed in order to know whether other cell treatments could modify the number of O$^6$-MeGua transferase molecules. $H_4$ cells were treated with various agents known to induce different types of DNA damage: $\gamma$ or UV-irradiation, heat treatment, incubation with different compounds (mitomycin, *cis*-dichlorodiammine platinum II, 2-methyl-9-hydroxyellipticinium, bleomycin ...). The assay measured the removal of O$^6$-methylguanine from [$^3$H]-alkylated DNA by cellular extracts. The results show that 48 h after the various treatments, the O$^6$-MeGua transferase activity is increased by 2- to 6-fold. This increase is due to *de novo* protein synthesis and is not related to cell cycle modifications.

The increase of the O$^6$-MeGua transferase activity represents an actual increase of the active molecules in the cells as the mutation frequency is lower in cells treated with N-methyl-N′-nitro-N-nitrosoguanidine 48 h after a pretreatment than in non-pretreated cells.

### F.8 **Removal of the promutagenic lesion O$^6$-methylguanine from mitochondrial DNA**

K.A. Myers, R. Saffhill & P.J. O'Connor

*Paterson Laboratories, Christie Hospital, Manchester M20 9BX, UK*

We have used radioimmunoassay to measure the preferential formation of O$^6$-methylguanine (O$^6$-MeG) in rat hepatic mitochondrial DNA (mtDNA) compared to nuclear DNA (nDNA). Over a three day period O$^6$-MeG is lost from mtDNA with similar kinetics to that for nDNA, suggesting that repair in mitochondria may occur by a similar mechanism. When animals are given a single dose of 2-acetylaminofluorene to enhance the repair of O$^6$-MeG in nDNA a similar increased rate of removal from mtDNA is also observed. Extracts of mitochondria contain assayable levels of O$^6$-MeG methyltransferase (MT) activity and when the mitochondria are treated with digitonin to remove the outer membrane, O$^6$-MeG MT levels are largely unaffected while the marker, acid phosphatase activity is reduced 12- to 13-fold. When the mt O$^6$-MeG MT protein is reacted with [$^3$H]-methylated DNA as substrate and run on SDS-PAGE gels a MT protein of ~22 Kd is seen, indicating that the MT isolated from mitochondria is probably transported from the cytosol as are many other proteins found associated with mitochondria.

### F.9 **The frequency of N-methyl-N-nitrosourea induced sister chromatid exchanges is reduced in mammalian cells expressing the *E. coli* O$^6$-guanine alkyltransferase gene**

C.H. Ockey, J. Brennand, G.R.M. White & G.P. Margison

*Paterson Laboratories, Christie Hospital, Manchester M20 9BX, UK*

Clones of Chinese hamster V79 fibroblast cells have been generated by transfection with retrovirus-based plasmids containing either the *E. coli* O$^6$-alkylguanine-alkylphosphotriester alkyltransferase gene (clone 8 cells) or a fragment of this gene which codes only for O$^6$-alkylguanine alkyltransferase (clone SB cells). These cells and clone 2 cells, which were transfected with the parent plasmid, were exposed to increasing doses of N-methyl-N-nitrosourea (MNU) and SCEs were scored. In clone 2 cells, known to be deficient in O$^6$-alkyltransferase, there was an almost linear dose response and at 8 $\mu$g MNU ml$^{-1}$ there were 45 SCEs per cell (more than 4 times background). In both clone 8 and clone SB cells SCE was only slightly higher than background level (~12 SCE per cell). These results suggest that O$^6$-methylguanine residues in DNA can give rise to SCE.

### F.10 **Lack of sequence homology between a fragment of *E. coli* DNA encoding an 0$^6$-methylguanine methyltransferase and the *ada* gene**

P.M. Potter, J. Brennand & G.P. Margison

*Paterson Laboratories, Christie Hospital, Manchester M20 9BX, UK*

During the isolation of the O$^6$-alkylguanine (O$^6$-AG) alkylphosphotriester (AP) dual alkyltransferase (AT) gene (*ada*) from an *E. coli* genomic DNA library, a second plasmid was identified that coded only for an O$^6$-AG AT activity (Margison *et al., Nucl. Acid. Res.,* **13,** 1939, 1985) and this DNA fragment (061) is being further characterised. Digestion with Bam HI, Eco RI, Hind III, Pst I or Sal I and agarose gel electrophoresis produced a pattern of subfragments that did not resemble that of similar digests of the *ada* gene or a section of it that codes only for the $O^6$-AG AT function. Southern analysis of total *E. coli* digested with Bam HI, Gbl II, Dra I, Eco RI, Hind III, Hpa II, Msp I, Pst I, or Sal I showed different bands using nick translated 061 or the dual function gene as a probe. Furthermore, the dual function AT gene did not hybridise to 061 DNA after digestion with Bam HI, Eco RI, Hind III, Pst I or Sal I. Subclones of 061 show greatly reduced AT activity but most results suggest that the coding region is in the 3′ end of the original 10 Kb 061 fragment. Biochemical and sequence analysis is under way but we tentatively conclude that *E. coli* contains a second gene for $O^6$-AG AT.

### F.11 Selective repair of methylated purines in regions of chromatin DNA

A.J. Ryan[1], M.A. Billett[1], & P.J. O'Connor[2]

[1]*Department of Biochemistry, University of Nottingham, Nottingham, UK;* [2]*Paterson Laboratories, Christie Hospital, Manchester M20 9BX, UK*

The distribution of methylated purines in different regions of liver chromatin DNA has been examined in rats treated with [$^{14}$C]-dimethylnitrosamine ($2\,mg\,kg^{-1}$). At various times later, liver nuclei were fractionated by micrococcal nuclease digestion and low and high salt extractions into an active chromatin fraction, two fractions comprising the bulk of the genome, and a nuclear matrix fraction. Regions of active chromatin and nuclear matrix were methylated more readily than bulk chromatin. The repair of N-methylpurines occurred relatively uniformly in all chromatin fractions whilst the repair of $O^6$-methylguanine proceeded more rapidly from active chromatin than from bulk chromatin and repair of this lesion from nuclear matrix DNA was much slower. Although pretreatment of rates with unlabelled dimethylnitrosamine enhanced the repair of $O^6$-methylguanine from all chromatin fractions, the rate of loss of this adduct was still faster from active chromatin and slower from matrix DNA, than for the bulk of the genome. Pretreatment also elevated the rate of DNA synthesis in the nuclear matrix fraction, thereby increasing the probability of the fixation of mutations in this selected region of the genome.

### F.12 Complementation of human DNA alkylation repair defects by *E. coli* DNA repair genes

L. Samson[1], B. Derfler[1] & E. Waldstein[2]

[1]*Laboratory of Toxicology, Harvard School of Public Health, Boston, MA 02115, USA;* [2]*Department of Biochemistry, G. Wise Faculty of Life Sciences, Tel Aviv University, Tel Aviv 69978, Israel*

The *E. coli ada-alkB* operon provides considerable protection against the effects of alkylation damage in bacteria. We have subcloned it into the pSV2 mammalian expression vector to yield pSV2*ada-alkB*, and this plasmid has been introduced into $Mer^-$ HeLa S3 cells which are highly sensitive to killing and SCE induction by alkylating agents. One transformant (the S3–9 cell line) has several integrated copies of the pSV2*ada-alkB* and expresses very high levels of the *ada* gene product, the 39kDa $O^6$-methylguanine-DNA-methyltransferase. S3–9 cells were found to be very resistant to killing and SCE induction by MNNG and BCNU. Hence bacterial DNA alkylation repair genes are able to complement alkylation repair defects in human cells. (Supported by American Cancer Society Research Grant NP448, Whitaker Health Sciences Fund Grant 85-13, NIH Cancer Research Grant CA35895 and US–Israel Binational Science Foundation Grant 3374.)

### F.13 Quantitative comparison of two promutagenic alkylated bases in mammalian DNA using radioimmunoassay

C.P. Wild[1], M. Serres[2], R.A. Becker[1], R. Saffhill[3] & R. Montesano[1]

[1]*International Agency for Research on Cancer, 150 Cours Albert Thomas, 69008 Lyon, France;* [2]*University of Lyon, INSERM-CNRS, U. 189, 69921 Oullins, France;* [3]*Paterson Laboratories, Christie Hospital, Manchester M20 9BX, UK*

The two minor methylation adducts $O^6$-methyldeoxyguanosine ($O^6$-medG) and $O^4$-methylthymidine ($O^4$-medT) have been implicated as initiating events in nitrosamine induced carcinogenesis. We have previously used a monoclonal antibody to analyze formation and persistence of $O^6$-medG in DNA from experimental animals and to detect this adduct in DNA from human surgical tissue specimens. We now report the production of a rabbit polyclonal antibody of affinity constant $10^9\,l\,mol^{-1}$, which allows detection 0.1 pmol $O^4$-medT in a RIA. Using a two-step HPLC fractionation, both $O^4$-medT and $O^6$-medG can be purified from the same DNA sample for RIA analysis. In *in vitro* alkylation repair studies mammalian tissue extracts, including human, have been shown to repair $O^6$-medG and $O^4$-medT but the above antibody methodology will facilitate the study of these adducts in the same tissue sample *in vivo* where little is known of their relationship in terms of formation and persistence. We have examined $O^6$-medG:$O^4$-medT ratios both *in vitro* in calf thymus DNA, and *in vivo* in rats after single or multiple exposure to alkylating agents. In addition we are examining human oesophageal and stomach DNA samples which were found to contain $O^6$-medG (Umbenhauer *et al.*, *Int. J. Cancer*, **36**, 661, 1985), for the presence of $O^4$-medT.

### F.14 **$O^6$-methylguanine-DNA-methyltransferase in human hepatic tissue**

A. Yawetz and E. Waldstein

*Institute for Nature Conservation Research and Department of Biochemistry at George S. Wise Faculty of Life Sciences, Tel-Aviv University, Israel*

The $O^6$-methylguanine-DNA-methyltransferase (MT) activity was particularly purified from human liver. The MT activity was present both in the cytosolic and nuclear fractions obtained by differential centrifugation of the homogenate. The activity in the nuclei was released by sonication, combined with the cytosolic fraction and precipitated between 25–55% saturation with ammonium sulfate. The MT activity was further purified 10-fold using DEAE-cellulose column chromatography (85% yield) and retained by double strand DNA cellulose affinity chromatography. The concentrated active material was further fractionated by gel filtration on Sepharose 6B. Most of the MT activity eluted as a narrow band with a mol. wt corresponding to 55,000. Only a minor fraction eluted at a mol. wt of 20,000–18,000. The withdrawal of glycerin resulted in an increase of absorbance (opening of the molecule) without concomitant change of mol. wt. Upon treatment with deoxycholate (0.1%) and Triton N101 (0.1%) most of the high mol. wt activity shifted to regions corresponding to mol. wt of 35,000 and 20,000. (Supported by NIH Cancer Research Grant CA35895 and US–Israel Binational Science Foundation Grant 3374.)

### G.1 **Alkaline step elution analysis of 8-methoxypsoralen photoinduced DNA crosslinks and their repair in yeast.**

E. Cundari & D. Averbeck

*Institut Curie—Biologie, Paris*

The characterization of interstrand crosslinks in yeast DNA induced by treatment with 8-methoxypsoralen (8-MOP) and UVA has been carried out by the alkaline step elution technique (Cundari and Averbeck, 1985). In the diploid strain D7 of *S. cerevisiae* crosslinks were revealed after cell lysis by a dose-dependent increase of double stranded DNA retained on elution filters at pH 12.5. To determine the number of crosslinks a given amount of DNA strand breaks was introduced by gamma-irradiation following 8-MOP plus UVA treatments. A linear relationship was observed between the residual radioactivity retained and the UVA dose (range 12–36 $kJm^{-2}$) at 5 $\mu$M of 8-MOP.

From alkaline elution analysis of the DNA from cells postincubated in growth medium after 8-MOP plus UVA treatment two phases can be distinguished for the repair of DNA interstrand crosslinks, (*a*) a rapid phase of incision as indicated by a decrease of residual radioactivity on the elution filters accompanied by the appearance of low molecular weight DNA fragments eluting at pH 11.5, (*b*) a slow phase in which these DNA fragments are apparently rejoined to high molecular weight DNA showing the same elution pattern as the DNA of untreated cells. Following treatments with 8-MOP plus UVA leaving ~90% survival more than 80% of crosslinked DNA was incised after 15 min. of postincubation while DNA strand rejoining was accomplished only after 2 h. The results show that diploid yeast is efficiently repairing 8-MOP plus UVA induced DNA crosslinks.

### G.2 **DNA-damage by the carcinogen 4-nitroquinoline 1-oxide: Structural identification and mutagenesis of the main adducts**

P. Daubersies, A. Heysen, S. Galiegue-Zouitina, B. Bailleul & M.H. Loucheux-Lefebvre

*Unité 124 INSERM, Institut de Recherches sur le Cancer de Lille, Place de Verdun, 59045 Lille, Cedex, France*

4-nitroquinoline 1-oxide (4 NQO) is a potent carcinogen the action of which is mediated by covalent interaction of its ultimate metabolite with DNA. 4-acetoxyaminoquinoline 1-oxide (Ac-4 HAQO) was proved to be an attractive model of ultimate carcinogen to study *in vitro* the carcinogenesis by this compound. The DNA-adducts were characterized as 4 NQO binding to N2 and C8 of guanine (60% and 30% of total modification, respectively) and to N6 of adenine leading to a minor adduct (see *Cancer Res.*, **45**, 520, 1985).

4 NQO is also a potent mutagen. Mutagenesis studies were initiated, in an *E. coli* system, for each of the two guanyl-adducts. The vector used was the tetracycline resistance gene of the plasmid pBR322 (see Fuchs *et al.*, *Nature*, **294**, 657, 1981). Plasmid DNA was especially modified at either N2 or C8 of guanine. As expected, there were significant differences in the behaviour of the two adducts. Indeed, the decrease in transforming efficiency of the N2-guanyl-modified DNA, on a wild *E. coli* strain, was much more important than those of the DNA containing the C8-guanyl adduct. Moreover, as reported by Fuchs *et al.* in the case of DNA-adducts of acetylaminofluorene (*J. Mol. Biol.*, **183**, 341, 1985), the mutants induced by the two NQO-adducts seemed qualitatively different.

### G.3 Nitropyrene induced DNA damage, toxicity and DNA-adduct formation in mammalian cells

M.J. Edwards, S. Batmanghelich, K. Smith & J.M. Parry

*School of Biological Sciences, University College of Swansea, Singleton Park, Swansea SA2 8PP, UK*

Nitropyrene and its chemically derived analogues were investigated for their cytotoxicity and DNA damaging activity in cultured Chinese hamster lung fibroblasts (Don: Wg3h). Both 1-nitrosopyrene and 1-aminopyrene (0.25–25 $\mu g\,ml^{-1}$) induced DNA single strand breaks and cell killing within 30 min of exposure. Higher doses of 1-aminopyrene (25–60 $\mu g\,ml^{-1}$) inhibited the formation of further DNA damage. 1-nitropyrene was not toxic and induced low levels of damage. The formation of DNA adducts was measured in calf thymus DNA which had been treated with nitropyrene or its analogues. Of the compounds investigated, only 1-nitrosopyrene formed a DNA-adduct without prior metabolic activation. In the presence of the mammalian nitroreductase, xanthine oxidase, both 1-nitropyrene and 1-nitrosopyrene formed one minor and one major DNA-adduct in calf thymus DNA. The major adduct was shown to be N-(deoxyguanosin-8-yl)-1-aminopyrene. Recently, an N-(deoxyguanosin-8-yl)-1-aminopyrene type DNA-adduct has been isolated from [$^{14}$C]-1-nitrosopyrene or [$^{14}$C]-1-nitropyrene treated Don cells. In contrast, a different DNA-adduct was isolated from Don cells which had been exposed to either [$^{14}$C]-1-aminopyrene or [$^{3}$H]-1.6 dinitropyrene.

### G.4 DNA damage and repair in cultured human fibroblasts exposed to 4 NQO or its 3 methyl derivative

S. Edwards & R. Waters

*School of Biology, University College, Swansea, UK*

The methylation of the potent carcinogen 4-nitroquinoline 1-oxide (4NQO) at the 3 position (3me 4NQO) dramatically reduces its genotoxic potency. Normal fibroblasts have been cultured on cytodex beads, exposed for 1 h to [$^{3}$H] 4NQO or [$^{3}$H] 3me 4NQO, their DNA purified and hydrolysed. Nuclease digests were subjected to chromatography on Bio-gel P-2. Undamaged deoxyribonucleosides were detected by absorption at 254 nm. Radioactivity in the resulting fractions was estimated. Peaks after 4NQO treatment have provisionally been characterised according to the elution profiles reported by Galiègue-Zouitina *et al.* (1985) for DNA after *in vivo* or *in vitro* exposure to 4-hydroxyaminoquinoline 1-oxide which is considered the proximate carcinogen of 4NQO. The profiles with 3me 4NQO are more difficult to analyse. First, no data are available to characterise the peaks, and second the effect of methylation on the elution of adducts is unknown. However, some features should be noted. First, the profile is not similar to that of 4NQO. Hence, the lower level of DNA damage seen with this agent at equimolar concentrations cannot be attributed to a low rate of demethylation of 3me 4NQO to give 4NQO. Second, as the chromatography separates the nucleosides on the basis of size, the largest molecules eluting last, the major 3me 4NQO lesion must be a breakdown product as it elutes before undamaged bases. Our next objectives are to characterise the 3me 4NQO peaks via mass spectrometry and to estimate the repair of these lesions in normal and XP fibroblasts.

### G.5 Mapping of psoralen adducts on defined DNA sequences

E. Sage & E. Moustacchi

*Institut Curie—Biologie, Paris, France*

Psoralens intercalate in the DNA double helix and form cyclo-addition products with pyrimidine bases upon UVA irradiation. The photoproducts result in furan-side and/or pyrone-side monoadducts. Furan-side monoadducts can be converted to diadducts by UVA light.

The aim of the work is to localize the psoralen-adducts in DNA fragments of defined sequences. We take advantage of the quantitative block of the 3′-5′-exonuclease associated with the T4 DNA polymerase at or near the site of bulky adducts. The stops of the exonuclease are examined on sequencing gel. The objective is to determine whether or not there are substantial differences in the DNA photoadducts formation between different mono and bifunctional psoralen derivatives.

Results concerning 8-MOP show the T4 DNA polymerase 3′-5′-exonuclease preferentially stops at or near *cross-linkable sites* (5′-TpA or 5′-ApT) and adjacent thymines. There is a marked effect of the flanking sequence upon the extent of formation of the photoproducts. Adducts at cytosine are not detected. Monoadducts do not exhibit hot-alkali sensitivity. The stop of the exonuclease at a particular site, exhibits multiple bands. This is probably due to the different types of adducts (furan or pyrone-side mono-adducts and crosslinks).

The bifunctional psoralen 5-MOP and monofunctional derivatives (angelicine, pyridopsoralens, 3-carbethoxypsoralen) are now tested. The effect of the wavelength of irradiation is also under investigation.

### G.6 A comparison of the genotoxic potencies of three aflatoxin $B_1$-dichloride induced modifications to plasmid DNA

M.L. Wood[1], J.R. Lindsay Smith[1] & R.C. Garner[2]

[1]*Department of Chemistry and* [2]*Cancer Research Unit, University of York, York YO1 5DD, UK*

The hepatocarcinogen aflatoxin $B_1$ ($AFB_1$) requires oxidative metabolism before it can exert its genotoxic effects. The active species is thought to be 8,9-dihydro-8,9-epoxy-$AFB_1$ ($AFB_1$-epoxide). Although this compound has not been isolated, 8,9-dichloro-8,9-dihydro-$AFB_1$ ($AFB_1$-$Cl_2$) provides a useful model due to the electrophilic nature of the carbon atom at position 8. As expected, this compound is a direct-acting mutagen and carcinogen.

We have shown that $AFB_1$-$Cl_2$ reacts with DNA *in vitro* to produce an unstable $N^7$-substituted guanine adduct ($AFB_1$-Cl-G), which behaves identically to the equivalent $AFB_1$-$N^7$-guanine adduct ($AFB_1$-G), i.e. $AFB_1$-Cl-G can break down to form a more stable imidazole ring-opened adduct (iro-$AFB_1$-Cl-G) or undergo spontaneous depurination to leave an apurinic site on the DNA molecule. Binding of $AFB_1$-$Cl_2$ to plasmid DNA (followed by transformation of the plasmid into repair deficient bacteria) has allowed us to study which of these 3 lesions is most important in $AFB_1$ induced mutagenesis. The results obtained indicate that both guanine adducts are equally mutagenic to the bacteria, whereas apurinic sites on the plasmid DNA lead to reduced levels of mutation. The reported instability of $AFB_1$-G *in vivo* ($t\frac{1}{2}$=*ca.* 20 h in rat liver) suggests that iro-$AFB_1$-G is the $AFB_1$-induced lesion with greatest biological significance.

### G.7 Poly(ADP)ribosylation is involved in depression of semiconservative DNA synthesis after 8-Methoxypsoralen + UV*A*

E. Wunder[2], & J. Heise[1]

[1]*Institut fur Humangenetik, Heidelberg, Germany;* [2]*Institut Curie, Paris, France*

Human cells in culture show after treatment with radiations or 8-Methosypsoralen(8-MOP)+UV*A* a typical pattern of change in the rates of semi-conservative DNA synthesis (SDS). After an initial drop, rates pass a minimum and increase slowly leading to resumption of pretreatment values after several hours. In normal human fibroblasts treated with 8-MOP+UV*A* we found by autoradiographic examination, that the fraction of cells incorporating [$^3$H]TdR stayed the same as in untreated cells. This indicates that the change of SDS rates takes place in every single cell. If 3-Aminobenzamide is added immediately after 8-MOP photoaddition, the decline in rate of DNA synthesis is aggravated compared to treatment with 8-MOP+UV*A* alone The recovery phase still occurs however with similar kinetics. Consequently poly(ADP)-ribosylation of nuclear proteins is involved in the cellular response to 8-MOP photoadduct as in the case of treatment with Dimethylsulfonate, but not by $\gamma$ and UVC irradiation (James & Lehmann, *Biochem.* **21,** 4001, 1982).

### H.1 The roles of *recA* in UV mutagenesis in *E. coli*

B.A. Bridges, R. Woodgate & M. Ruiz-Rubio

*MRC Cell Mutation Unit, University of Sussex, Falmer, Brighton, Sussex BN1 9RR, UK*

In addition to its role as a protease able to cleave *lexA* repressor protein and so derepress *din* genes such as *umuD,C* and *recA* itself, *recA* protein also has a direct involvement in the mutagenic process (Blanco *et al.*, *Biochimie*, **64,** 633, 1982). *RecA430* bacteria are non-mutable by UV yet are able to carry out the misincorporation step (seen as the induction of mutations by delayed photoreversal of UV irradiated *recA430* bacteria). It would therefore appear that *recA* protein is required for the bypass step in what may be proteolytic or quasi-proteolytic interaction with *umuD* protein.

The *recA441* allele confers a higher frequency of misincorporation and this is probably a non-proteolytic function since it is unaffected by temperature, by adenine or by guanosine plus cytidine. Although Fersht and Knill-Jones (*J. Mol. Biol.*, **165,** 669, 1983) and Echolls (personal communication) have evidence that *recA* protein may affect the proofreading function of DNA polymerase III holoenzyme *in vitro*, there is as yet no clear proof that it is essential for misincorporation *in vivo*, although it clearly can influence the amount of misincorporation that takes place.

### H.2 Molecular analysis of ouabain-resistant mouse cells

J. Cole, W.J. Muriel & A.R. Lehmann

*MRC Cell Mutation Unit, University of Sussex, Falmer, Brighton, E. Sussex BN1 9RR, UK*

The cardiac glycoside ouabain is a specific inhibitor of the plasma-membrane $Na^+$, $K^+$-ATPase and it has been assumed that ouabain resistant mutants arise as a consequence of alterations in the ATPase such that ouabain is no longer able to bind. It has recently been demonstrated however that ouabain-resistance can also be mediated by a completely separate ouabain-inducible and ouabain-resistant $K^+$-transport system. We have isolated 20 ouabain-resistant mutants from the mouse lymphoma L5178Y cell line including spontaneous mutants, and induced mutants following treatment with a number of different mutagens. None of these mutants had a ouabain inducible $K^+$ uptake system, as measured by the uptake of radioactive rubidium following a 24 h treatment with ouabain. The uptake of rubidium in the absence of inducing treatment was however much more resistant to ouabain in all the mutants than in wild-type cells. Gross alterations in the structure of two ouabain-resistance genes, coding respectively for the ouabain-resistant $K^+$ uptake system and for the $Na^+$, $K^+$-ATPase, have been investigated by Southern analysis. No deletions, rearrangements or amplifications of either of these genes were detected in any of the mutants. The possibility of over-expression of these genes is currently under investigation. At present our results are most consistent with the original hypothesis of that the majority of ouabain-resistant mutants do indeed result from base-changes in the $Na^+$, $K^+$-ATPase gene.

### H.3 Ultraviolet mutagenesis in excision-proficient *umuC* and *lexA*(ind$^-$) *Escherichia coli* as revealed by delayed photoreversal

B.A. Bridges & F. Sharif

*MRC Cell Mutation Unit, University of Sussex, Falmer, Brighton, Sussex BN1 9RR, UK*

Streptomycin-resistant mutations are induced in excision-proficient *umuC122*::Tn5 bacteria given delayed photoreversal after UV light. The mutations occur after much earlier photoreversal (10–20 min) than is found in excision-deficient *umuC* bacteria and the yield of mutants is between 25 and 40% of that found immediately after UV irradiation of isogenic *umu*$^+$ bacteria. Mutagenesis is not inhibited by the presence of chloramphenicol after UV and before photoreversal. Loss of photoreversibility of streptomycin-resistant mutations in *umu*$^+$ excision-proficient bacteria also occurs during a similar period after UV and is similarly unaffected by chloramphenicol. The results are interpreted on a 2-step model of error-prone repair in which a misincorporation step is followed by a lesion bypass step which requires induced levels of *umuC* gene product, the latter step being unnecessary when the pyrimidine dimer is removed by photoreversal after the misincorporation has taken place. It is suggested that loss of photoreversibility in excision-proficient *umu*$^+$ bacteria may reflect the misincorporation step only, thus explaining the apparent noninducible nature of loss of photoreversibility previously reported.

### H.4 Site-specific mutagenesis by $O^6$-methylguanine and hypoxanthine

M. Hill-Perkins, M.D. Jones & P. Karran

*Imperial Cancer Research Fund, Lincoln's Inn Fields, London WC2A 3PN, ICRF Clare Hall Laboratories, Potters Bar, Herts EN6 3LD, UK*

The technique of site-directed mutagenesis has been used to investigate the mutation induction by single DNA lesions at a specific locus in M13mp9 DNA. Covalently closed duplex circular molecules which contained a single altered purine base were constructed *in vitro* and transformed into competent *E. coli.* $O^6$-methylguanine did not induce a significant frequency of mutations in progeny phage. However, the mutant frequency was greatly enhanced by exhausting the cellular repair enzyme for this base before transformation. In contrast, hypoxanthine induced mutations in the absence of any prior interference with cellular repair enzymes. This indicates that *E. coli* hypoxanthine-DNA glycosylase acts inefficiently in the removal of hypoxanthine from DNA *in vivo.* Both $O^6$-MeG and hypoxanthine induced transition mutations.

### H.5 Localized conversion in *Streptococcus pneumoniae* transformation: sequence specificity

J.-C. Lefevre, P. Mostachfi, A.-M. Gasc, P. Garcia, D. Baty & M. Sicard

*Centre de Recherche de Biochimie et Génétique Cellulaires du CNRS, 118 route de Narbonne, 31062 Toulouse, France*

In pneumococcal transformation, we have described recently an aberrant marker (*amiA*36) in the *amiA* locus that appeared to enhance recombination frequency when crossed with any other allele of this gene (Lefevre, *et al., Proc. Natl Acad. Sci.,* **81,** 5184, 1984). This hyperrecombination is due to a frequent (20%) conversion to wild type (Sicard, *et al., Genetics,* **110,** 557, 1985). The aberrant mutation results from a transversion in the sequence 5′,ATTCAT,3′, generating 5′,ATTAAT,3′ and spans over very few nucleotides. We have constructed artificial heteroduplexes using separated DNA strands: only one of the heteroduplexes 5′,ATTAAT/3′,TAAGTA is converted. Using a suppressor gene, we have crossed this mutation by a closely linked mutation to isolate double mutants. Their frequency shows that conversion *amiA*+→*amiA*36 is as likely as the reciprocal conversion *amiA*36→*amiA*+.

This 6-base palindrome has been created in another region of this locus by directed mutagenesis using synthetic oligomers. This new mutation is also conversinogenic. Thus, this special 6-base heteroduplex structure is sufficient to induce conversion.

### H.6 Suppression of a nonsense mutation in human cells *in vivo* by aminoglycoside antibiotics

A.E. Mogg, L.A. Heywood & J.F. Burke

*School of Biological Sciences, Biochemistry Laboratory, University of Sussex, Brighton BN1 6QG, Sussex, UK*

Aminoglycoside antibiotics in *E. coli* and yeast can cause ribosomes to read through stop codons during translation. This can result in the phenotypic suppression of nonsense mutations.

In order to determine the degree of aminoglycoside suppression of nonsense mutations in mammalian cells *in vivo* we have used the mammalian cell transfection vector $pRSVcat_{amb38}$. The plasmid contains the bacterial gene chloramphenicol acetyl transferase (*cat*) with nonsense codons at positions 27 and 38. The gene is transcribed from the Rous Sarcoma Virus long terminal repeat when transfected into Human 293 cells, but the level of CAT activity is less than one thousand times that of cells transfected with the wild type vector, pRSV*cat*.

We have tested two aminoglycosides G-418 and Paromomyacin for their ability to suppress the nonsense codons. In each case we have demonstrated that these antibiotics stimulate significant read through of all three classes of nonsense mutation *in vivo.*

### H.7 UV-induced mutation fixation occurs before resumption of DNA replication

I. Pietrzykowska & M. Felczak

*Institut of Biochemistry and Biophysics, Pol. Acad. Sci., Warszawa, Poland*

We have previously shown that mutation in gene *umu*C does not affect resumption of DNA replication which is inhibited for 30 min in UV-irradiated *E. coli* cells, but does affect later stage(s) of replication. We have also noted the absence of any correlation between time of appearance of UV-

induced mutants in wild type strain and *umu*C-dependent inhibition of DNA replication (~20 min and 90 min post-UV, respectively. Abstr. 16th FEBS Meeting, Moscow, 1984, p. 405).

New results indicate that UV-induced mutants are formed before resumption of DNA replication inhibited by UV-irradiation. We have studied the time-course of DNA replication resumption in UV-irradiated *E. coli uvr*A and *uvr*A*rec*F strains, and appearance of $his^+$ revertants. In the *uvr*A strain, the maximal level of mutants was observed after 15–20 min incubation in rich medium after UV, while DNA replication was inhibited for 30 min. This suggested that restoration of DNA replication requires a higher level of induced SOS functions than mutagenesis. Using an *rec*F mutant, known to show delayed and lowered level of induced SOS functions, we show that mutation fixation may occur before resumption of DNA replication in UV-irradiated cells. Control experiments excluded the possibility of DNA replication on the selective plates before expression of $his^+$ mutations.

### H.8 AFB1C12 induced frameshift mutagenesis in *E. coli*

L.M. Refolo, C.B. Bennett & M.Z. Humayun

*Department of Microbiology, New Jersey Medical School, Newark, NJ, USA*

Our approach to an investigation of AFB1C12-induced frameshift mutagenesis was to construct two derivatives of M13mp8 each containing a frameshift within the $lacZ_\alpha$ gene. The derivative BK8 is expected to revert by a −1 (±3N) bp event, whereas HS8 is expected to revert by a +1 (±3N) bp event. RFI DNA was modified *in vitro* with AFB1C12, transfected into *E. coli* and $lacZ_\alpha^+$ revertants were isolated and sequenced. Our results indicate that; (1) AFB1C12 induces 2 classes of BK8 frameshift revertants, a simple class (−1 bp event) representing 90% of all induced mutations and a complex class (a −1 bp event associated with a nearby base substitution) representing the remaining 10%; (2) the simple mutations are *rec*A independent; (3) SOS induction enhances the occurrence of simple mutations 3–10 fold; (4) AFB1C12 significantly enhanced the occurrence of simple mutations at *14* of 40 revertable sites, the 5 "hottest" mutational sites involve those G:C bp with the highest predicted AFB1C12 reactivity; (5) complex mutations are significantly enhanced in strains carrying the $muc^+$ plasmid pGW270. This effect requires a *recA* ± gene; (6) AFB1C12 induces 2 classes of HS8 revertants, a simple class (a + 1 bp event) representing 65% and a complex class (a + 1 bp event accompanied by a nearby base substitution) representing 35% of induced mutations; (7) More than 50% of HS8 base additions (including base substitutions) involve insertion of an A:T bp. We propose that these misinsertions reflect a lesion directed targeted process. Furthermore, we suggest that complex mutations occur via a concerted mechanism (two synchronous events) in which a single lesion leads to a targeted and "untargetted" event.

### H.9 Confirmation of an intermediary complex in UV-mutagenesis: Specificity of mutation by delayed photoreactivation in *umu*C cells

M. Ruiz-Rubio, R. Bockrath & B.A. Bridges

*MRC Cell Mutation Unit, University of Sussex, Falmer, Brighton, Sussex BN1 9RR, UK*

Mutagenesis by ultraviolet radiation (UV) in *Escherichia coli* is blocked in *umu*C defective cells but reappears after delayed photoreactivation (PR). This could result if intermediary complexes accumulate at pyrimidine dimers in DNA, allowing misincorporation but not continued replication. Removal of the dimers by PR then would release the complexes and let DNA synthesis establish permanently the errors that occurred specifically at dimers. We describe UV reversion of two auxotrophic defects (UAA at *his* and *arg* gene sites) by backmutations and by tRNA suppressor mutations. Neither backmutation results from delayed PR of irradiated *umu*C cells, and both backmutations are insensitive to PR in *lexA51 recA441* cells which are genetically induced and activated for UV-mutagenesis. Contrariwise, glutamine tRNA suppressor mutations do result from delayed PR of *umu*C cells and are sensitive to PR in *lexA51 recA441* cells. Thus the idea of an intermediary complex accumulating in *umu*C cells and released by delayed PR associates specifically with mutation targeted at pyrimidine dimers. (Supported in part by NIH grant GM21788.)

### H.10 Effect of *dnaE486ts* and *mutD5* mutations on UV mutagenesis in *E. coli*

M. Ruiz-Rubio, R. Woodgate & B.A. Bridges

*MRC Cell Mutation Unit, University of Sussex, Falmer, Brighton, Sussex BN1 9RR, UK*

The *dnaE486* allele specifies an alpha subunit of DNA polymerase III holoenzyme that confers a spontaneous mutator effect presumably due to either defective base selection or defective proofreading. The *mutD5* mutator allele specifies a defective epsilon subunit which has a marked defect in proofreading. The *mutD5* allele has no effect either on UV mutagenesis, or on UV mutagenesis seen after delayed photoreversal of *umu*C or *recA430* bacteria. Proofreading is therefore not responsible for the stopping of DNA polymerization at or before photoproducts in the template strand nor does the deficiency in proofreading appear to affect any stage in UV mutagenesis. If DNA polymerase III is involved, the epsilon subunit must be largely non-functional during the mutagenic repair event.

*DnaE486* similarly has no effect on delayed photoreversal mutagenesis in *umu*C bacteria but has a pronounced UV mutator effect in strains able to carry out *umu*C, D functions. We suggest that this polymerase has difficulty in reestablishing its fidelity after mutagenic synthesis has occurred opposite a photoproduct, leading to a burst of untarget mutations associated with, but not opposite photoproducts ("hitch-hiking" mutations).

### H.11 A new role for photoreversible pyrimidine dimers in induction of prototrophic mutations in excision-deficient *Escherichia coli* by ultraviolet light

M. Ruiz-Rubio[1], R. Woodgate[1], B.A. Bridges[1], G. Herrera[2] & M. Blanco[2]

[1]*MRC Cell Mutation Unit, University of Sussex, Falmer, Brighton, Sussex BN1 9RR, UK;* [2]*Instituto de Investigaciones Citologicas, Amadeo de Saboya, 4, Valencia 10, Spain.*

UV mutagenesis to His$^+$ in certain *recA441 lexA51* bacteria is not photoreversible indicating pyrimidine dimers are not target lesions. Photoreversibility is observed in *recA*$^+$ *lexA51* bacteria showing pyrimidine dimers are needed to activate *recA*$^+$ protein (unlike *recA441* protein) to perform a function in UV mutagenesis distinct from cleavage of *lexA* repressor.

### H.12 UV-induced mutagenesis in the *cro* gene of bacteriophage λ carried on a multicopy plasmid

H. Shwartz, O. Landman & Z. Livneh.

*Department of Biochemistry, Weizmann Institute of Science, Rehovot 76100, Israel*

We have developed a new mutagenesis system based on the *cro* repressor gene of bacteriophage λ. The repressor is overproduced from pHS27, a multicopy plasmid, and represses the expression of the *lacZ* gene which is under the control of $O_RP_R$ operator-promoter of λ in our tester strains. The assay detects mutations in *cro* which reduce its binding to $O_R$, thereby allowing partial or full expression of *lacZ*. The system is based on screening and is thus free of selection pressures. All types of mutations can be detected, including base substitutions, frameshifts, deletions and rearrangements.

Transformation of the tester strains with UV-irradiated plasmid pHS27 demonstrated the production of UV-induced mutations in *cro*. These mutations are increased by UV-irradiating the host strains prior to transformation. Transformation of UV-irradiated cells with the unirradiated plasmid showed an increased frequency of mutations, demonstrating the existence of untargeted mutagenesis in this system.

### J.1 The DNA double-strand break origin of chromosomal aberrations

P.E. Bryant

*Department of Anatomy and Experimental Pathology, University of St Andrews, St Andrews KY16 9TS, Fife, UK*

The relationship between DNA double-strand breaks (dsb) and chromosomal aberrations has been investigated using a model approach (Bryant, *Int. J. Radiat. Biol*, **46**, 57, 1984) in which Sendai virus permeablized V79 Chinese hamster cells were exposed to various type II restriction endonucleases. These enzymes induced dsb in the nuclear DNA having a variety of specific end structures. It was shown that "blunt-ended" dsb from Pvu II, Alu I or Eco RV induce chromosomal aberrations in a dose dependent way, whereas cohesive-ended dsb with an overlap of 4 bases (e.g. from Bam H1 or Eco R1) did not yield aberrations above spontaneous levels. Experiments with V79 cells synchronized at the G1/S border indicate a progressive decline in efficiency of aberration induction as base overlap was increased from zero to four. The results support the molecular "breakage-first" hypothesis of Bender *et al.* (*Mutation Res.*, **23**, 197, 1974) and open up a new approach to the investigation of the molecular mechanisms of chromosomal aberration induction.

### J.2 Poly(ADP-ribose) requirement for cell cycle traverse

B.W. Durkacz, A.L. Harris & K. Moses

*Cancer Research Unit, Royal Victoria Infirmary, Newcastle Upon Tyne NE1 4LP, UK*

Inhibitors of ADP-ribosyl transferase (ADPRT) are known to inhibit DNA repair and enhance the cytotoxicity caused by monofunctional alkylating agents. Using a number of ADPRT inhibitors, we have shown that ADPRT function is also required for cell cycle traverse. CHOK1 cells treated with ADPRT inhibitors block in G1 or G2, thereby protecting against the cytotoxicity of the S-phase acting drugs, hydroxyurea and 5-fluorodeoxy-uridine, in the presence of uridine. Using serum deprivation to synchronise CHOK1 cells, we have selectively blocked cells in G1 or G2 by treatment with inhibitors. We demonstrate that while cells can reversibly arrest at the G1 block, the G2 block rapidly becomes cytotoxic. There is a good correlation between the relative potency of the inhibitors as blockers of cell cycle traverse and as inhibitors of ADPRT. This indicates that the inhibition of cell cycle traverse is mediated via inhibition of ADPRT function. The use of the ADPRT-mediated cell cycle block to protect against the cytotoxicity of S-phase acting drugs as a selective system for the isolation of ADPRT defective mutants is discussed.

### J.3 Hypoxanthine-DNA glycosylase from *E. coli* and human thymus

I. Harosh & J. Sperling

*Department of Organic Chemistry, The Weizmann Institute of Science, Rehovot 76100, Israel*

Hypoxanthine-DNA glycosylase from *E. coli* and human thymus were partially purified and characterized. The enzymatic activity was assayed by following the release of [$^3$H]hypoxanthine from nick-translated DNA containing [$^3$H]dIMP residues. The enzymatic activity from both sources has an obligatory requirement for $Mg^{++}$ ions, and is inhibited in the presence of EDTA. Other divalent metal ions can only partially replace $Mg^{++}$.

The human thymus-enzyme was purified about 500 fold by fractionation on DEAE-cellulose, phosphocellulose P-11 and second chromatography on DEAE-cellulose. NaCl and KCl have an inhibitory effect, while caffeine does not affect the enzymatic activity.

The hypoxanthine-DNA glycosylase from *E. coli* has been extensively purified and characterised. We have found that the enzyme has a mobility of a 56-kd polypeptide, as determined by SDS-polyacrylamide gel electrophoresis, and it migrates as a 60-kd polypeptide in gel filtration under native conditions. The sedimentation coefficient of the enzyme was determined by glycerol gradient centrifugation and was found to be 4.0 S. The Km of the *E. coli* enzyme is $4.2 \times 10^{-5}$ M, and that of the human thymus enzyme is $7.4 \times 10^{-5}$ M.

### J.4 The analysis of replicating instabilities in the fission yeast *Schizosaccharomyces pombe*

J.L. Roberts & B.J. Kilbey

*Department of Genetics, University of Edinburgh, West Mains Road, Edinburgh EH9 3JN, UK*

A replicating instability is defined as a heritable premutational lesion in the DNA of an organism which leads to repeated and identical mutations at a locus over many generations. Replicating instabilities were discovered in Drosophila by Auerbach (*Proc. Roy. Soc. Edin. B*, **62**, 307, 1947) after exposure of male flies to mustard gas, their nature remains unelucidated. Similar phenomena have been reported in *Escherichia coli* (R.F. Hill, *J. Gen. Microbiol*, **30**, 289, 1963), *Neurospora crassa* (Burnett & de Serres, *Genetics*, **48**, 717, 1963) but most work has been done with fission yeast (Nasim & James, *Genetics*, **69**, 513, 1971; Loprieno *et al.*, *Genet. Res.*, Camb., **12**, 45, 1968). The object of the present investigation was to study the molecular nature of replicating instabilities by cloning and sequencing the products of instabilities induced by EMS at loci concerned with adenine biosynthesis in *S. pombe*.

This contribution describes our attempts to detect *bona fide* replicating instabilities in *S. pombe* and raises questions concerning the adequacy of criteria previously used to identify them. It would appear that they are far less frequent than previously proposed in fission yeast.

### J.5 **Use of a postlabeling method to identify the thymine glycols in poly(dT) and lambda DNA oxidized by hydrogen peroxide.**

J.F. Mouret, F. Odin, J. Cadet & M. Polverelli

*CEN-Grenoble DRF, Chimie 85X, 38041 Grenoble Cedex, France*

Thymine glycols have been shown to be the predominant oxidation products of thymine as the results of the action of hydroxyl radicals and various oxidizing agents such as osmium tetroxide and permanganate. Quantitation of these base lesions when generated in low yields remains a challenging problem.

This is due in particular to self-radiolysis processes which give rise to significant amounts of thymine diols when prelabeled DNAs are used as oxidation substrates. This drawback may be avoided by the use of a postlabeling technique (Bodell *et al.*, *Anal. Biochem.*, **142**, 525, 1984) allowing the incorporation of $^{32}P$ into the modified nucleotides after enzymatic digestion with appropriate enzymes.

Thymine glycols were found in poly(dT) oxidized by hydrogen peroxide in carefully deionized solutions in order to prevent the Fenton reaction.

An authentic sample of thymidine glycol monophosphate was chemically synthesized for comparison with products obtained from enzymatic digestion of the oxidized substrates.

Special effort was made to check the exonuclease digestion of the thymidine glycol monophosphate from modified oligonucleotide. Work is in progress to measure thymine glycol in cellular DNA.

### J.6 **Benzamides at nanomolar concentrations stimulate nuclear ADP-ribosylation**

C.J. Skidmore & J. Jones

*Department of Physiology & Biochemistry, University of Reading, PO Box 228, Whiteknights, Reading RG6 2AJ, UK*

Investigation of the role of the nuclear protein modification ADP-ribosylation in DNA repair has depended heavily on the use of benzamides as inhibitors of ADP-ribosyltransferase (ADPRT) in intact cell studies. In general benzamides inhibit DNA repair-dependent processes but a number of anomalous effects have been observed. We have found that, in permeabilised L1210 cells, 3-acetoamidobenzamide inhibits ADP-ribosylation at concentrations above 10 $\mu$M but stimulates the reaction at nanomolar concentrations. A number of ADPRT inhibitors show these effects. Both stimulation and inhibition appear to be due to binding of the compound at similar, if not identical, sites. When ADP-ribosylation is separated into its component reactions—initiation and elongation—stimulation of only the initiation reaction is observed. These effects occur under conditions which may well obtain in intact cells and stress the importance of careful controls to demonstrate directly that the extracellular concentration of inhibitor applied is sufficient to inhibit ADP-ribosylation inside the cell.


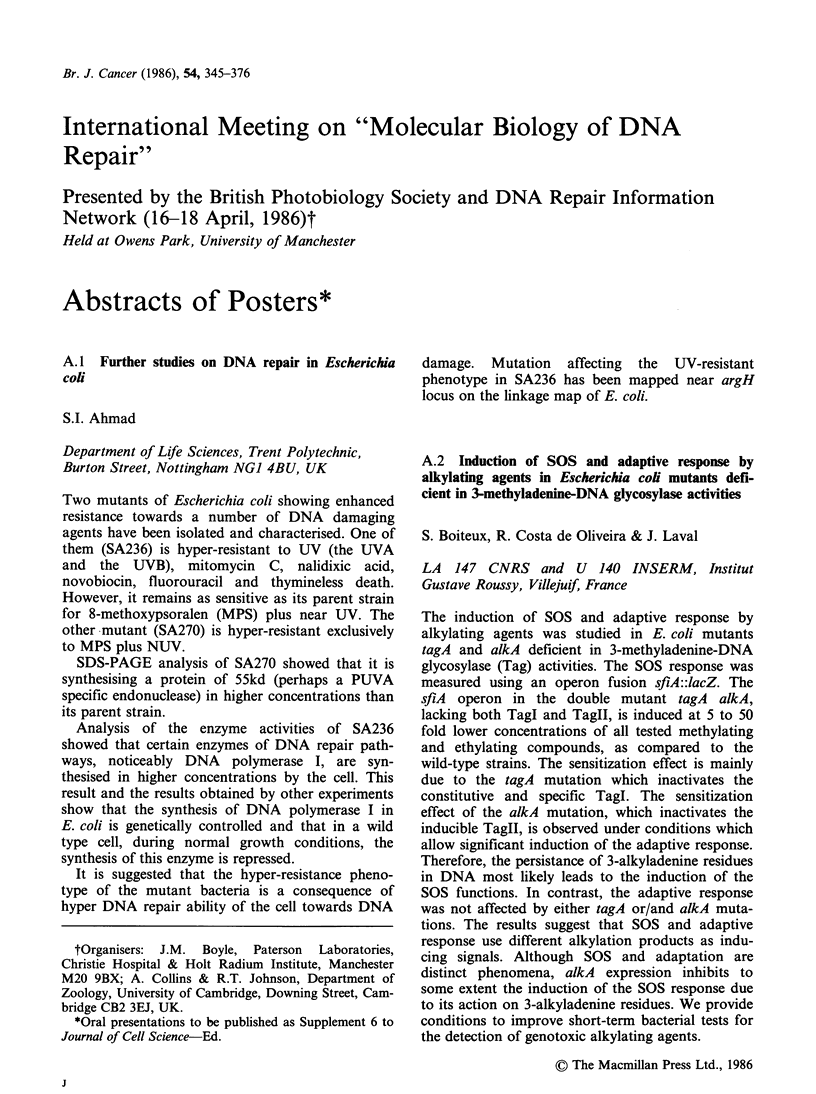

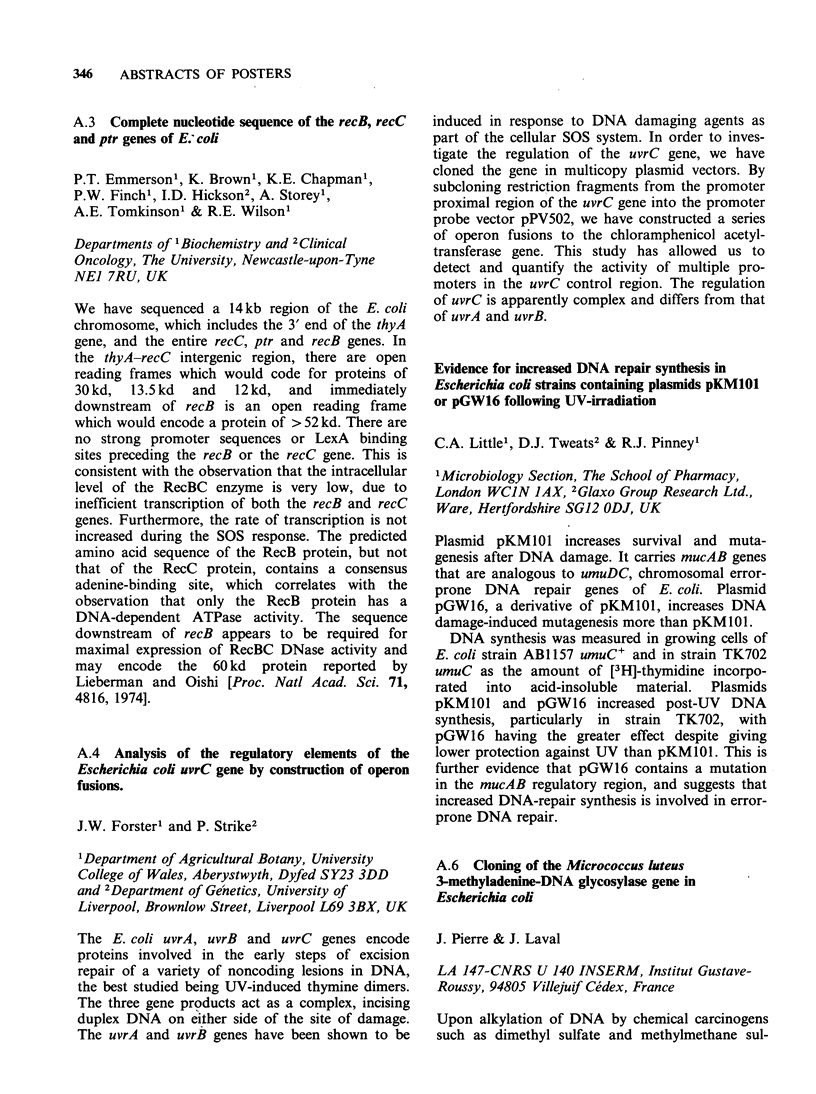

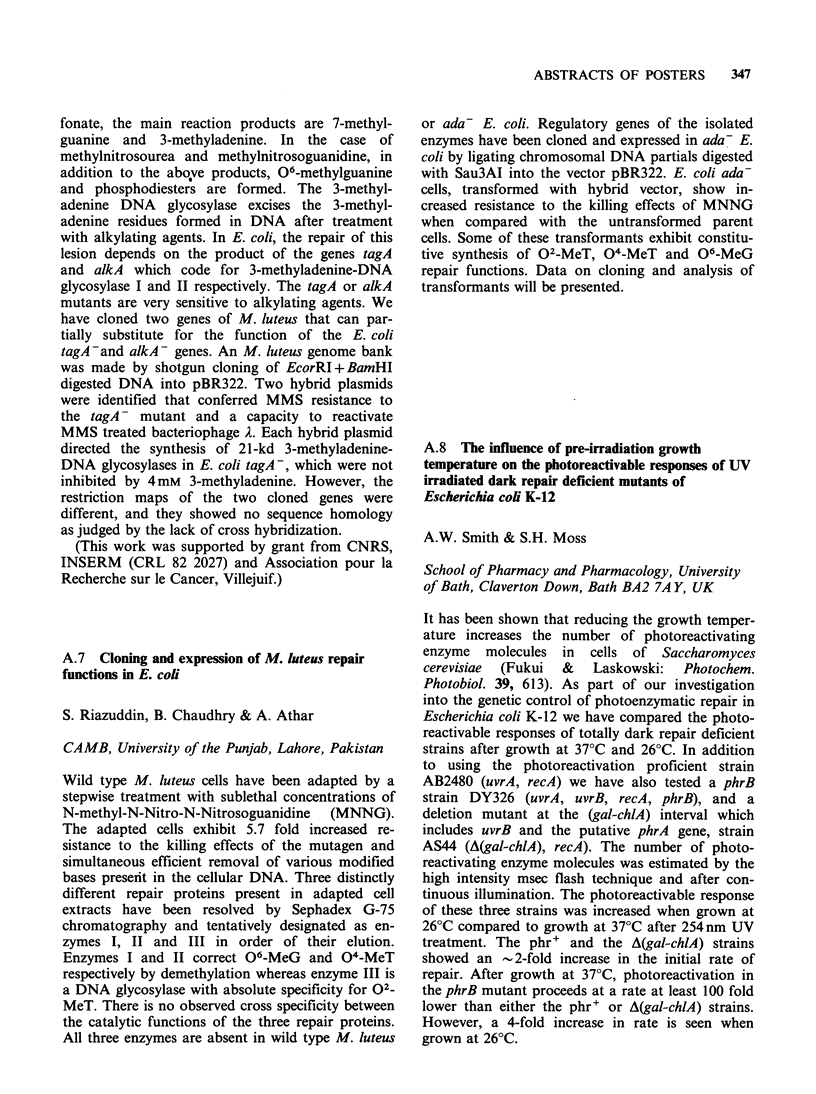

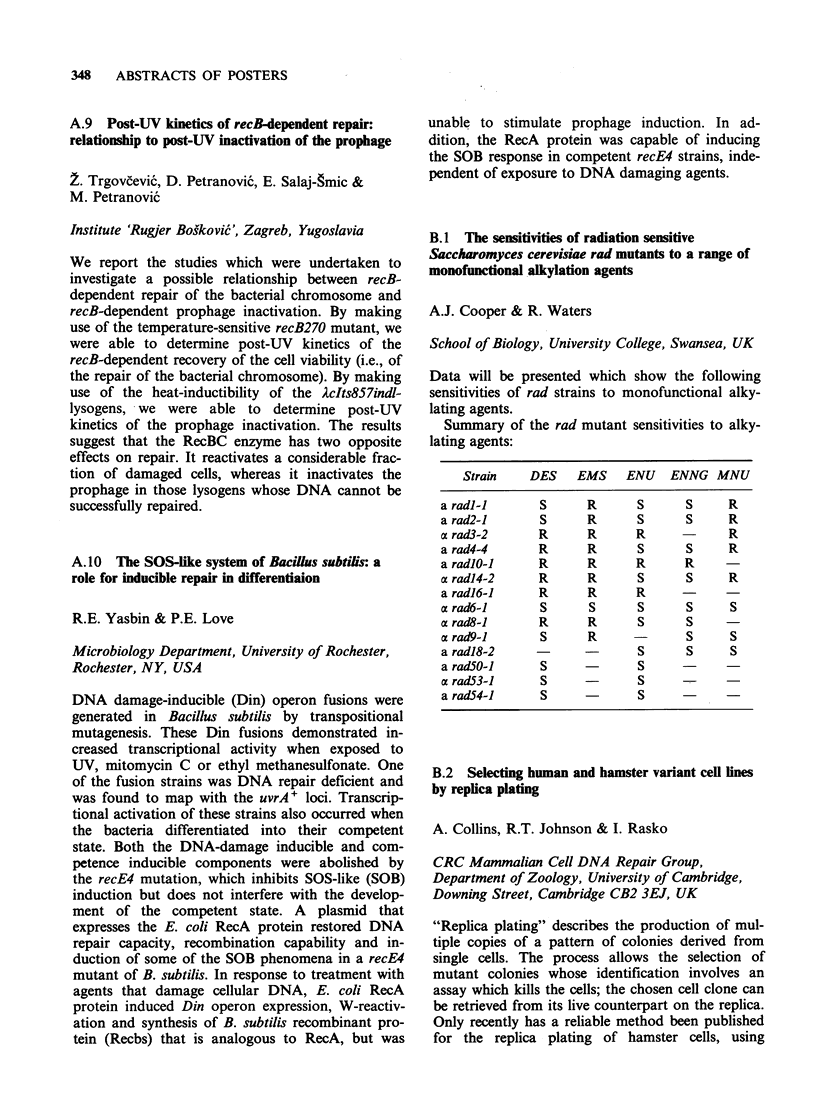

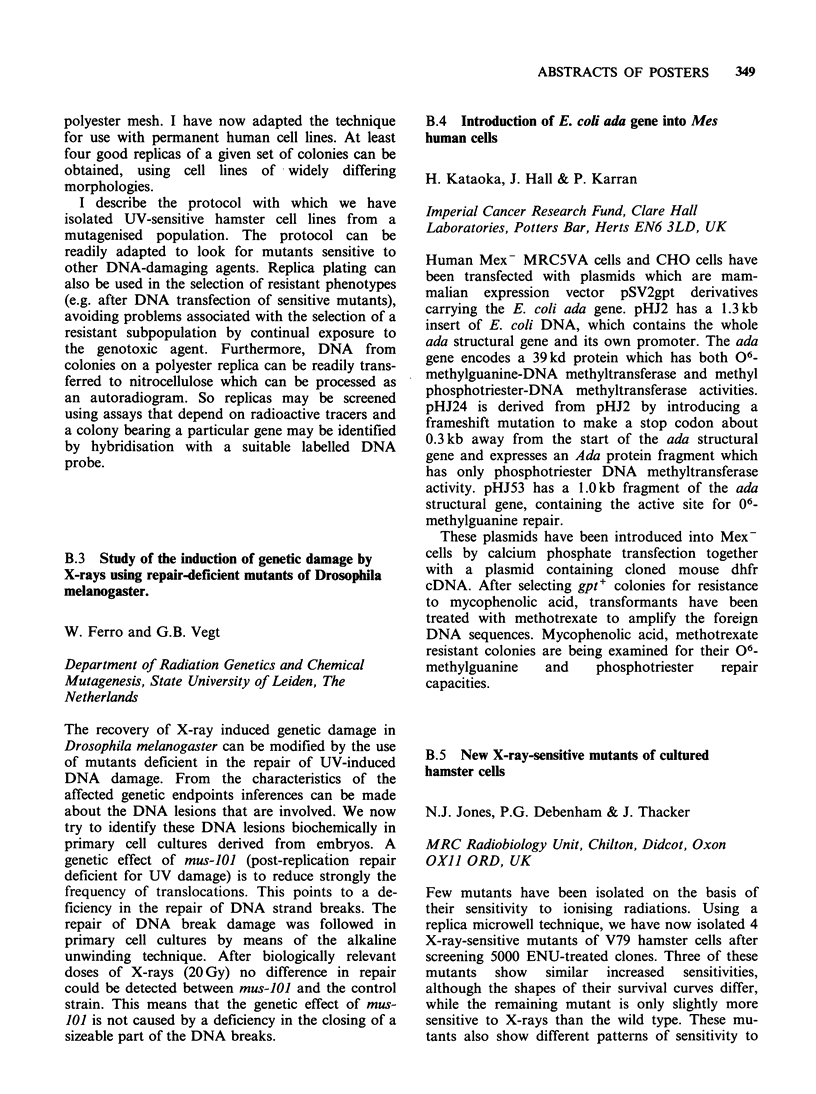

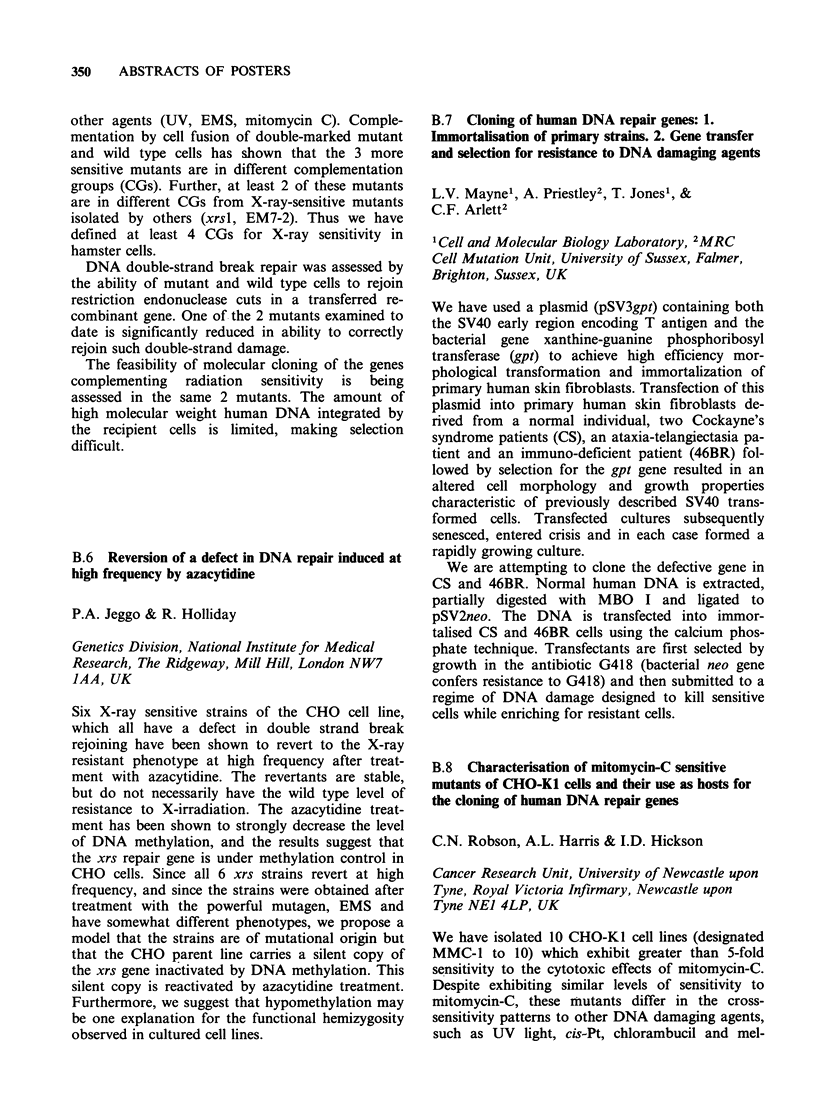

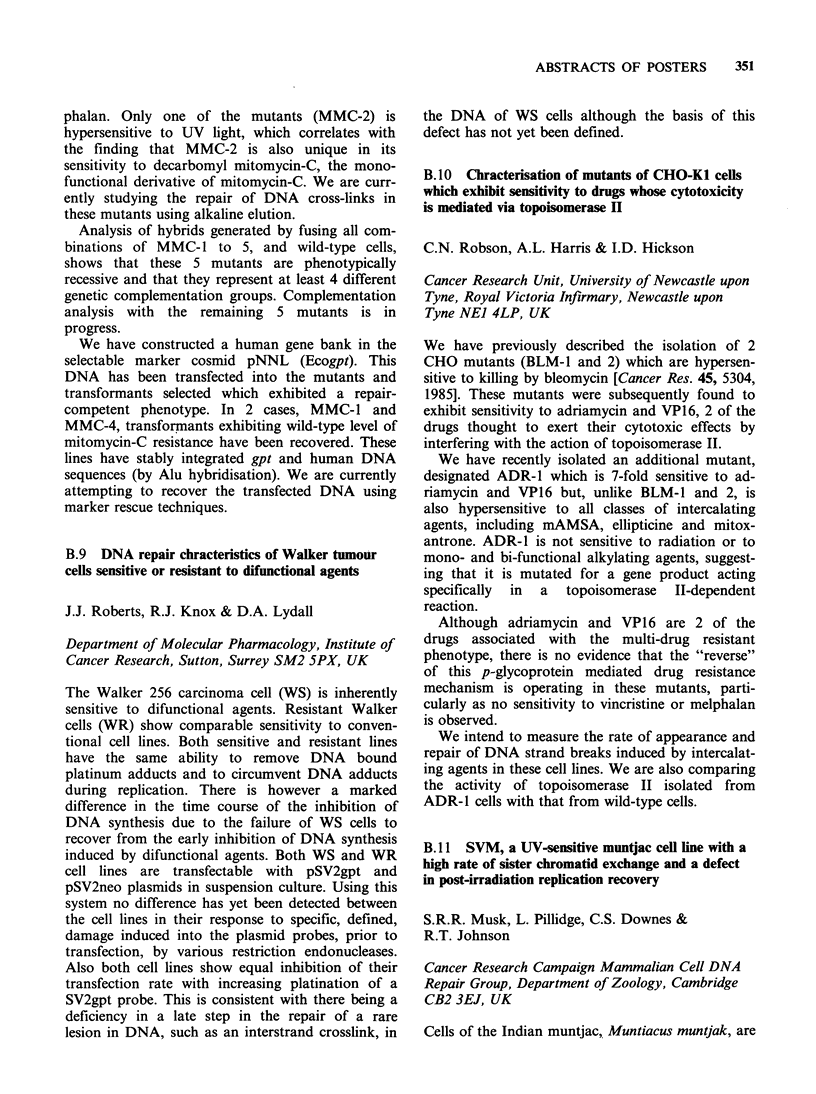

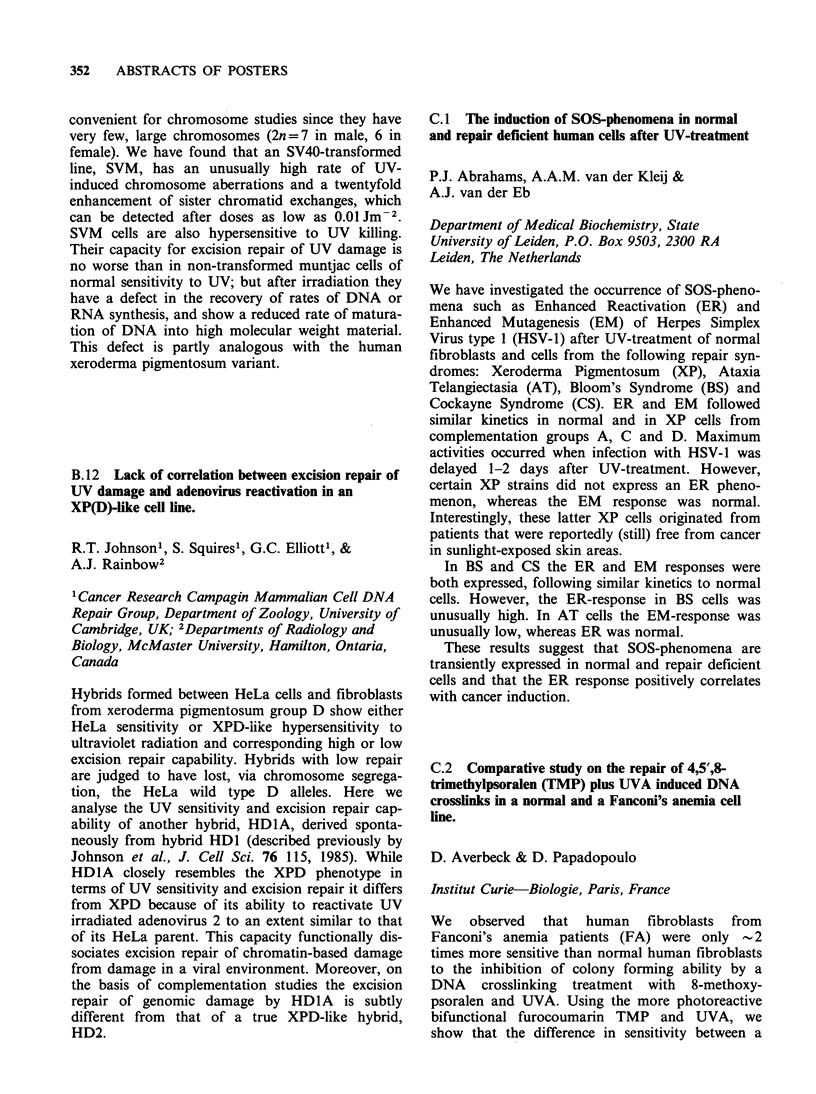

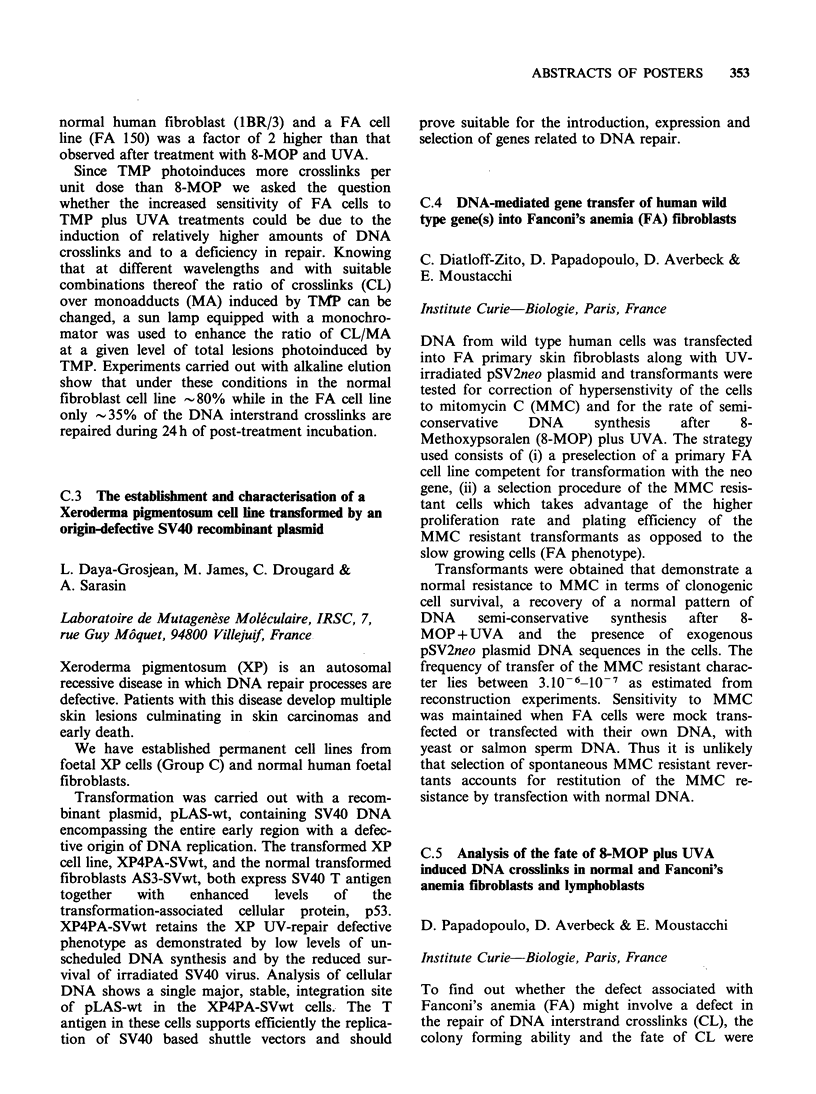

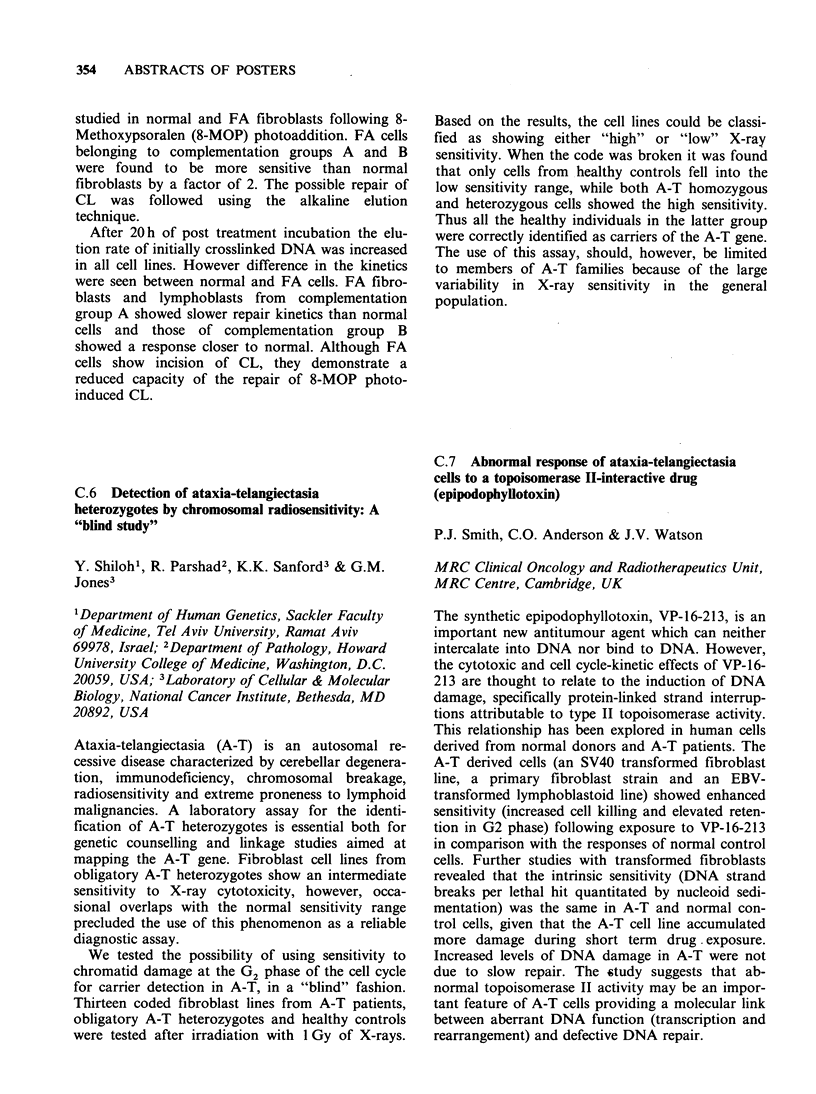

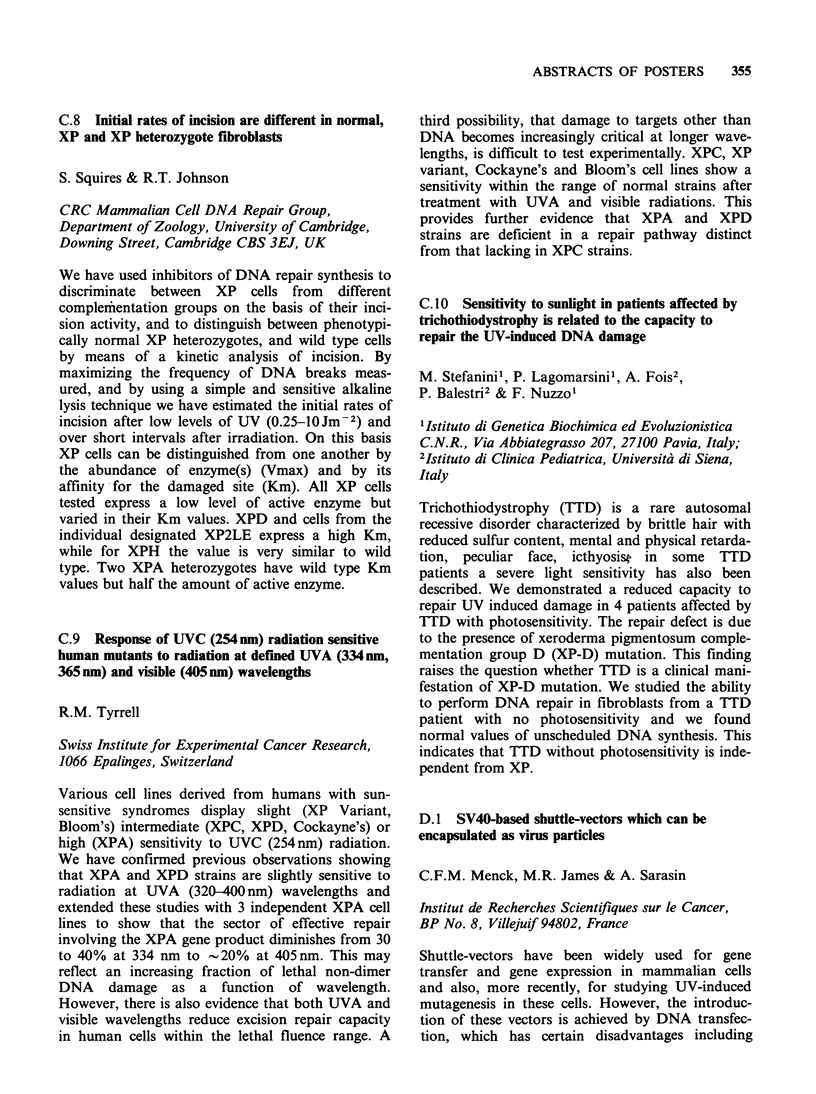

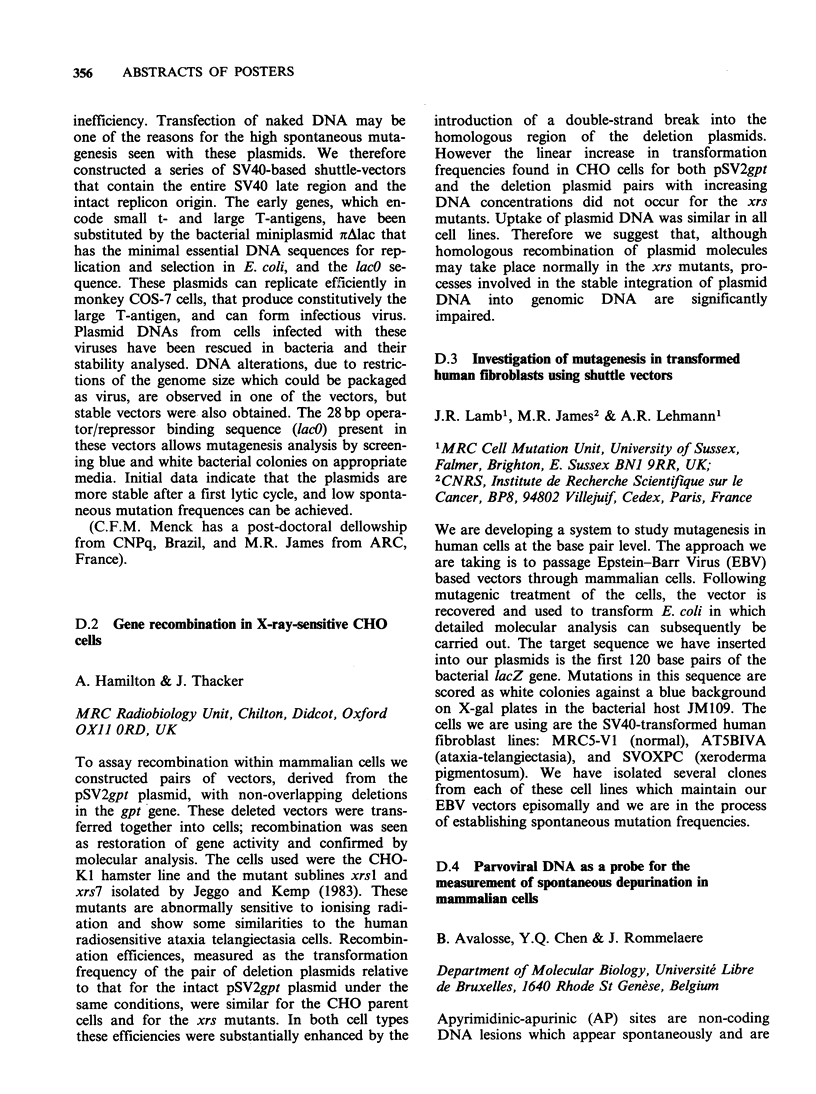

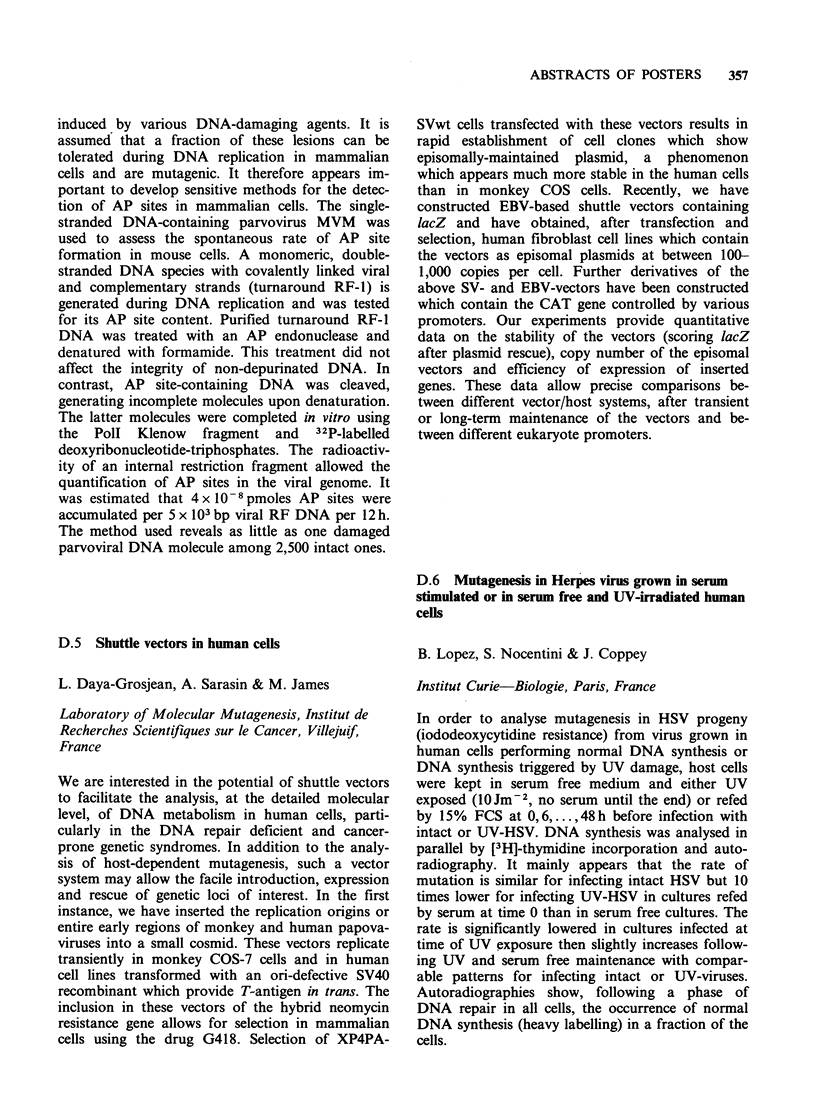

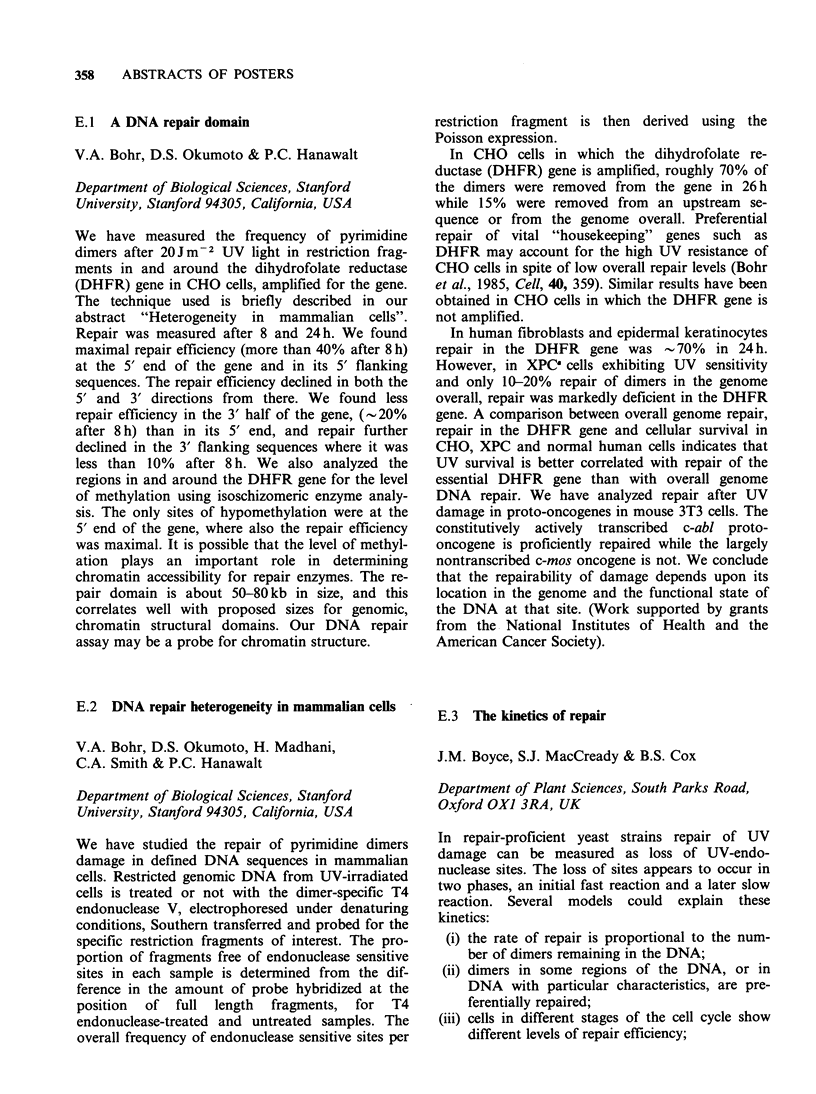

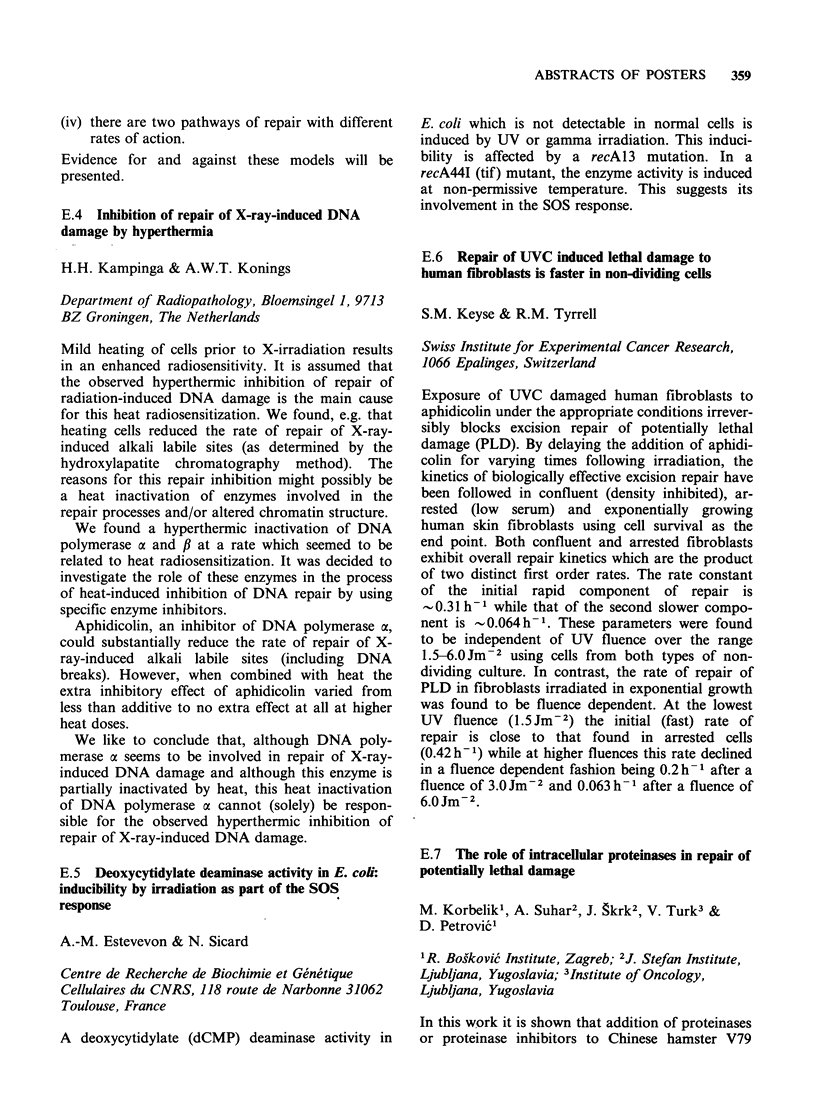

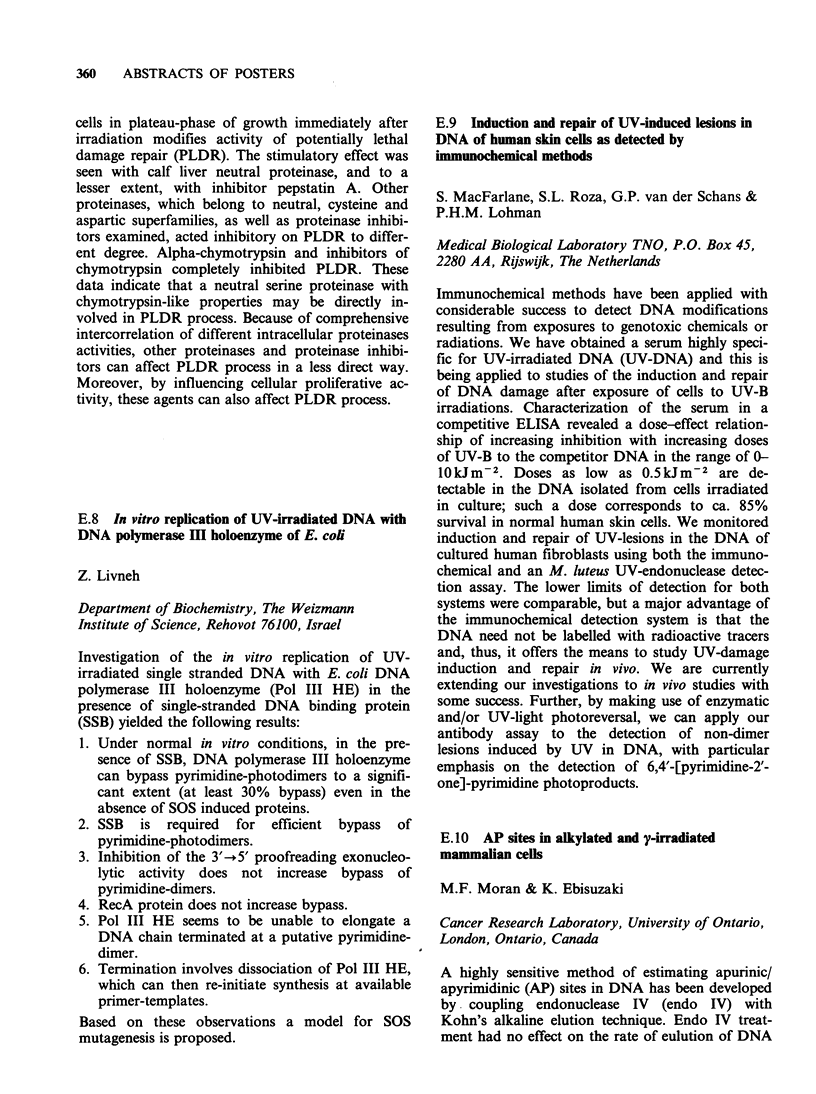

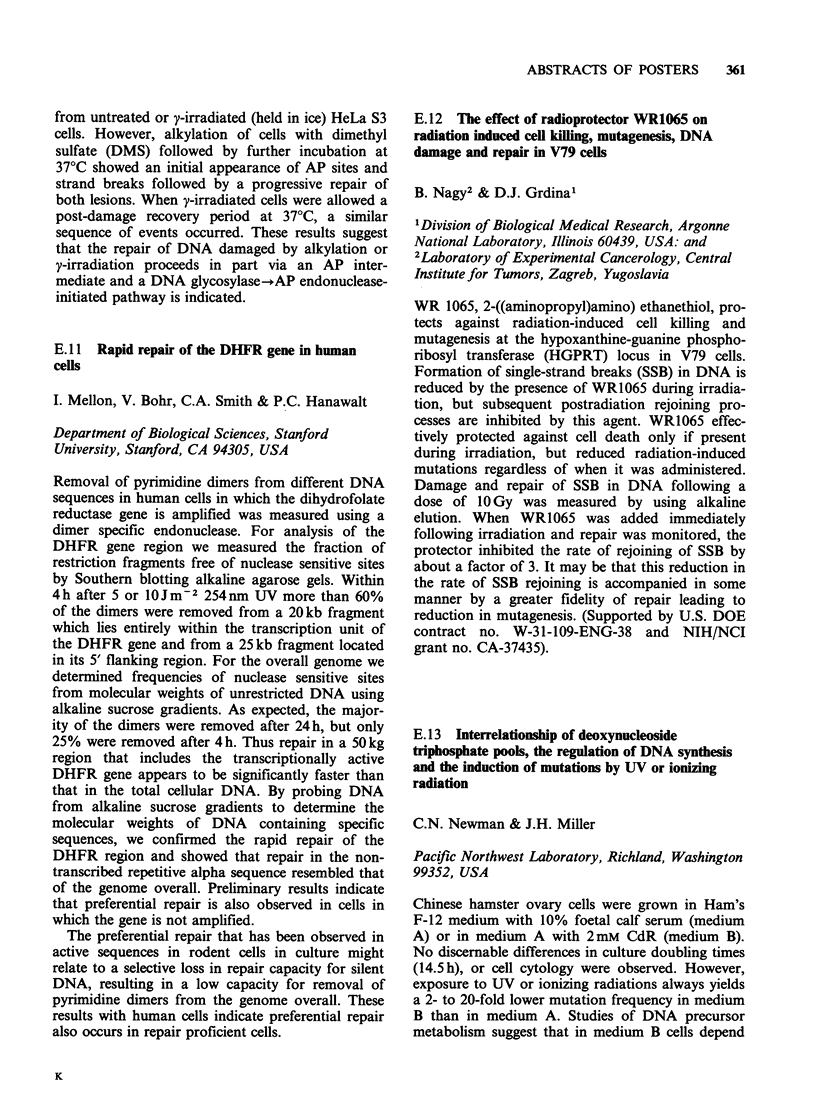

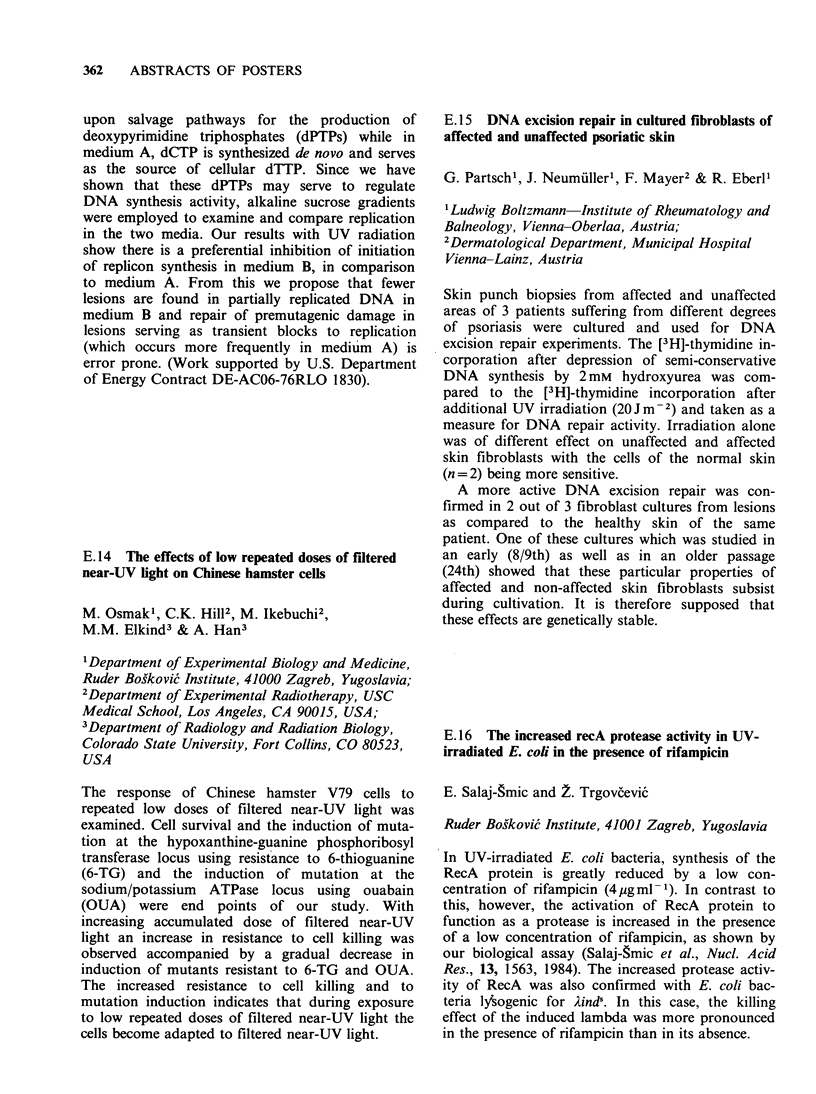

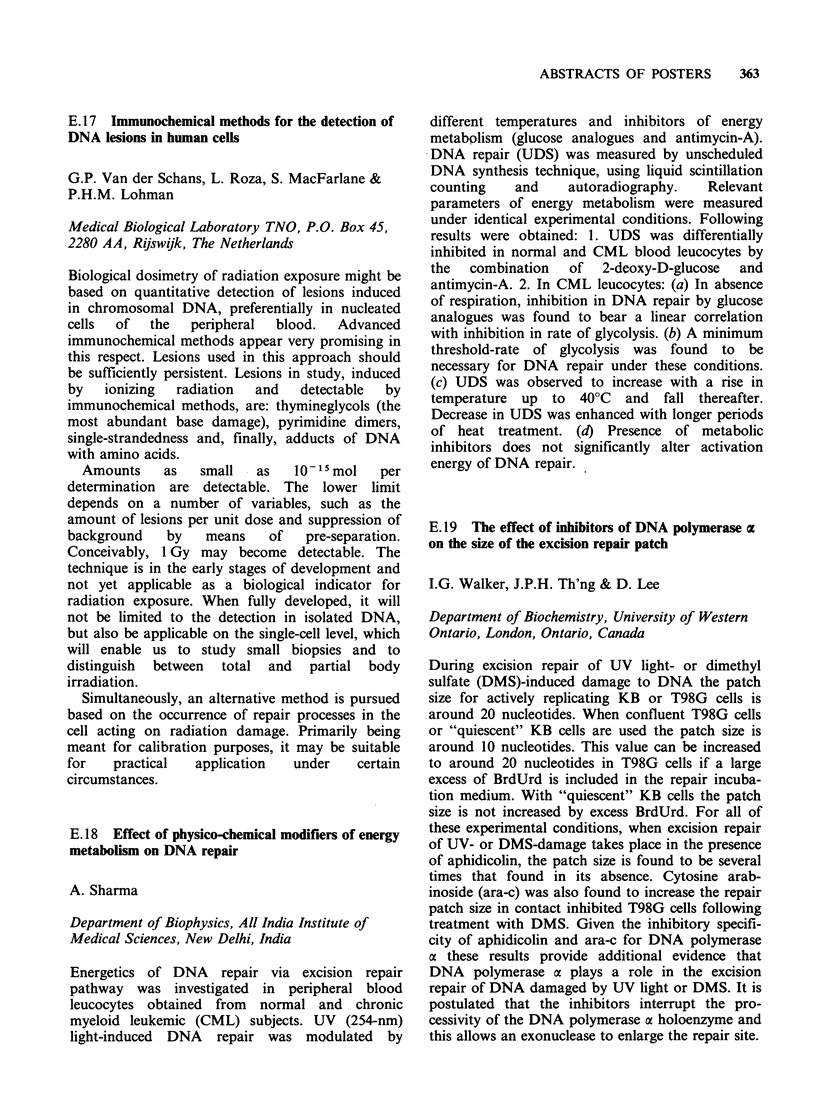

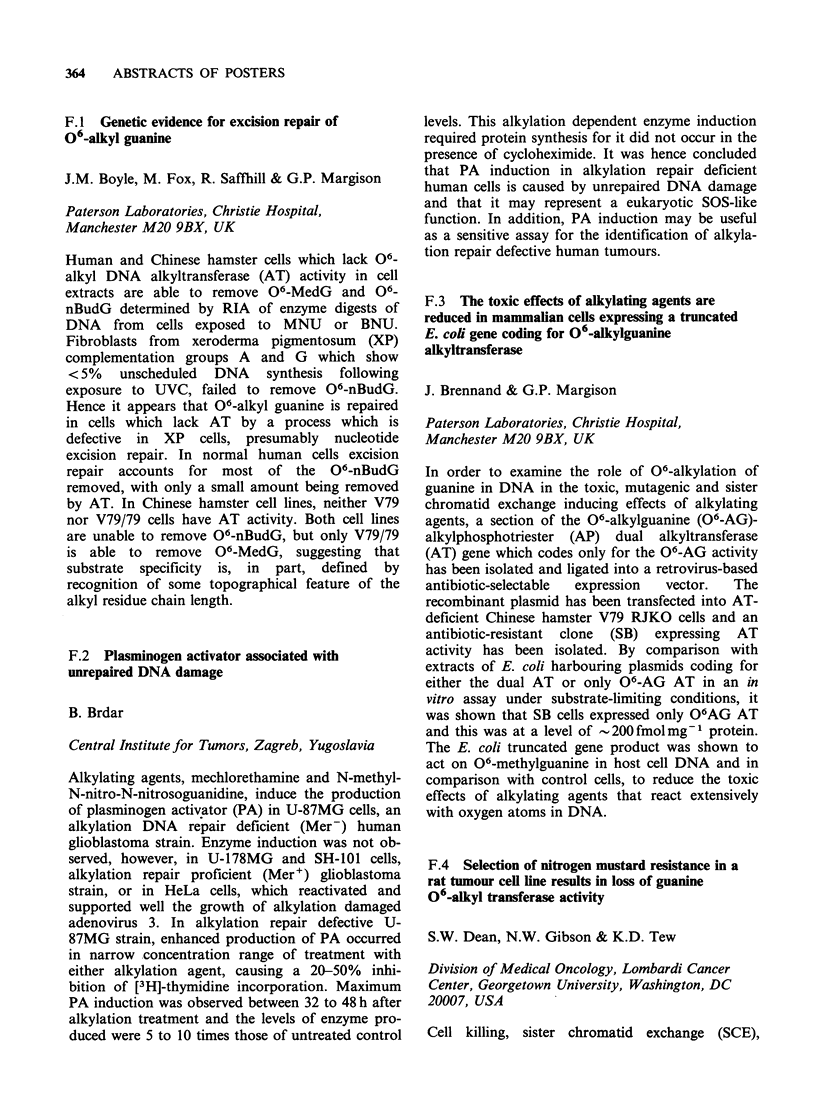

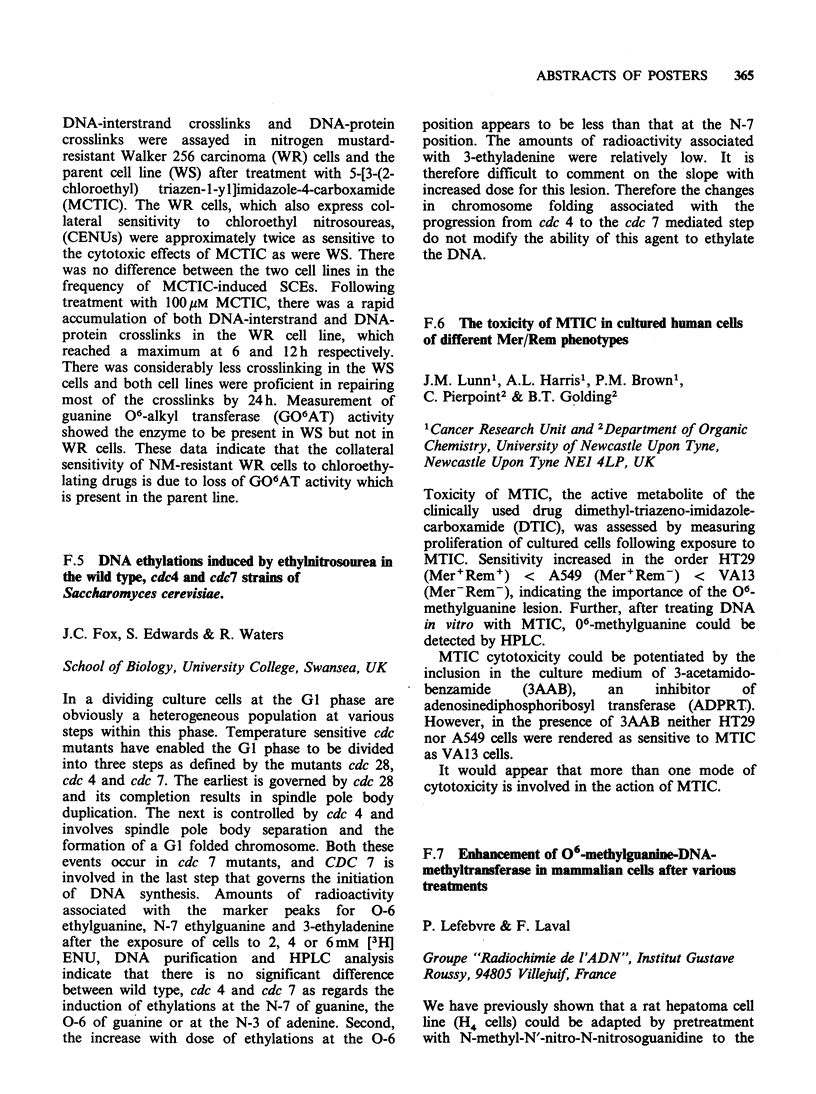

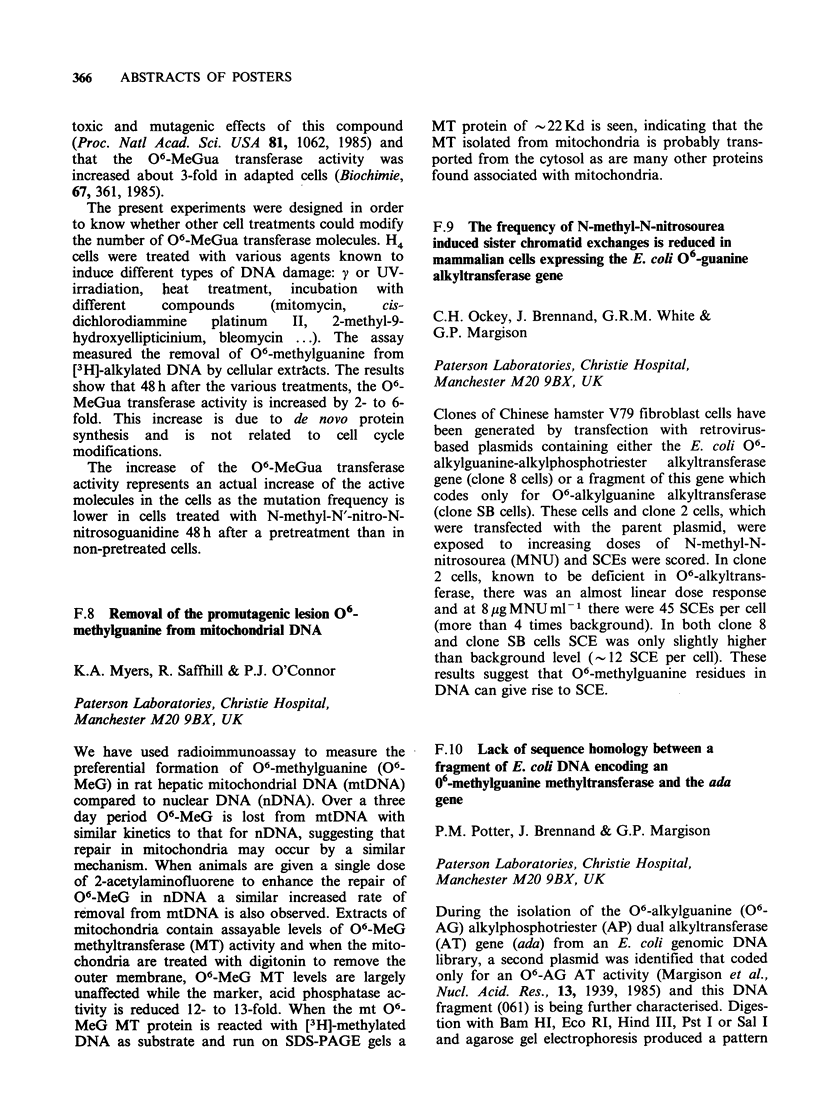

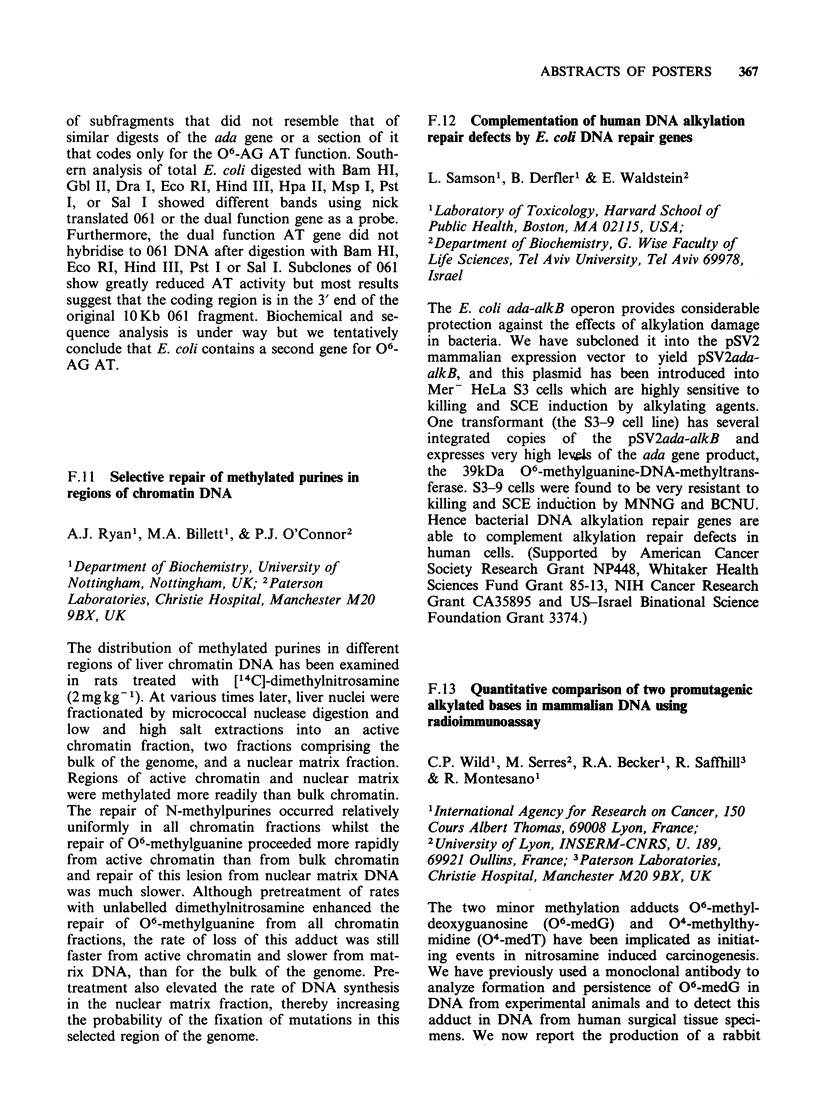

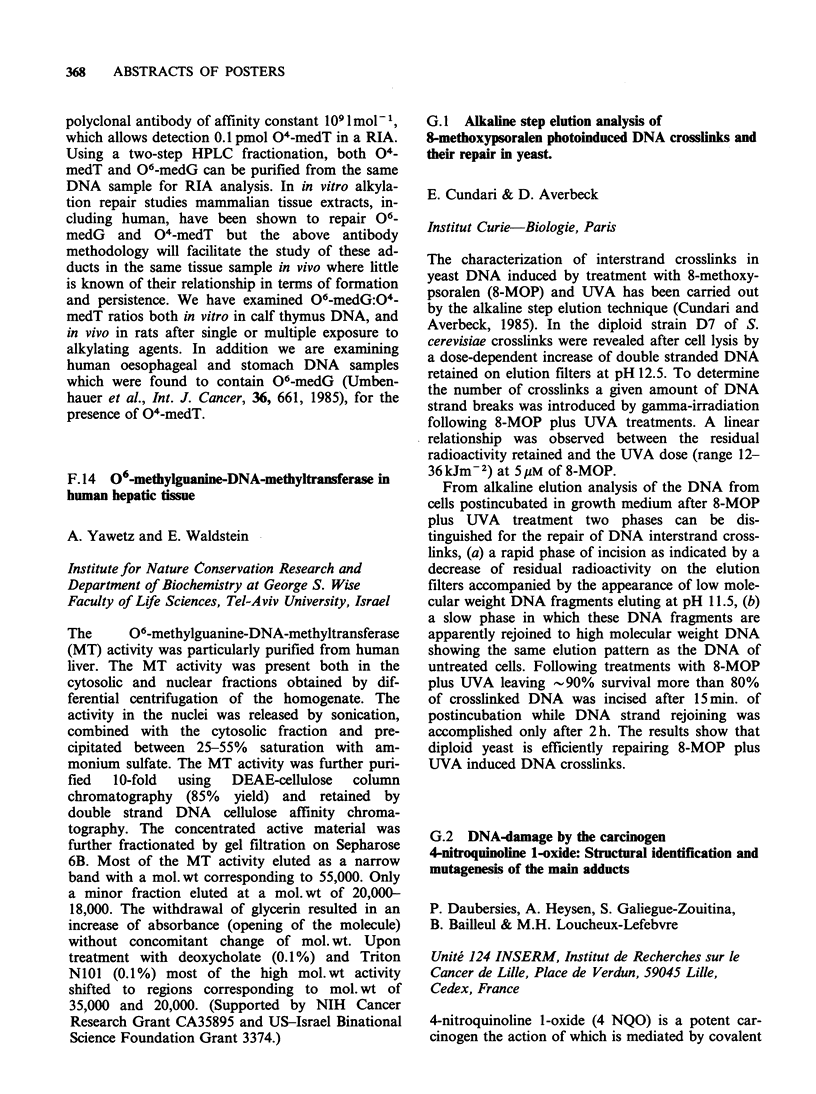

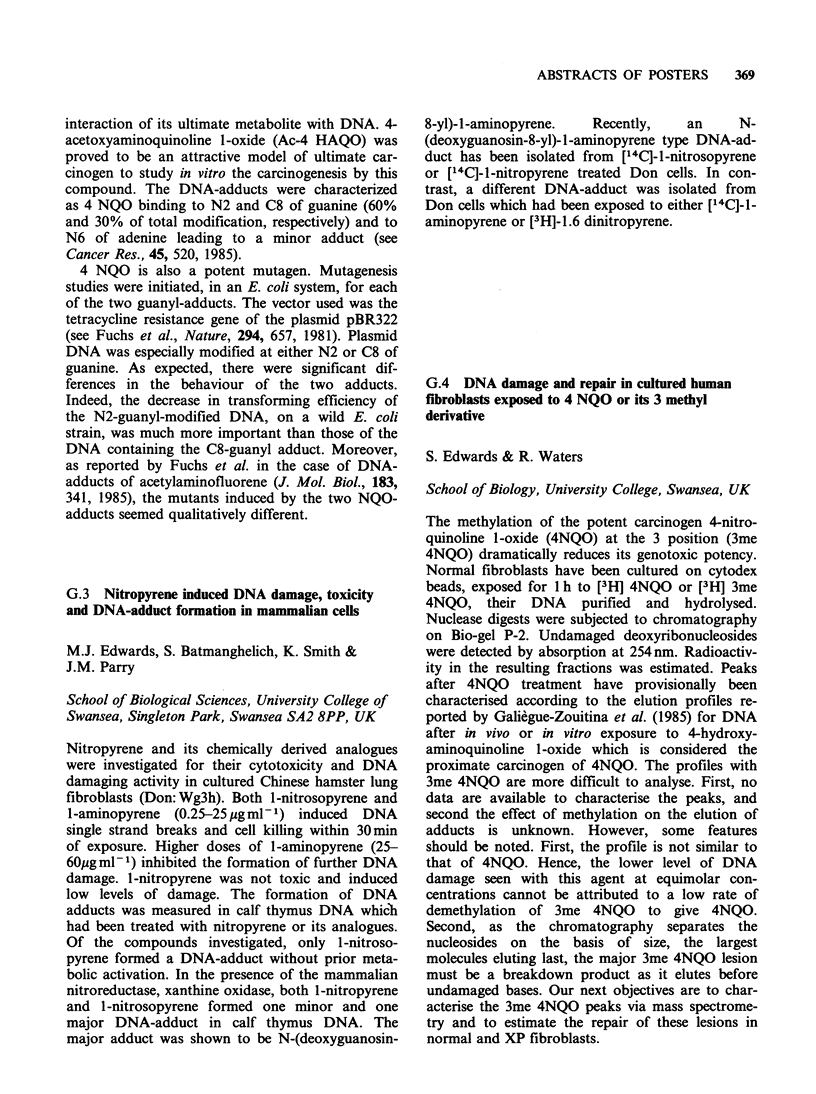

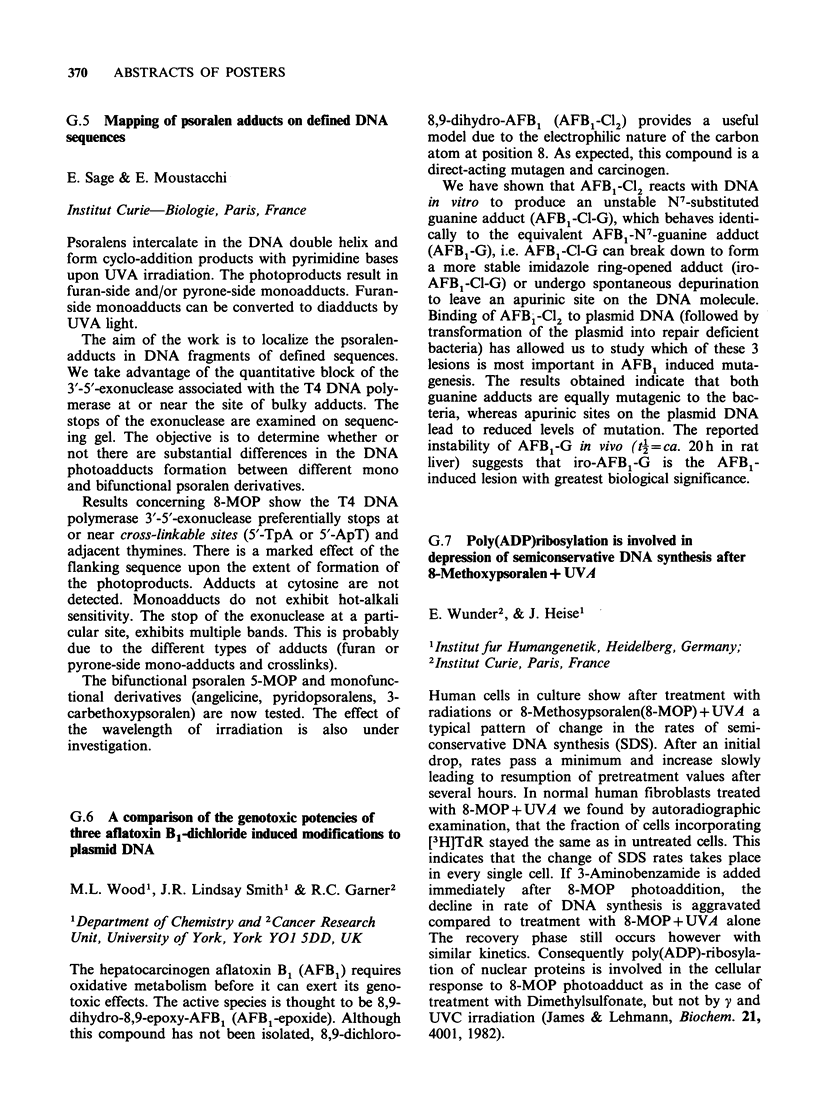

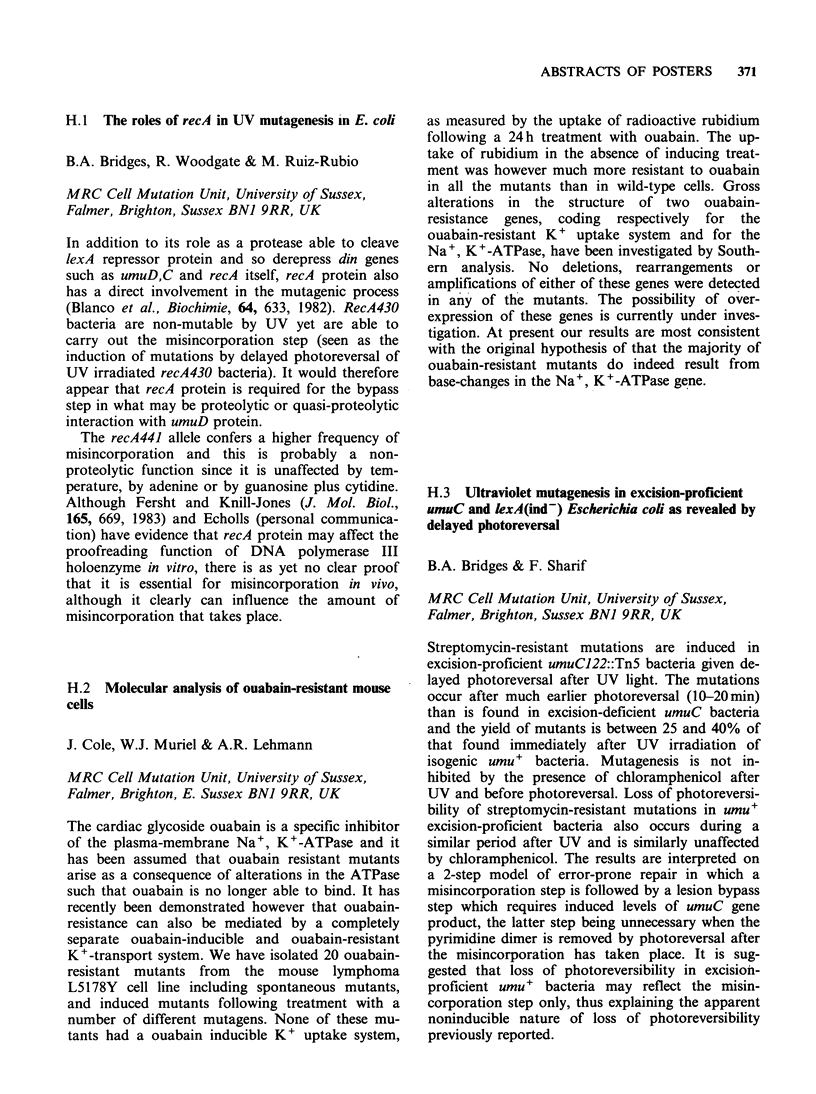

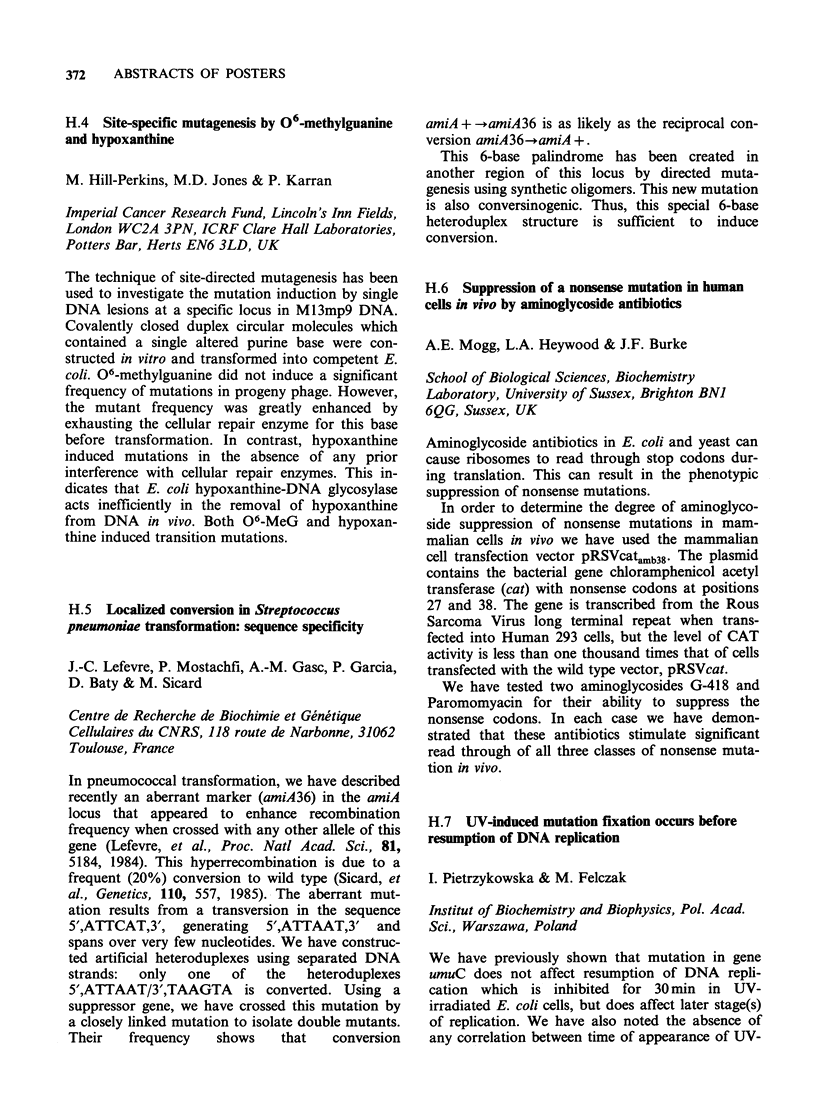

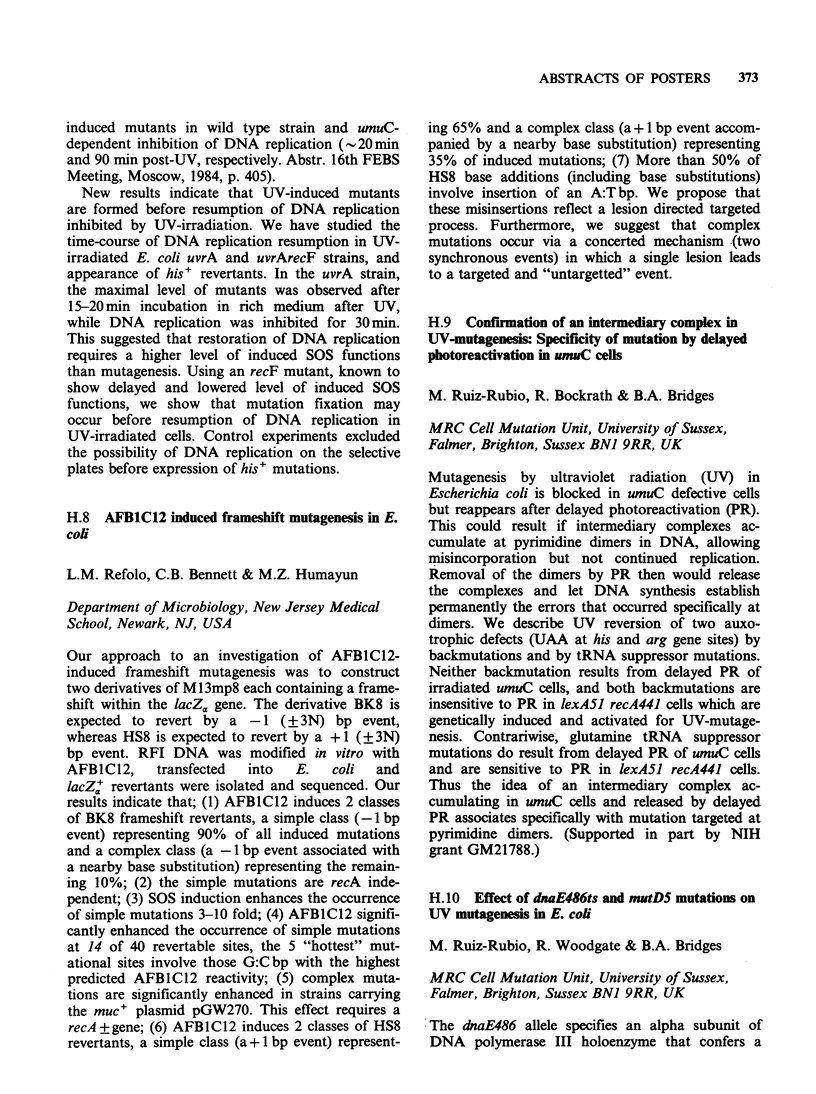

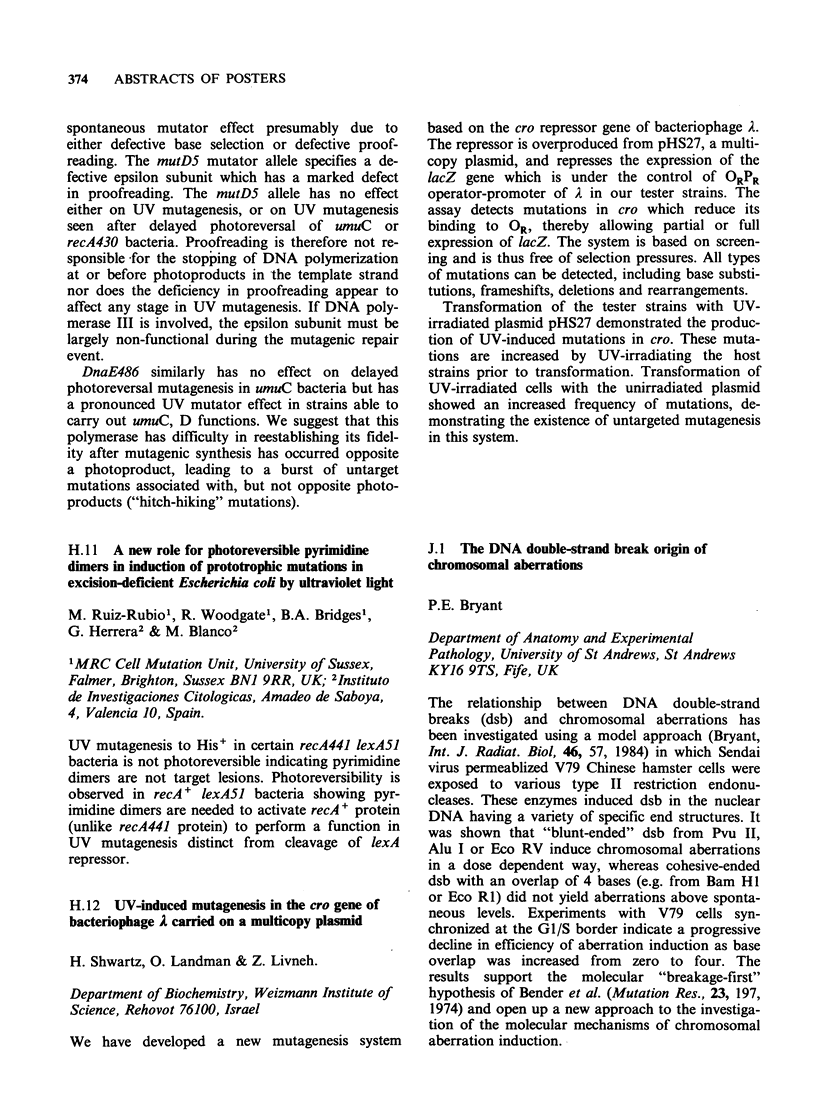

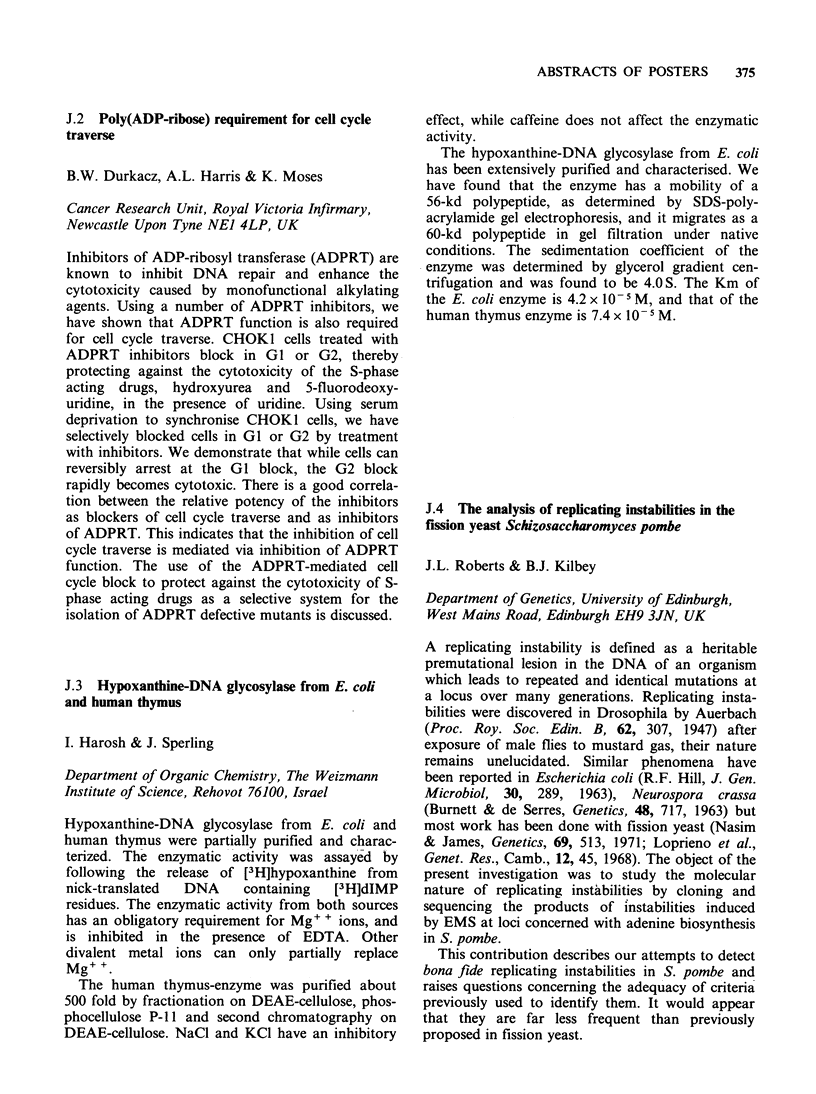

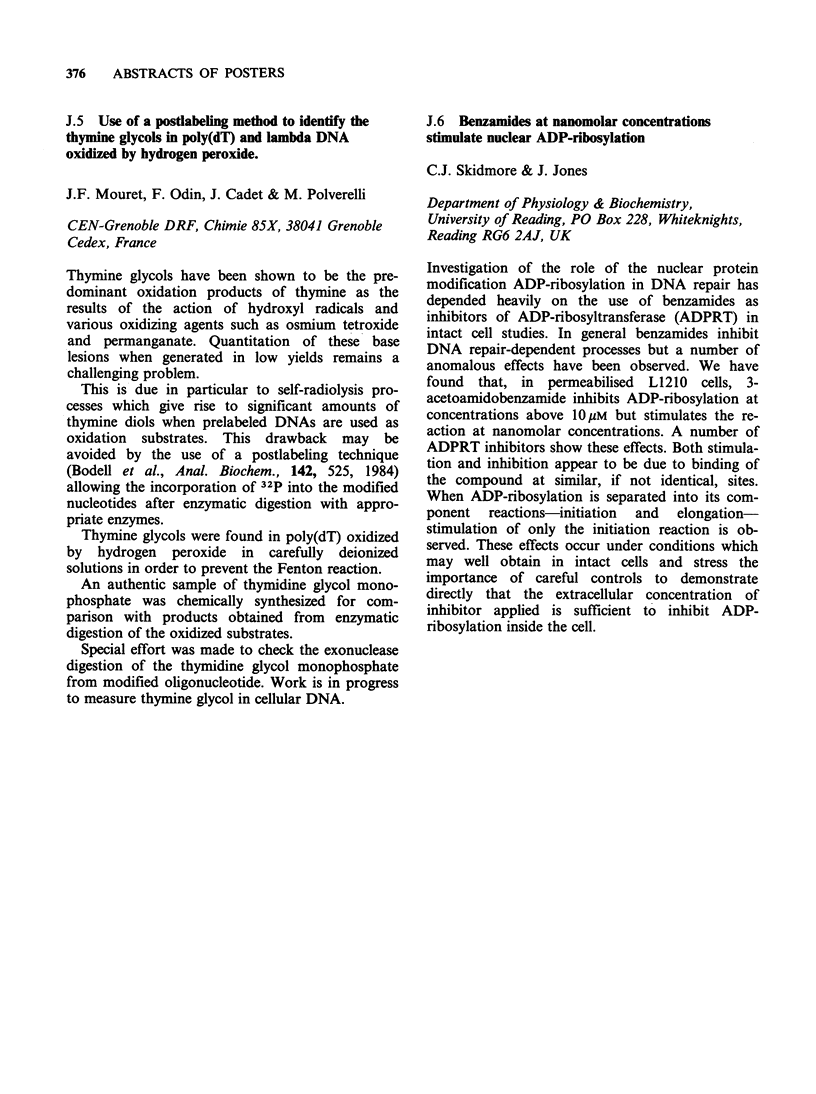


## References

[OCR_03543] BARNETT W. E., DE SERRES F. J. (1963). Fixed genetic instability in Neurospora crassa.. Genetics.

[OCR_00631] Bennett A., Berstock D. A., Carroll M. A. (1982). Increased survival of cancer-bearing mice treated with inhibitors of prostaglandin synthesis alone or with chemotherapy.. Br J Cancer.

[OCR_00637] Bennett A., Carroll M. A., Melhuish P. B., Stamford I. F. (1985). Treatment of mouse carcinoma in vivo with a prostaglandin E2 analogue and indomethacin.. Br J Cancer.

[OCR_00643] Bennett A., Houghton J., Leaper D. J., Stamford I. F. (1979). Cancer growth, response to treatment and survival time in mice: beneficial effect of the prostaglandin synthesis inhibitor flurbiprofen.. Prostaglandins.

[OCR_00650] Bennett A., Stamford I. F., Unger W. G. (1973). Prostaglandin E2 and gastric acid secretion in man.. J Physiol.

[OCR_02788] Bichara M., Fuchs R. P. (1985). DNA binding and mutation spectra of the carcinogen N-2-aminofluorene in Escherichia coli. A correlation between the conformation of the premutagenic lesion and the mutation specificity.. J Mol Biol.

[OCR_03006] Blanco M., Herrera G., Collado P., Rebollo J. E., Botella L. M. (1982). Influence of RecA protein on induced mutagenesis.. Biochimie.

[OCR_03583] Bodell W. J., Rasmussen J. (1984). A 32P postlabeling assay for determining the incorporation of bromodeoxyuridine into cellular DNA.. Anal Biochem.

[OCR_03015] Fersht A. R., Knill-Jones J. W. (1983). Contribution of 3' leads to 5' exonuclease activity of DNA polymerase III holoenzyme from Escherichia coli to specificity.. J Mol Biol.

[OCR_00662] Fischer S., Struppler M., Böhlig B., Bernutz C., Wober W., Weber P. C. (1983). The influence of selective thromboxane synthetase inhibition with a novel imidazole derivative, UK-38,485, on prostanoid formation in man.. Circulation.

[OCR_03541] HILL R. F. (1963). The stability of spontaneous and ultraviolet-induced reversions from auxotrophy in Escherichia coli.. J Gen Microbiol.

[OCR_00669] Hennam J. F., Johnson D. A., Newton J. R., Collins W. P. (1974). Radioimmunoassay of prostaglandin F-2-alpha in peripheral venous plasma from men and women.. Prostaglandins.

[OCR_00675] Hewitt H. B., Blake E. R., Walder A. S. (1976). A critique of the evidence for active host defence against cancer, based on personal studies of 27 murine tumours of spontaneous origin.. Br J Cancer.

[OCR_00691] Honn K. V., Busse W. D., Sloane B. F. (1983). Prostacyclin and thromboxanes. Implications for their role in tumor cell metastasis.. Biochem Pharmacol.

[OCR_00681] Honn K. V., Cicone B., Skoff A. (1981). Prostacyclin: a potent antimetastatic agent.. Science.

[OCR_00697] Lee E. T., Desu M. M. (1972). A computer program for comparing K samples with right-censored data.. Comput Programs Biomed.

[OCR_03146] Lefèvre J. C., Gasc A. M., Burger A. C., Mostachfi P., Sicard A. M. (1984). Hyperrecombination at a specific DNA sequence in pneumococcal transformation.. Proc Natl Acad Sci U S A.

[OCR_00142] Lieberman R. P., Oishi M. (1974). The recBC deoxyribonuclease of Escherichia coli: isolation and characterization of the subunit proteins and reconstitution of the enzyme.. Proc Natl Acad Sci U S A.

[OCR_00702] Moncada S., Vane J. R. (1979). Arachidonic acid metabolites and the interactions between platelets and blood-vessel walls.. N Engl J Med.

[OCR_03545] Nasim A., James A. P. (1971). Replicating instabilities in yeast: evidence from single cell isolation.. Genetics.

[OCR_00721] Stork J. E., Dunn M. J. (1985). Hemodynamic roles of thromboxane A2 and prostaglandin E2 in glomerulonephritis.. J Pharmacol Exp Ther.

